# Next Generation Sequencing Analysis Reveals Segmental Patterns of microRNA Expression in Mouse Epididymal Epithelial Cells

**DOI:** 10.1371/journal.pone.0135605

**Published:** 2015-08-13

**Authors:** Brett Nixon, Simone J. Stanger, Bettina P. Mihalas, Jackson N. Reilly, Amanda L. Anderson, Matthew D. Dun, Sonika Tyagi, Janet E. Holt, Eileen A. McLaughlin

**Affiliations:** 1 Reproductive Science Group, School of Environmental and Life Sciences, Faculty of Science and IT, University of Newcastle, Callaghan, NSW, Australia; 2 School of Biomedical Sciences and Pharmacy, Faculty of Health and Medicine, University of Newcastle, Callaghan, NSW, Australia; 3 Australian Genome Research Facility Ltd, The Walter and Eliza Hall Institute, Parkville, VIC, Australia; University of Hyderabad, INDIA

## Abstract

The functional maturation of mammalian spermatozoa is accomplished as the cells descend through the highly specialized microenvironment of the epididymis. This dynamic environment is, in turn, created by the combined secretory and absorptive activity of the surrounding epithelium and displays an extraordinary level of regionalization. Although the regulatory network responsible for spatial coordination of epididymal function remains unclear, recent evidence has highlighted a novel role for the RNA interference pathway. Indeed, as noncanonical regulators of gene expression, small noncoding RNAs have emerged as key elements of the circuitry involved in regulating epididymal function and hence sperm maturation. Herein we have employed next generation sequencing technology to profile the genome-wide miRNA signatures of mouse epididymal cells and characterize segmental patterns of expression. An impressive profile of some 370 miRNAs were detected in the mouse epididymis, with a subset of these specifically identified within the epithelial cells that line the tubule (218). A majority of the latter miRNAs (75%) were detected at equivalent levels along the entire length of the mouse epididymis. We did however identify a small cohort of miRNAs that displayed highly regionalized patterns of expression, including *miR-204-5p* and *miR-196b-5p*, which were down- and up-regulated by approximately 39- and 45-fold between the caput/caudal regions, respectively. In addition we identified 79 miRNAs (representing ~ 21% of all miRNAs) as displaying conserved expression within all regions of the mouse, rat and human epididymal tissue. These included 8/14 members of *let-7* family of miRNAs that have been widely implicated in the control of androgen signaling and the repression of cell proliferation and oncogenic pathways. Overall these data provide novel insights into the sophistication of the miRNA network that regulates the function of the male reproductive tract.

## Introduction

Following their initial morphological differentiation, spermatozoa are released from the germinal epithelium of the testes in a functionally immature state, incapable of movement or any of the complex array of cellular interactions that are required for fertilization [1]. In all mammalian species studied to date, the acquisition of functional competence occurs progressively as the cells descend through the epididymis, a highly specialized region of the male reproductive tract linking the testis to the vas deferens. A hallmark of epididymal maturation is that the process is driven exclusively by extrinsic factors in the complete absence of nuclear gene transcription or *de novo* protein translation in the spermatozoa [2].

The first region of the epididymis that immature sperm encounter is that of the caput, wherein the cells are concentrated by a mechanism of resorption that rapidly removes almost all of the testicular fluid that enters the epididymis. As they leave this environment and enter the corpus epididymis, sperm begin to acquire the potential for both progressive motility and recognition of an ovum. These attributes continue to develop as the sperm move through the corpus, and reach an optimal level as they enter the cauda region where they are stored in quiescent state prior to ejaculation. Importantly, the epididymis is characterized by highly regionalized profiles of both gene and protein expression [3], that ultimately give rise to a dynamic intraluminal environment responsible for [4] promoting sperm maturation. While androgens and additional lumicrine factors synthesized in the testis have been implicated in coordinating the gene/protein expression along the length of the epididymis, the balance of evidence indicates that they alone cannot account for the complexity of the distinct intrasegmental microenvironments. Instead, elegant pioneering studies by the laboratories of Zhang [5–10] and Sullivan [11–13] have provided evidence that an additional tier of regulation involving specialized RNA molecules, termed microRNAs (miRNAs), may be active in the epididymal environment.

miRNAs are small single-stranded non-coding RNA molecules (~21−25 nucleotides) that form an integral part of a recently discovered RNA interference pathway. miRNAs are initially synthesized as primary transcripts (pri-miRNA) before being sequentially processed in the nucleus by the RNase DROSHA, and within the cytoplasm by the endoribonuclease DICER. This leads to the formation of a double-stranded mature miRNA, one strand of which is preferentially loaded into an effector miRNA-induced silencing complex (miRISC). In addition to the loaded miRNA, the miRISC complex comprises catalytic Argonaute (AGO) proteins that mediate the post-transcriptional regulation of target mRNAs. The fate of the targeted mRNA varies from increased degradation and hence translational repression, through to increased translation and protein synthesis, depending on both the complementarity of the miRNA—mRNA duplex and on the catalytic activity of the AGO proteins within the miRISC complex [14,15]. Recent advances in miRNA expression profiling have fuelled rapid growth in our appreciation of the tremendous number, diversity and importance of this mechanism of post-transcriptional gene regulation in development, disease and fertility [16].

Evidence secured by a number of independent groups indicates that miRNAs play an essential role in regulating the differentiation of spermatozoa in the testes, with inactivation of key genes such as *Dicer1* leading to a severe impact on the formation of mature germ cells [17–22]. However, an emerging body of evidence indicates that this role may also extend to the post-transcriptional regulation of gene expression within the epididymis. Indeed, complex profiles of several hundred miRNAs have been documented in the epididymis of species such as the human, rat, and bovine and several of these are significantly enriched and or unique to this tissue [8,12,23]. Furthermore, comparative profiling of epididymal miRNAs has revealed distinct temporal and spatial patterns of expression [9,12]. The notion that miRNAs may be critical in establishing the unique epididymal environment and thus play a prominent role in promoting sperm maturation, is further supported by elegant global and targeted miRNA manipulation strategies. In the former studies, conditional knock-out of *Dicer1* in the proximal epididymis has been shown to elicit a rapid dedifferentiation of the epithelium, perturbation of steriod signaling, altered lipid homestatis, and a concomitant loss of fertility [24,25]. Similarly, defects in sperm fertility have also been documented following pertubation of gene expression via the injection of a single miRNA analog (agomir) directly into the epididymis of adult rats [10]. Notwithstanding these data, global patterns of differential miRNA expression have not been explored in the mouse epididymis. Therefore, the aim of the present investigation was to conduct a systematic analysis of the expression profile of miRNAs and key elements of their processing machinery within the adult mouse epididymis and in so doing provide novel insights into miRNA control of sperm fertilizing potential in this species.

## Materials and Methods

### Reagents

Unless specified, chemical reagents were obtained from Sigma (St. Louis, Mo, USA) or Life Technologies (Carlsbad, CA, USA) and were of research grade. The following primary antibodies were purchased to characterize proteins of interest: rabbit polyclonal anti-DICER1 antibody (Cat # ab13502; Abcam, Cambridge, United Kingdom), rabbit polyclonal anti-androgen receptor (Cat # SAB4501575, Sigma), rat monoclonal α-AGO2 (Cat # SAB4200085, Sigma), goat polyclonal anti-IZUMO1 (Cat # sc-79543, Santa Cruz Biotechnology, CA, USA), and rabbit polyclonal anti-cytokeratin 8 (Cat # ab59400, Abcam). Alexa Fluor 488-conjugated goat anti-rabbit (A11008), 594-conjugated goat anti-rat (A11007) and 594-conjugated goat anti-rabbit (A11012) antibodies were purchased from Life Technologies.

### Ethics Statement

All experimental procedures were carried out with the approval of the University of Newcastle’s Animal Care and Ethics Committee (ACEC) (approval number A-2013-322), in accordance with relevant national and international guidelines. Inbred Swiss mice were obtained from a breeding colony held at the institutes’ Central Animal House and maintained according to the recommendations prescribed by the ACEC. Mice were housed under a controlled lighting regime (16L: 8D) at 21–22°C and supplied with food and water *ad libitum*. Prior to dissection, animals were euthanized via CO_2_ inhalation.

### Epididymal Epithelial Cell Isolation and Characterization

Immediately after adult male mice (8 weeks old) were euthanized, their vasculature was perfused with pre-warmed PBS to minimize the possibility of blood contamination. The epididymides were then removed, separated from fat and overlying connective tissue and carefully dissected into three anatomical regions corresponding to the caput, corpus and cauda. This material was then pooled and either subjected directly to RNA extraction and miRNA next-generation sequencing as described below to document the ‘whole epididymal tissue’ miRNA signature (three mice / replicate), or alternatively it was prepared for isolation of epididymal epithelial cells (nine—twelve mice / replicate). For the latter study, the bulk of the caudal spermatozoa were flushed from the lumen via retrograde perfusion with water-saturated paraffin oil. This was immediately followed by perfusion with modified Biggers, Whitten, and Whittingham media (BWW; [26]) composed of 91.5 mM NaCl, 4.6 mM KCl, 1.7 mM CaCl_2_•2H_2_O, 1.2 mM KH_2_PO_4_, 1.2 mM MgSO_4_•7H_2_O, 25 mM NaHCO_3_, 5.6 mM D-glucose, 0.27 mM sodium pyruvate, 44 mM sodium lactate, 5 U/ml penicillin, 5 μg/ml streptomycin, 20 mM Hepes buffer, and 3 mg/ml BSA, to remove any residual spermatozoa. Caput and corpus spermatozoa were removed by placing the tissue in a 500 μl droplet of BWW and making multiple incisions with a razor blade. Using a method adapted from Zuo *et al*. [27], the tissue was then washed free of spermatozoa by subjecting it to agitation, prior to being minced with forceps washed a further three times in sterile PBS and digested in 100 μg/ml trypsin (Promega, Madison, WI, USA) at 37°C for 30 minutes with vigorous shaking. Tissue clumps were collected by centrifugation (800 × g for 5 minutes) and digested in 1 mg/ml collagenase for 30 minutes with shaking at 37°C. The cells were subjected to a further centrifugation at 800 × g for 5 minutes and the supernatant was discarded. The cell pellet was then resuspended in Dulbecco's Modified Eagle Medium (DMEM) culture medium containing sodium pyruvate (1 mM), 10% FBS (v/v), penicillin (100 IU/ml), and streptomycin (100 μg/ml), before being filtered through a 70 μm cell strainer and incubated in 6-well plates at 32°C. After 4 hours of incubation, all non-epithelial cells (fibroblasts and muscle cells) were found attached to the base of the plate while the epithelial cells remained in suspension. These populations of isolated epithelial cells were washed in PBS and either fixed in 4% paraformaldehyde for immunocytochemical analysis or frozen at -80°C for downstream RNA isolation.

Enrichment of epididymal epithelial cells (>95%) was assessed by immunocytochemistry. For this purpose, isolated epithelial cells were fixed in paraformaldehyde (ProSciTech, Kirwan, QLD, Australia) and settled onto poly-l-lysine coated coverslips overnight at 4°C. All subsequent incubations were performed at 37°C in a humidified chamber, and all antibody dilutions and washes were conducted in PBS containing 0.1% Tween-20 (PBST). Fixed cells were permeabilized in 0.2% Triton X-100 / PBS for 10 minutes and blocked in 3% (w/v) bovine serum albumin (BSA) in PBST for 1 h. Slides were then sequentially probed with anti-androgen receptor and Alexa Fluor 594-conjugated secondary antibodies. After washing, the slides were dual labeled with FITC-conjugated peanut agglutinin (PNA, 5 μg/ml), a marker of the outer acrosomal membrane, and counterstained with 4’,6-diamidino-2-phenylindole (DAPI) before being mounted with antifade medium (Mowiol 4–88). Labeled cells were viewed on an Axio Imager A1 microscope (Carl Zeiss MicroImaging, Inc., Thornwood, NY) equipped with epifluorescent optics and images captured with an Olympus DP70 microscope camera (Olympus America, Center Valley, PA). Upon confirmation of target cell enrichment, populations of isolated epididymal cells were then subjected to RNA extraction as described below.

### Immunofluorescent Localization

In addition to isolated epithelial cells, whole mouse epididymal tissue was also paraformaldehyde fixed, embedded in paraffin and sectioned. Embedded tissue was dewaxed, rehydrated and subjected to antigen retrieval by boiling in 50 mM Tris-HCl (pH10) for 10 minutes. The tissue was then assessed for key components of the RNAi silencing machinery under similar conditions to those described for isolated cells. Briefly, tissue sections from three individual mice were labeled with anti-DICER and anti-AGO2 antibodies (diluted 1:150 in 1% BSA/PBS) overnight at 4°C. After incubation, the slides were washed three times, then sequentially incubated in goat anti-rabbit 488 Alexa Fluor and goat anti-rat 594 Alexa Flour (diluted 1:400 in 1% BSA/PBS) secondary antibodies for 1 h at 37°C. Sections were then washed and counterstained in DAPI before mounting in antifade reagent. Similarly, epididymal sections were labeled with anti-androgen receptor primary antibody (1:50) overnight at 4°C, followed by goat anti-rabbit 594 Alexa Fluor (1:400). Negative controls included slides in which the primary antibody / lectin was substituted with PBS. All sections were visualized as described for cells and, as anticipated, the negative controls showed no labeling.

### RNA Extraction and miRNA Next-Generation Sequencing

Total RNA was extracted from whole epididymal tissue and purified epididymal epithelial cells (caput, corpus and cauda) using a Direct-zol RNA MiniPrep Kit (Zymo Research Corporation, Irvine, CA, USA) according to manufacturer’s instructions before being incubated with 1% DNase (Promega) to eliminate genomic DNA contamination. Total RNA from each epididymal region was pooled from a minimum of three (whole tissue)—nine (isolated epithelial cells) animals to generate a single biological replicate. Two such replicates were subjected to Illumina TruSeq small RNA sample preparation protocol as per the manufacturers’ instructions (Illumina Inc. San Diego, CA, USA) at the Australian Genome Research Facility (AGRF, Brisbane, QLD, Australia). The libraries so generated were analyzed in triplicate sequenced using an Illumina Hiseq-2000 RNA-seq platform as 50 bp single end chemistry at AGRF. Briefly, the sequence reads from all samples were analyzed for quality control, screened for the presence of any contaminants and trimmed based on their matches to: PhiX, Adaptors, ChrM or Mouse rRNA. Cleaned sequence reads were then aligned against two different databases: (i) Mus Musculus genome (Build version mm10), and (ii) microRNA database (miRBASE release21 at http://www.mirbase.org/). Alignment against the mature miRNA sequences for mouse miRNAs were summarized and counts were recorded for known miRNAs.

RNA quality was assessed at multiple points during our analysis. Firstly, the integrity of total RNA was evaluated immediately after isolation by resolution of an aliquot of the sample on a denaturing agarose gel and assessment of 28S and 18S rRNA bands. Additional quality control was conducted independently at the AGRF whereby after arrival, each sample was analyzed on an Agilent 2100 Bioanalyzer as per the manufacturers’ instructions (Agilent Technologies, USA). Finally the samples were again analyzed after siRNA library construction to confirm the size of the products. Only samples that passed each quality control step were processed for next generation sequencing.

Differential miRNA expression analysis was undertaken using R script based on, limma and voom libraries (http://www.bioconductor.org/packages/release/bioc/vignettes/limma/inst/doc/usersguide.pdf). A count value of >10 was used as the cutoff for presence/absence and expression profiling comparisons were performed for mature miRNAs between the individual epididymal regions with a data filter set to ≥ 2 fold difference and false discovery rate (FDR) of 0.05. The data discussed in this publication have been deposited in NCBI's Gene Expression Omnibus [28] and are accessible through GEO Series accession number GSE70197 (http://www.ncbi.nlm.nih.gov/geo/query/acc.cgi?acc=GSE70197).

### Real Time PCR Confirmation of Selected miRNAs

Validation of miRNA expression profiles was conducted using a quantitative real-time PCR (qRT-PCR) strategy with Taqman miRNA assay reagents according to the manufacturer’s instructions (Life Technologies). The miRNAs selected for analysis were *let-7b-5p* (assay ID. 002619), *let-7c-5p* (assay ID. 000379), *miR-9-5p* (assay ID. 000583), *miR200c-3p* (assay ID. 002300), *miR-375-3p* (assay ID. 000564), *miR410-3p* (assay ID. 001274), *miR-470* (assay ID. 002588), *miR-467d-3p* (assay ID. 001826), and *miR486-5p* (assay ID. 001278). Real-time PCR was performed using a Light Cycler 96 SW 1.1 (Roche, Castle Hill, Australia). The U6 small nuclear RNA (snRNA) (assay ID. 001973) was employed as an endogenous control to normalize the expression levels of target genes, and relative expression levels were calculated using the 2^−ΔCt^ method [29]. All qRT-PCR assays were performed in triplicate using pooled biological samples (three mice / sample) differing from those employed for next generation sequence analyses.

### *In Silico* Analysis of miRNAs and Target Prediction

The expression of miRNA displaying statistically significant patterns of expression was clustered (Cluster3, Stanford University, Palo Alto, CA, USA) and examined using heatmaps (Java Treeview, Stanford University) to visualize trends and consistency in miRNA expression in caput, corpus and caudal epididymal epithelial cells. To gain a better understanding of the function of the up and down-regulated miRNAs their mRNA targets were analyzed with Ingenuity Pathway Analysis (IPA) software (version 8.8, Ingenuity Systems, Redwood City, CA, USA) using the Core Analysis. Similarly we also interrogated IPA in order to identify the key effected pathways likely to be regulated by epithelial cell miRNAs using the microRNA filter and restricting our analysis to experimentally confirmed targets.

### mECap transfection with miRNA Mimics

To confirm the functional significance of epididymal miRNAs, an immortalized mouse caput epididymal epithelial cell line (mECap) [30] was employed. This cell line was cultured in Dulbecco’s modified Eagle’s medium (DMEM; Life Technologies) supplemented with 0.45% glucose (w/v), 1% L-glutamine, 1% sodium pyruvate, 10% fetal bovine serum and 50 nM dihydrotestosterone (DHT). miRNA mimics of *miR-200c-3p*, *miR-486-5p* and a scrambled negative control (*mir*Vana) were transfected separately at a concentration 5 nM using lipofectamine 2000 along with a cherry red internal control (0.5 μg) in Opti-MEM (Life Technologies) as per manufacturer’s instructions. At 24 h post-transfection, cells were harvested and the transfection efficiency was calculated. The relative mRNA levels of predicted miRNA targets (*Mapk14* and *Foxo1*) were then analysed by qRT-PCR. These experiments were performed in triplicate.

### SDS-PAGE and Western Blotting

Proteins were extracted from epididymal tissue, isolated epididymal epithelial cells, and spermatozoa in a modified SDS-PAGE sample buffer (2% w/v SDS, 10% w/v sucrose in 0.1875 M Tris, pH 6.8) with protease inhibitor tablets by incubation at 100°C for 5 min. Insoluble matter was removed by centrifugation at 20000 × g for 10 min and protein estimations were performed using the DC Protein Assay kit (Bio-Rad, Hercules, CA). Proteins were boiled in SDS-PAGE sample buffer (2% v/v mercaptoethanol, 2% w/v SDS, and 10% w/v sucrose in 0.1875 M Tris, pH 6.8, with bromophenol blue) and resolved by SDS-PAGE on polyacrylamide gels followed by transfer onto nitrocellulose membranes. Membranes were blocked with 3% w/v BSA in Tris-buffered saline (TBS; pH 7.4) for 1 h before being probed with 1:1000 dilutions of primary antibody in TBS containing 1% w/v BSA and 0.1% v/v polyoxyethylenesorbitan monolaurate (Tween-20; TBS-T) for 2 h at room temperature. Blots were washed three times in TBS-T followed by incubation with 1:1000 horseradish peroxidase-conjugated secondary antibody in 1% w/v BSA/TBS-T for 1 h. Following three washes in TBS-T, proteins were detected using an enhanced chemiluminescence kit (GE Healthcare, Buckinghamshire, UK) and visualized on ImageQuant LAS 4000 (Fujifilm, Tempe, AZ, USA). All immunoblotting analyses were performed in triplicate and representative blots are presented.

### Statistical Analysis

Statistical significance was determined using analysis of variance (ANOVA), Tukey-Kramer HSD and T-tests employing JMP software (version 9.0.0). *P* < 0.05 was considered significant. Experiments were performed in triplicate unless otherwise stated. All data are expressed as mean ± SEM.

## Results

### miRNA Processing Machinery Is Expressed throughout the Mouse Epididymis

**Fig 1:**
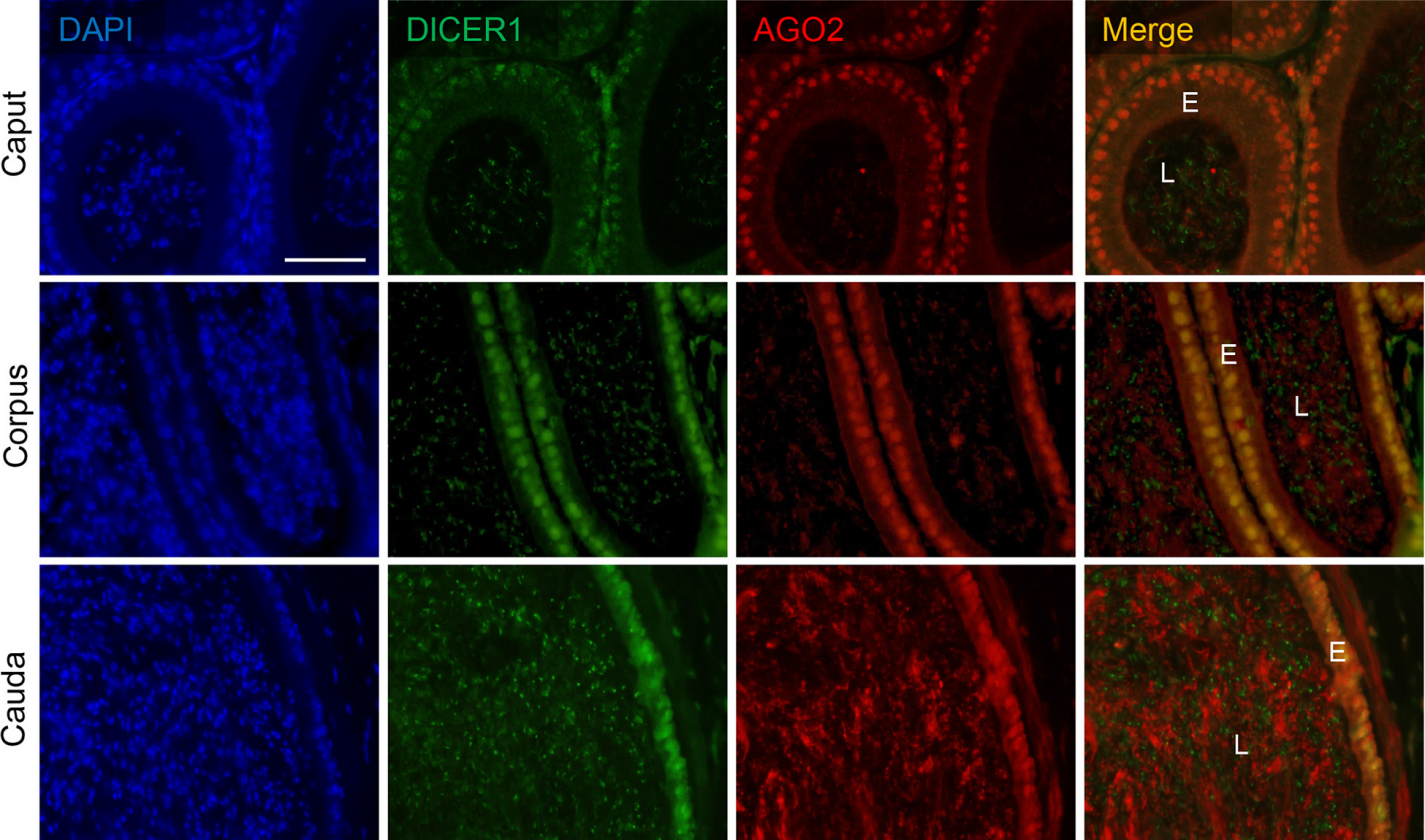
Assessment of key elements of the RNAi processing machinery in the mouse epididymis. Adult mouse epididymal sections were dual-labeled with anti-DICER1 and anti-AGO2 antibodies followed by either appropriate anti-rabbit 488 Alexa Fluor (green) or goat anti-rat 594 Alexa Flour-conjugated (red) secondary antibodies, respectively. The tissue sections were then counterstained with 4',6-diamidino-2-phenylindole (DAPI) and viewed using confocal microscopy. For clarity, DAPI labeling has been omitted from the merged images. E = epididymal epithelium, L = epididymal lumen. Scale bar = 20 μm. These experiments were replicated three times using independent samples from three mice and representative images are shown.

To begin our analysis of the role of the RNA interference pathway in the regulation of mouse epididymal function we initially focused on determining the expression profile of two of the key elements involved in the generation of mature miRNAs, namely the endoribonuclease DICER1 and the catalytic component of the RNA-induced silencing complex (RISC), Argonaute 2 (AGO2). As shown in Fig 1, both proteins were clearly detected in the epithelium of all epididymal regions examined, suggesting the presence of an active RNA interference pathway throughout the tubule. Indeed, both DICER1 and AGO2 displayed strong co-localization within the peri-nuclear domain of the epididymal epithelium. An additional pool of labeling, albeit of lower intensity, was also detected throughout the cytoplasm of these cells. In addition, both DICER1 and AGO2 appeared to strongly label spermatozoa within the epididymal lumen.

### The Mouse Epididymis Is Replete with miRNAs

**Fig 2:**
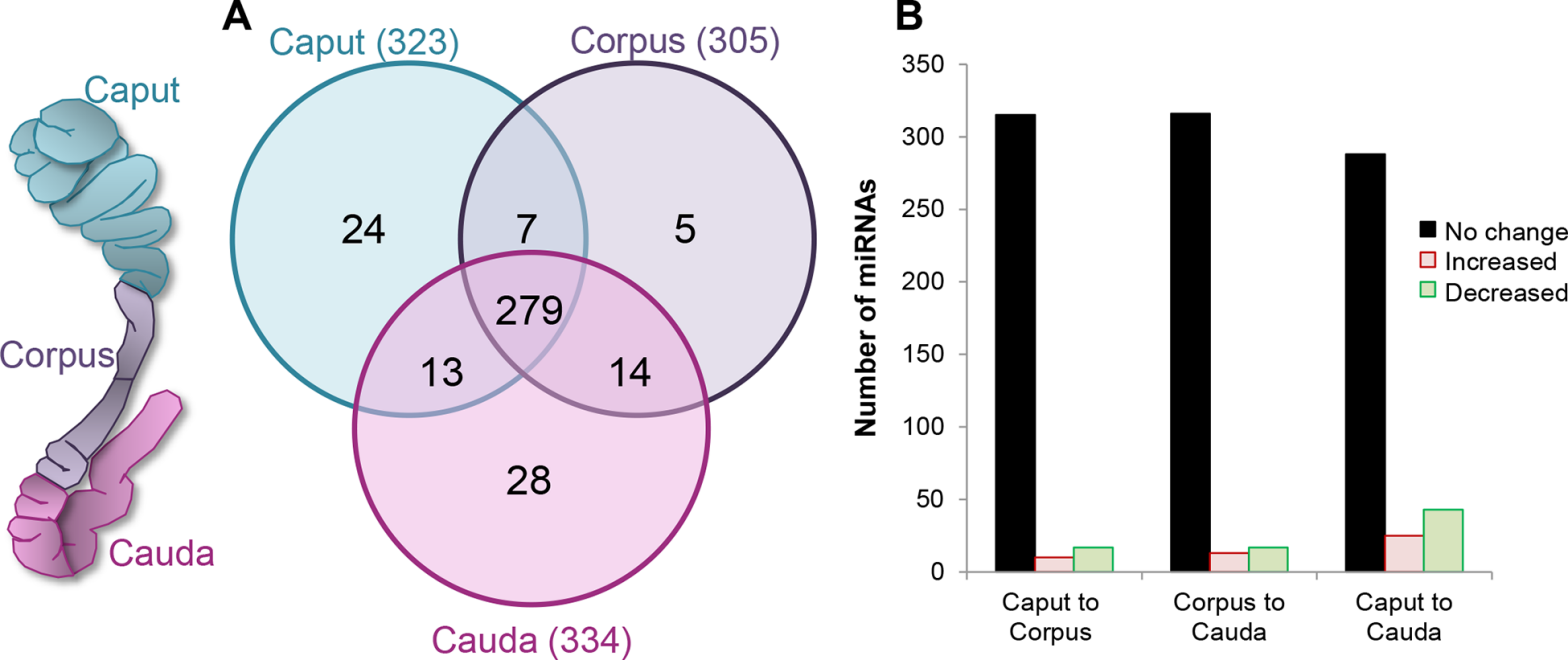
Determination of the miRNA signature present in whole mouse epididymal tissue. (A) Venn diagram depicting the number of miRNAs that were identified by next generation sequencing and their disruption within the caput, corpus and cauda regions of the adult mouse epididymis. (B) Graphical representation of miRNA distribution highlighting the number of significantly up- and down-regulated (threshold = ± ≥ 2 fold change and false discovery rate of < 0.05) miRNAs positively identified between each epididymal region. For the purpose of these analyses, an average count value of >10 across two biological replicates was used as the threshold for positive identification of each miRNA.

Next generation sequence analysis was performed on whole mouse epididymal tissue to obtain an overview of segmentally regulated miRNA expression patterns within this tissue. This analysis revealed a profile of miRNAs of similar overall complexity to that described in other species [9,11,12] (S1 Table). In total, 370 unique miRNAs were identified, the greatest number of which were detected within the cauda epididymal tissue, followed by the caput and finally the corpus (Fig 2A). A majority of these miRNA species (75%) were conserved in all epididymal regions and displayed relative levels of expression that were indistinguishable along the entire length of the epididymis. Indeed, only 15% of miRNAs were uniquely expressed in any one epididymal region (Fig 2A), and less than 10% were characterized by significant variations in their relative expression levels (fold change of ± ≥2, FDR <0.05) between the caput/corpus and corpus/cauda (Fig 2B).

**Fig 3:**
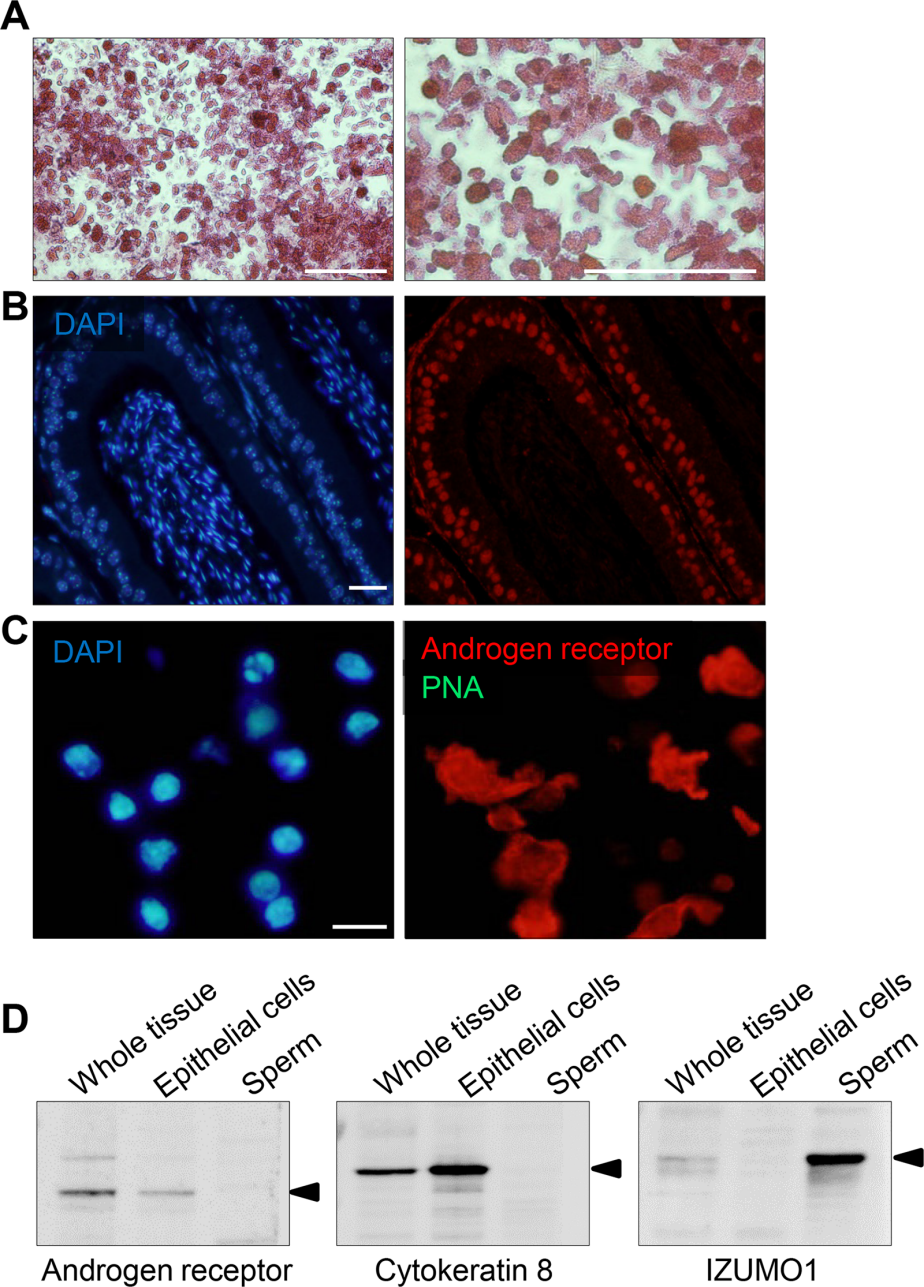
Isolation of highly enriched populations of epididymal epithelial cells. Epididymides were dissected from perfused adult mice and partitioned into the caput, corpus and cauda. The epithelial tissue was then cleared of spermatozoa, minced and sequentially digested in trypsin and collagenase. Epithelial cells were isolated by filtering through a 70 μm cell strainer and incubation in tissue culture plates prior to being fixed and assessed for overall purity by (A) staining with eosin and visualization by light microscopy (scale bar = 200 μm), (B,C) immunocytochemistry with anti-androgen receptor antibodies, and (D) immunoblotting with recognized epithelial / sperm cell markers. (B) The validity of androgen receptor antibodies for immunocytochemistry was initially assessed by labeling of mouse epididymal sections. These sections were then counterstained with DAPI and viewed using confocal microscopy (scale bar = 20 μm). (C) The identity of purified epithelial cells was confirmed by labeling with anti-androgen receptor and the possibility of sperm contamination assessed by co-staining with FITC conjugated PNA (a lectin that selectively labels the outer acrosomal membrane of spermatozoa). These preparations were then counterstained with DAPI (to detect all cells). This confirmed an absence of sperm contamination and epithelial cell enrichment of >95%. (D) As an additional line of evidence, cell lysates prepared from whole epididymal tissue (sperm + epithelial cells), enriched epithelial cells, and spermatozoa were immunoblotted with antibodies against androgen receptor (110kDa), cytokeratin 8 (an epithelial cell marker, 54kDa), and the sperm protein IZUMO1 (60kDa). All experiments were replicated three times on independent samples and representative images are presented.

The presence of so few intrasegmental changes in the miRNA profile detected in this, and previous analyses [12], stands in contrast to the pronounced spatial differences that have been documented in transcriptomic analyses of the epididymal tissue [3,31,32]. To investigate whether such differences reflect the potentially confounding influence of spermatozoa and/or fluid that convey miRNAs of testicular origin into the epididymal lumen, we refined our analysis to focus on miRNA signatures present exclusively in the epididymal epithelium. For this purpose, next generation sequencing was performed on highly enriched (>95%) populations of epididymal epithelial cells that were isolated as described in the Materials and Methods and initially validated through the use of light microscopy (Fig 3A) and immunocytochemistry with an anti-androgen receptor antibody (Fig 3B and 3C). As noted in Fig 3C, we did not detect any cells other than those expressing androgen receptor within this preparation. Indeed, the absence of sperm contamination was confirmed through counterstaining the slides with fluorescently conjugated PNA (marker of the sperm acrosome) (Fig 3C), and by immunoblotting of cell lysates with antibodies against the androgen receptor, cytokeratin 8, and IZUMO1 (an intrinsic sperm protein) (Fig 3D). As anticipated, no PNA labeling was observed within the epithelial cell preparations and similarly, no IZUMO1 protein could be detected in the epithelial cell lysates. In contrast, the epithelial cell lysates demonstrated strong labeling for both androgen receptor and cytokeratin 8.

**Fig 4:**
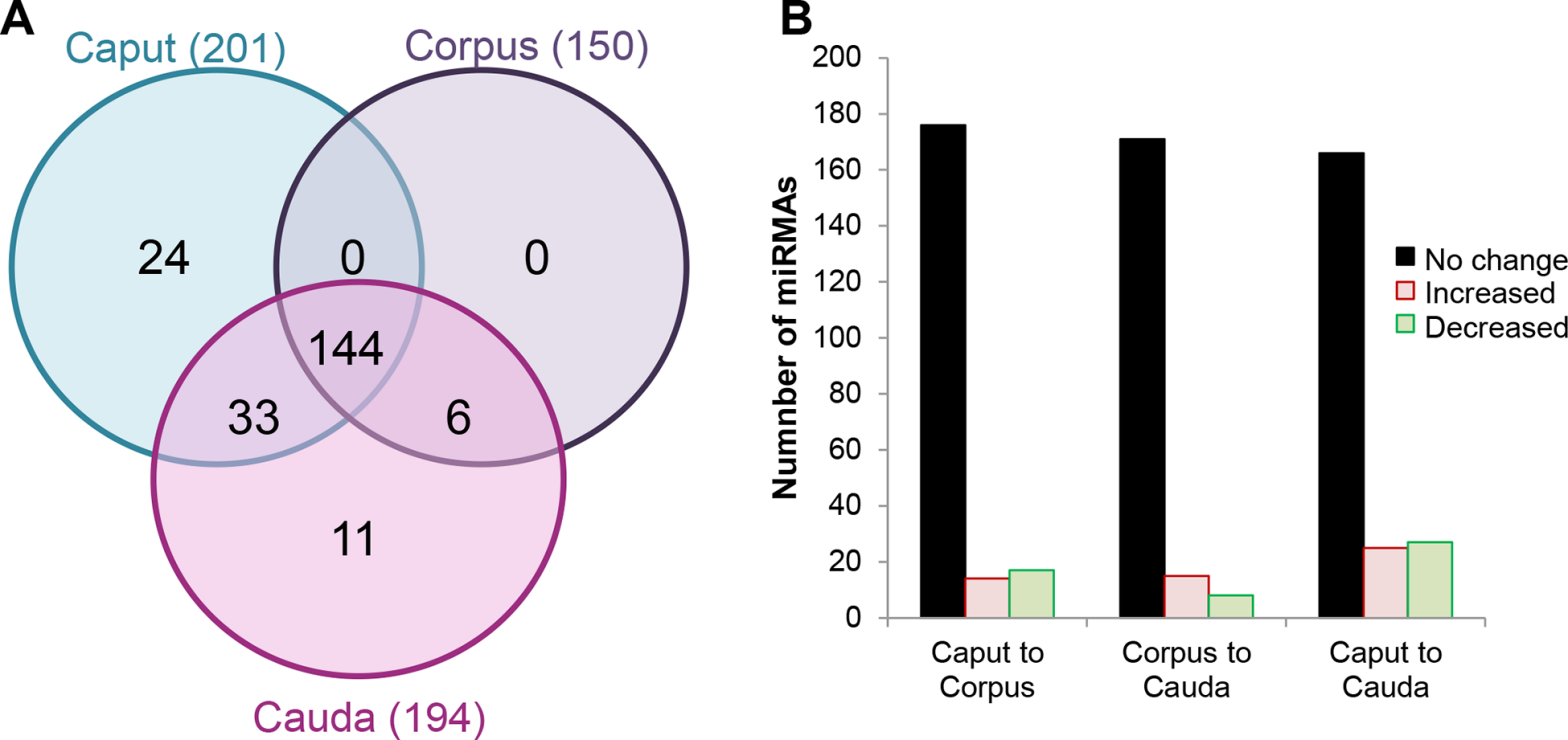
Determination of the miRNA signature present in enriched populations of epididymal epithelial cells. (A) Venn diagram depicting the number of miRNAs that were identified by next generation sequencing and their disruption within the epithelial cells of caput, corpus and caudal regions of the adult mouse epididymis. (B) Graphical representation of miRNA distribution highlighting the number of significantly up- and down-regulated (threshold = ± ≥ 2 fold change and false discovery rate of < 0.05) miRNAs positively identified in the epithelial cells of each epididymal region. For the purpose of these analyses, an average count value of >10 across two biological replicates was used as the threshold for positive identification of each miRNA.

**Fig 5:**
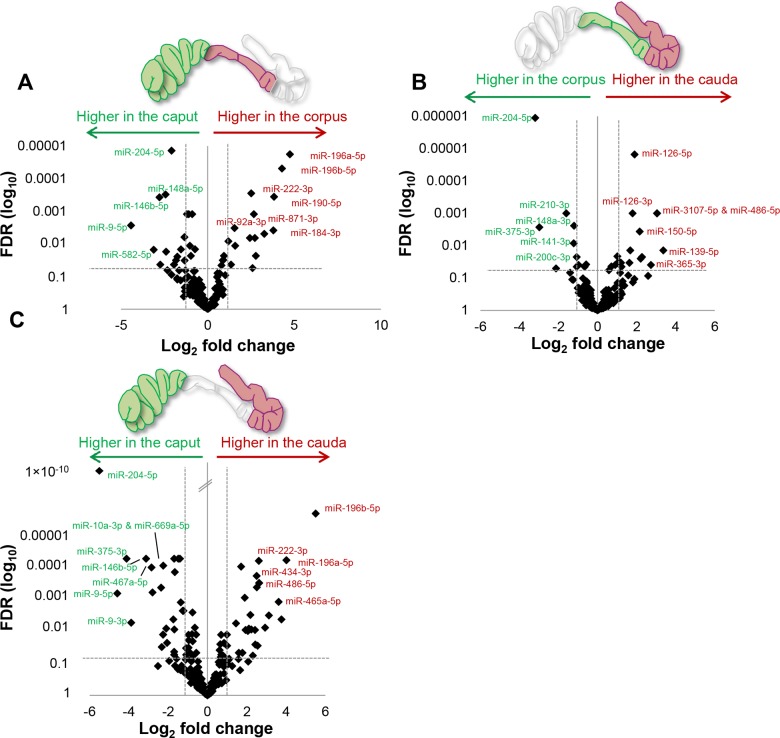
Volcano plots depicting the fold changes in miRNAs identified as being deferentially expressed within enriched populations of epididymal epithelial cells. Volcano plots highlighting the fold changes (x-axis) and false discovery rate (y-axis) of miRNAs that were identified as being differentially expressed in the epithelium between the (A) caput/corpus (B) corpus/cauda and (C) caput/cauda epididymis. Dotted lines depict thresholds values for significantly up- and down-regulated (± ≥ 2 fold change and false discovery rate of < 0.05) miRNAs identified between each epididymal region.

**Fig 6:**
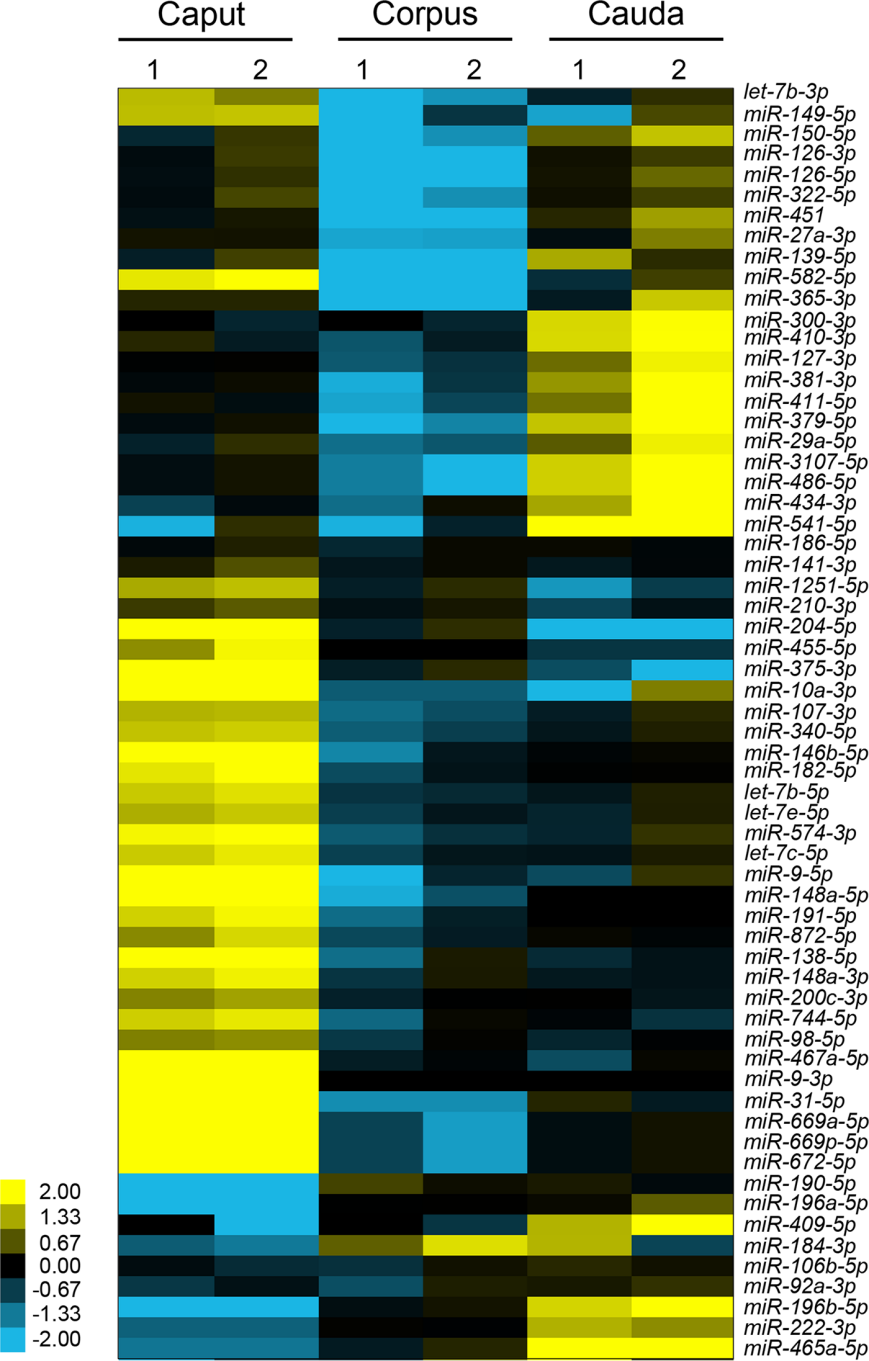
Analysis of the variability in miRNA expression between biological replicates. Hierarchical clustering of a subset of miRNAs that were identified as being differentially expressed between the epithelial cells from the caput/corpus, corpus/cauda, and caput/cauda was performed to assess overall variability between biological replicates. Cells within the matrix depict the relative expression level of a single miRNA within the 9 (caput/corpus)—12 (cauda) biological samples represented in each replicate. Yellow and blue shading represents the expression level (log_2_ fold change) above and below the median for this miRNA in all epithelial samples (caput, corpus, and cauda) analyzed, respectively.

Next generation sequencing of the isolated epithelial cell preparations led to the identification of a restricted subset of miRNAs (218), the majority of which (90%, 195) were also represented in the former analysis (S1 Table). Of the novel miRNAs detected in this assay, several (23) were present in low abundance raising the possibility that their signal may have been masked during the analysis of whole tissue preparations. Within the epithelial cell miRNA profile, two thirds (144) were conserved across all epididymal regions, with only 16% being exclusively expressed in any one epididymal region (Fig 4A). These results compare favorably with the data obtained for whole epididymal tissue, with a noteworthy feature of both analyses being that few miRNAs were uniquely detected in the corpus (5/370 and 0/218, respectively) (Figs 2A and 4A). Similarly, only a relatively small subset of miRNAs displayed expression levels that differed significantly between the caput/corpus and corpus/cauda (15% and 12%, respectively) (Fig 4B and S2 Table). More prominent changes were observed when comparisons were conducted over the entire length of the tract with almost a quarter (24%) of all miRNAs being characterized by significant variations in expression between the caput/cauda epididymis (Fig 4B and S2 Table). Although these data suggest that the spatial patterns of miRNA expression are not clearly demarcated by the broad intrasegmental boundaries of the caput, corpus and cauda, quantitative analysis of differentially expressed miRNAs revealed that many underwent substantial fold changes between the caput/corpus and corpus/cauda (Fig 5). Among the clearest examples of these, *miR-196b-5p* experienced a 45-fold increase in expression between the caput and caudal regions, while conversely *miR-204-5p* was down-regulated by approximately 39-fold over the same regions (Fig 5C). Importantly, we recorded consistent results across each biological replicate both in terms of the overall number of miRNA reads (S2 Table) and the relative fold change between regions (Fig 6).

**Fig 7:**
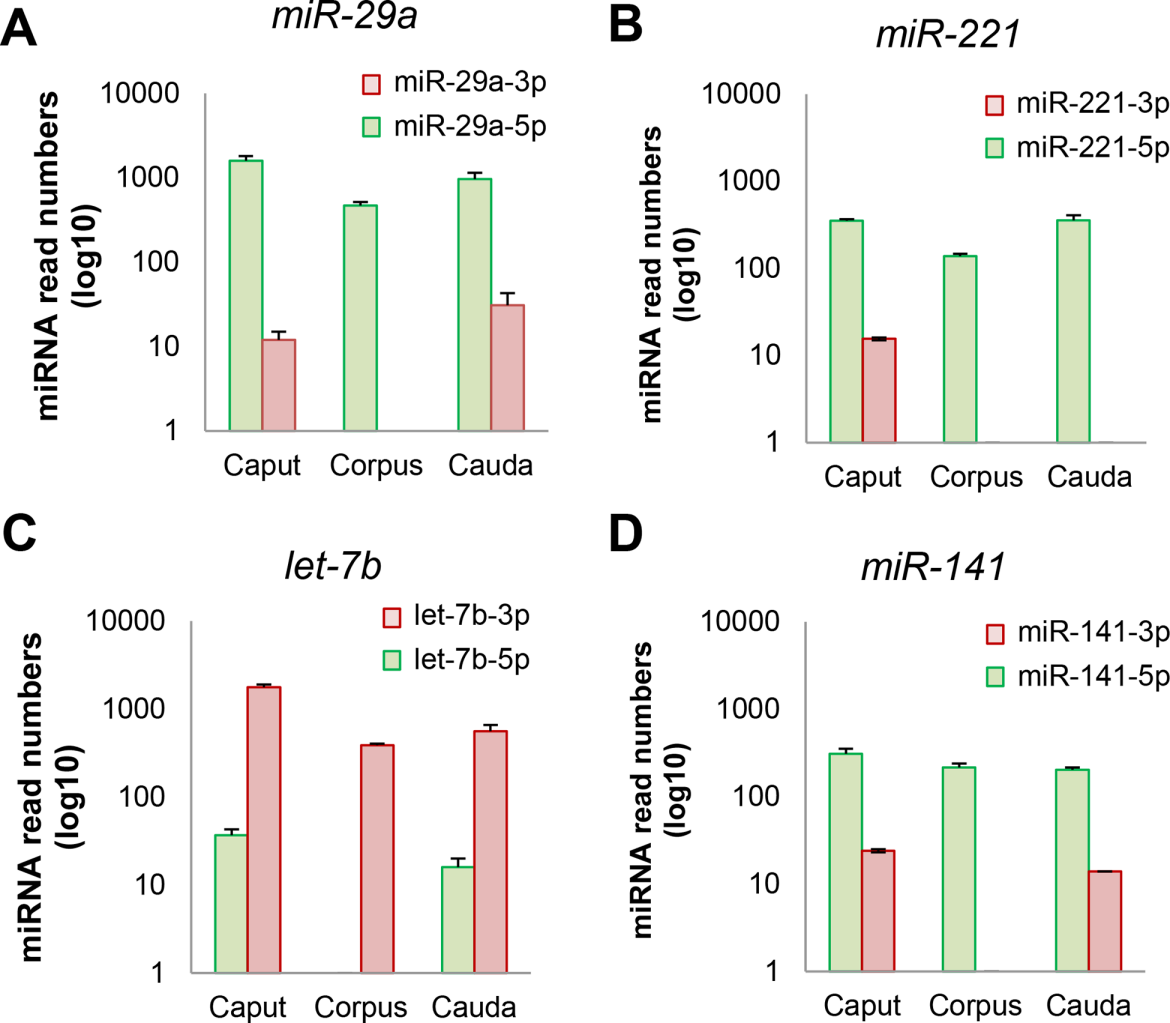
Determination of the relative expression levels of the mature products arising from representative miRNA precursors within the epididymis. Next generation sequencing revealed the relative expression levels of the two mature products arising from each miRNA precursor within the epididymis. Where products arising from both the 5′ and 3′ arm of a precursor mir hairpin were detected, one form generally dominated the expression profile throughout each epididymal region.

The deep sequencing strategy employed in the present study also afforded insight into the relative expression levels of the two mature products arising from each miRNA precursor within the epididymis (S1 Table). Where products arising from both the 5′ and 3′ arm of a precursor mir hairpin were detected, one form generally displayed dominant expression throughout each epididymal region (Fig 7). For instance, in the case of *miR-29a*, which has previously been implicated in androgen signaling within the mouse epididymis, the predominant product originated from the 3′ arm (*miR-29a-3p*) and was expressed at levels that were at least 30-fold higher than that of the alternative 5′ arm product (*miR-29a-5p*) (Fig 7A). These data accord with other tissues in which *miR-29a-3p* has also been shown to represent the predominant product generated from the *mir-29a* precursor (miRBASE).

### Validation of Differentially Expressed miRNAs

**Fig 8:**
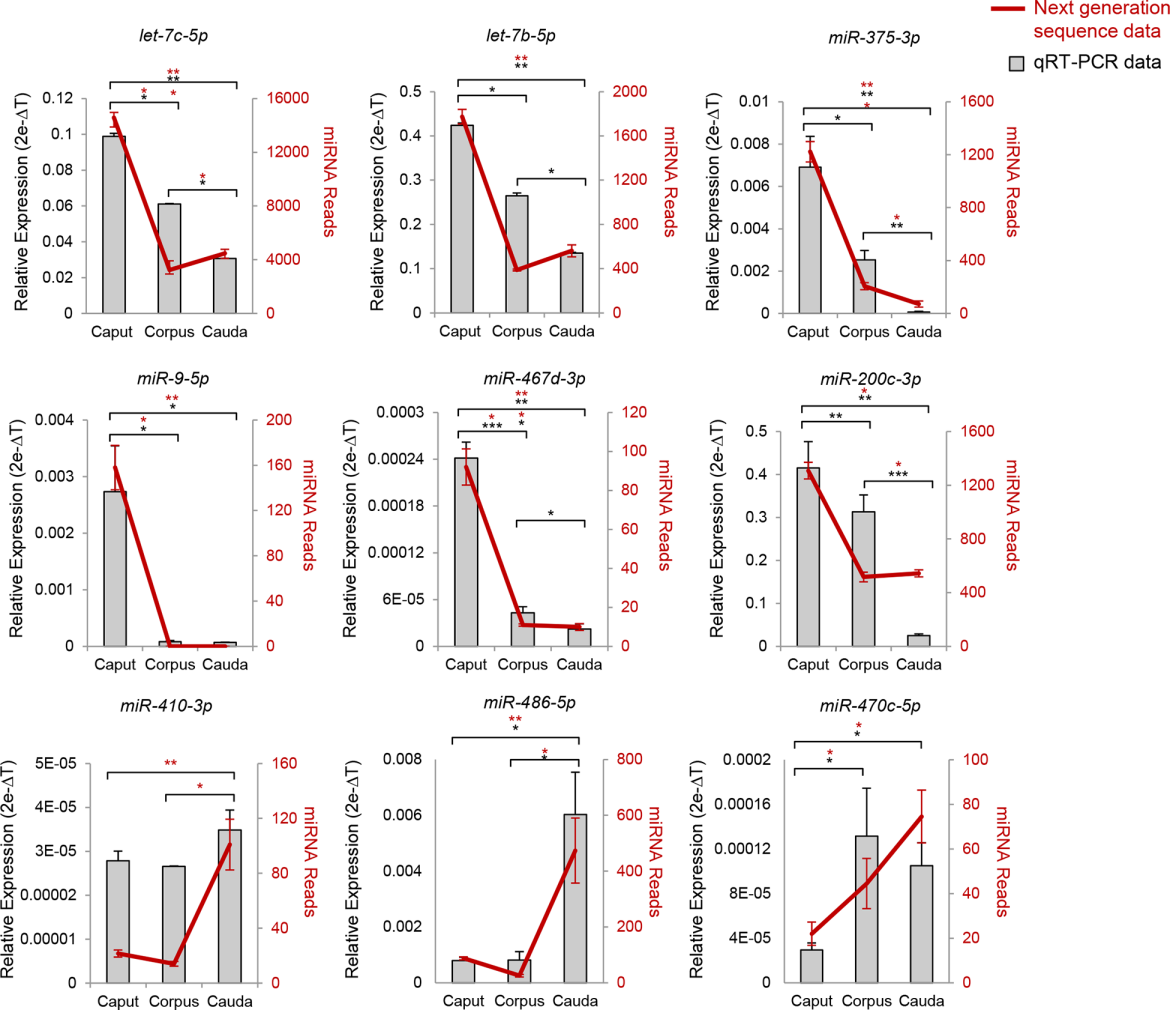
qRT-PCR validation of differentially expressed miRNAs within the mouse epididymis. In order to verify the next generation sequence data, nine differentially expressed miRNAs were selected for targeted validation using qRT-PCR, including representatives with highest expression in the proximal (caput: *let-7c-5p*, *let-7b-5p*, *miR-375-3p*, *miR-9-5p*, *miR-467d-3p*, and *miR-200c-3p*) and distal (cauda: *miR-410-3p*, *miR-486-5p*, and *miR470c-5p*) epididymis. qRT-PCR experiments were performed in triplicate using pooled biological samples (n = 3 mice / sample) differing from those employed for next generation sequence analyses. The U6 small nuclear RNA was employed as an endogenous control to normalize the expression levels of target miRNAs. * *P* < 0.05, ** *P* < 0.01, *** *P* < 0.001.

In order to authenticate the next generation sequence data, nine differentially expressed miRNAs were selected for targeted validation using qRT-PCR. These candidate miRNAs included representatives that exhibited regulated patterns of expression from each of the two primary classes detected, namely: those with highest expression in the caput (*let-7c-5p*, *let-7b-5p*, *miR-375-3p*, *miR-9-5p*, *miR-467d-3p*, and *miR-200c-3p*), or highest expression in the cauda (*miR-410-3p*, *miR-486-5p*, and *miR470c-5p*) epididymis. All qRT-PCR experiments were performed in triplicate using pooled biological samples (n = 3 animals/sample) that differed from those employed for next generation sequence analyses. In each experiment, the U6 small nuclear RNA was employed as an endogenous control to normalize the expression levels of target miRNAs. This analysis confirmed that eight of the nine target miRNAs were indeed differentially expressed within the epididymis (Fig 8). Furthermore, each of these eight targets displayed an expression profile that closely mirrored the trends identified by next generation sequence analysis (Fig 8). In this context, qPCR confirmed highly significant down-regulation of *let-7c-5p*, *let-7b-5p*, *miR-375-3p*, *miR-467d-3p*, and *miR-200c-3p* between the proximal and distal epididymal segments. It also highlighted the caput-specific expression of *miR-9-5p*, and confirmed a significant up-regulation of *miR-486-5p* and *miR470c-5p* between the caput and corpus epididymis. Only *miR-410-3p* failed to present significant changes in expression, however this result may be attributed to the relatively low absolute expression of this miRNA (Fig 8). Taken together, these findings suggest that our data faithfully report the spatial patterns of mouse epididymal miRNA signatures.

**Fig 9:**
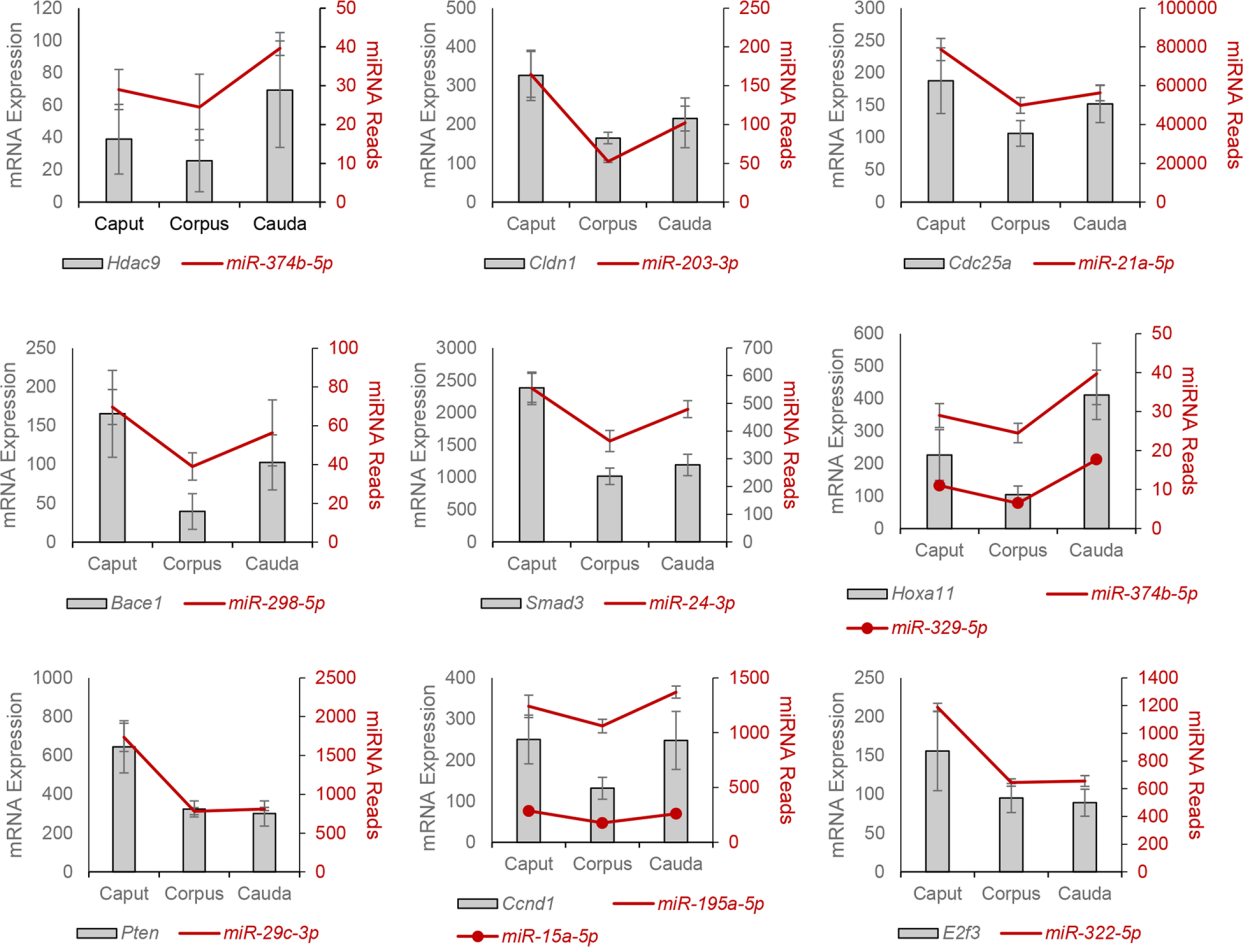
Correlation of miRNA expression profiles with that of their target mRNAs. An *in silico* analysis of published transcriptomic databases [3,31,32] was used to correlate miRNA and validated mRNA target (determined by IPA) expression levels throughout the epididymis. For the purpose of this comparison the mRNA expression levels reported in epididymal segments 2–5, 6–7, and 8–10 [3,31,32] were combined to represent overall expression in the caput, corpus, and cauda epididymis, respectively. A subset of 9 representative mRNA targets is depicted along with the relative levels of their targeting miRNA(s) as determined in the present study.

**Fig 10:**
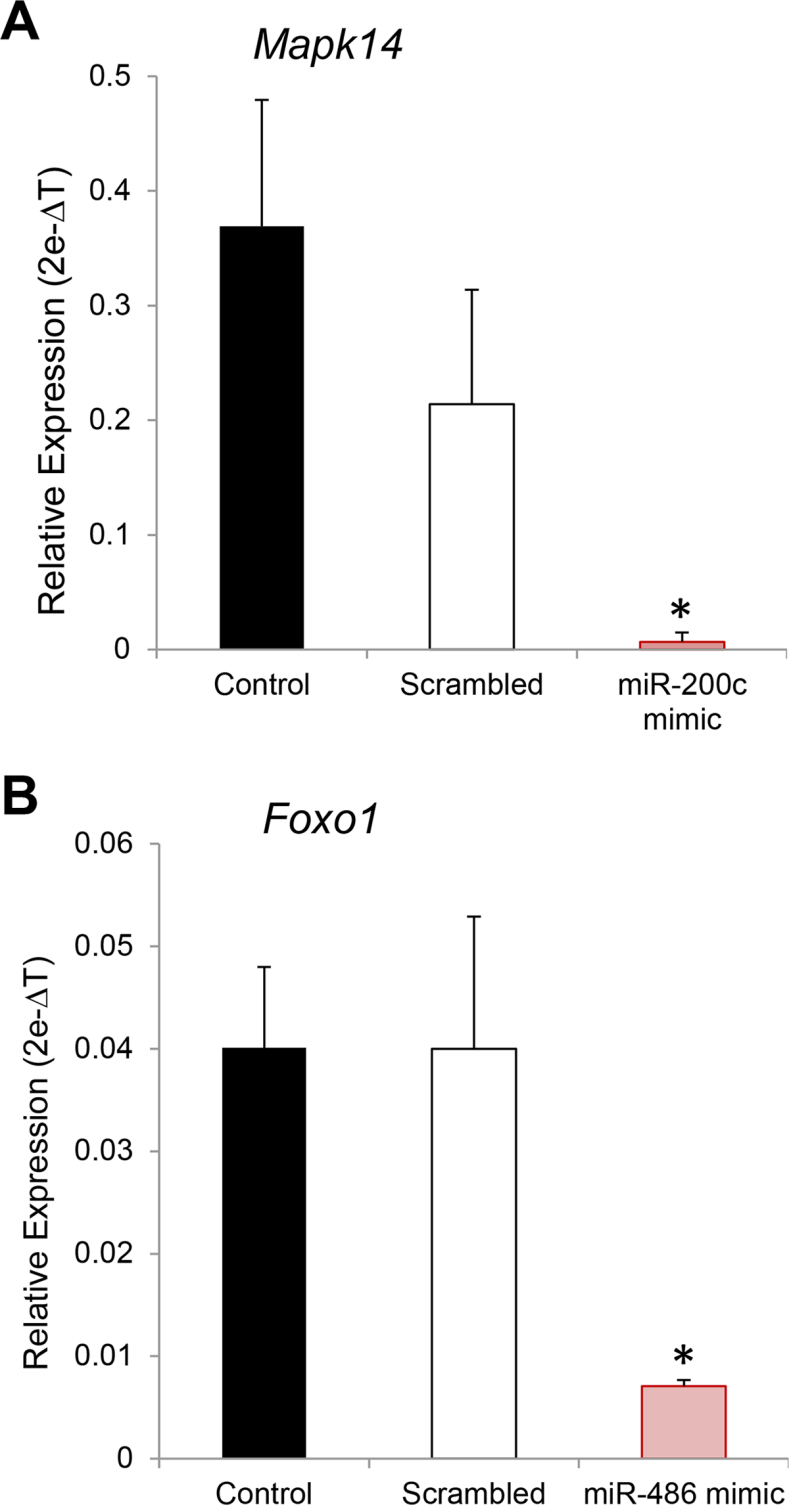
Examination of miRNA target gene knockdown. To confirm the functional significance of epididymal miRNAs, an immortalized mouse caput epididymal epithelial cell line (mECap) was co-transfected with a cherry red reporter and miRNA mimics of either (A) *miR-200c*, (B) *miR-486*, or a scrambled negative control (*mir*Vana). At 24 h post-transfection the relative levels of validated target mRNAs (*Mapk14* and *Foxo1*, respectively) were determined by qRT-PCR. These experiment were replicated three times and data are presented as mean ± SEM. * *P* < 0.05.

Having identified significant changes in miRNA expression within the epididymal epithelium, we next examined their functional significance by using an *in silico* analysis of published transcriptomic databases [3,31,32] to correlate miRNA and validated mRNA target expression levels throughout the epididymis. This approach proved effective at identifying several mRNAs whose relative levels of expression were positively correlated with that of their targeting miRNA(s), a subset of which are represented in Fig 9. These studies were therefore extended by employing a knockdown strategy in which an immortalized mouse caput epididymal epithelial cell line (mECap) was co-transfected with a cherry red reporter and miRNA mimics of either *miR-200c*, *miR-486* (shown to be significantly up-regulated in the caput and caudal regions, respectively), or a scrambled negative control (*mir*Vana). At 24 h post-transfection, cells were harvested, the transfection efficiency was calculated (25–70%) and the relative levels of validated target mRNAs determined by qRT-PCR. As shown in Fig 10 this strategy proved effective in eliciting a significant reduction in the expression of both *Mapk14* (targeted by *miR-200c*) (Fig 10A) and *Foxo1* (targeted by *miR-486*) (Fig 10B) mRNA. The specificity of this post-transcriptional knockdown strategy was confirmed by the absence of a similar effect in mECap cells transfected with *mir*Vana scrambled controls.

### The epididymal miRNA Signature Appears to be Poorly Conserved between Species

By exploiting the results of independent studies [7,12], we next sought to examine the level of conservation that exists between the miRNA signature of the mouse, rat and human epididymis. This survey focused on miRNAs identified in whole epididymal tissue (S1 Table) owing to the fact that previous global profiling studies have not been conducted on isolated epididymal cells. In addition, we were not able to distinguish between the two mature products arising from each miRNA precursor. Working within these limitations, and those associated with the alternative sequencing strategies based on commercially available miRNA microarrays that were employed in preceding work, our interrogation of known epididymal miRNAs revealed that only 21% (97/463) were conserved across the mouse, rat, and human (S3 Table). Interestingly, most of these conserved miRNAs (81%) were found to be expressed in the all epididymal regions (caput, corpus and cauda) in all species suggesting that they play fundamental house-keeping roles in the regulation of this tissue (S4 Table). As might be expected, the greatest level of similarity was observed in comparisons between the mouse and rat with approximately 42% of epididymal miRNAs identified in both species (143/341). In contrast, 31% (131/417) of miRNAs were detected in both the mouse and human epididymis. Interestingly, among the conserved miRNAs found in all epididymal regions, we identified 8/14 and 4/7 members of the *let-7* family (*let-7a—let-7f*, *let-7i*) and *miR-30* (*miR-30a*—*miR-30d*) family, respectively. The former family is of particular interest owing to its established role as a tumor suppressor and its high level of sequence and functional conservation across species [33]. Conversely, the 5 members of the *miR-888* cluster (*miR-890*, *miR-891a*, *miR-891b*, *miR-892a*, and *miR-892b*) that have been reported as being highly expressed in the corpus and caudal regions of the human epididymis [12] were not detected in our analysis of the mouse epididymis or in previous work on the rat epididymis [7].

This comparative analysis provided additional evidence for the differential expression of miRNAs in the epididymis of different species. For instance, *miR-32* and *miR-33* were found to be restricted to the caput epididymis of the mouse, while being absent in all regions of the rat epididymis, and present in the caput, corpus, and cauda of the human epididymis (S4 Table). Similarly, *miR-133b*, *miR-137*, *miR-155*, and *miR499* were exclusively expressed in the caudal region of the mouse epididymis but were widely distributed throughout the rat and/or human epididymis (S4 Table). Among a myriad of alternative interesting expression profiles, *miR-329* and *miR-350* displayed a biphasic pattern of expression in the mouse epididymis (being present in the caput and cauda, but absent in the corpus), but were entirely absent in the rat epididymis and present in all regions of the human epididymis (S4 Table).

### *In Silico* Analysis of miRNA Regulated Pathways in the Mouse Epididymal Epithelium

**Fig 11:**
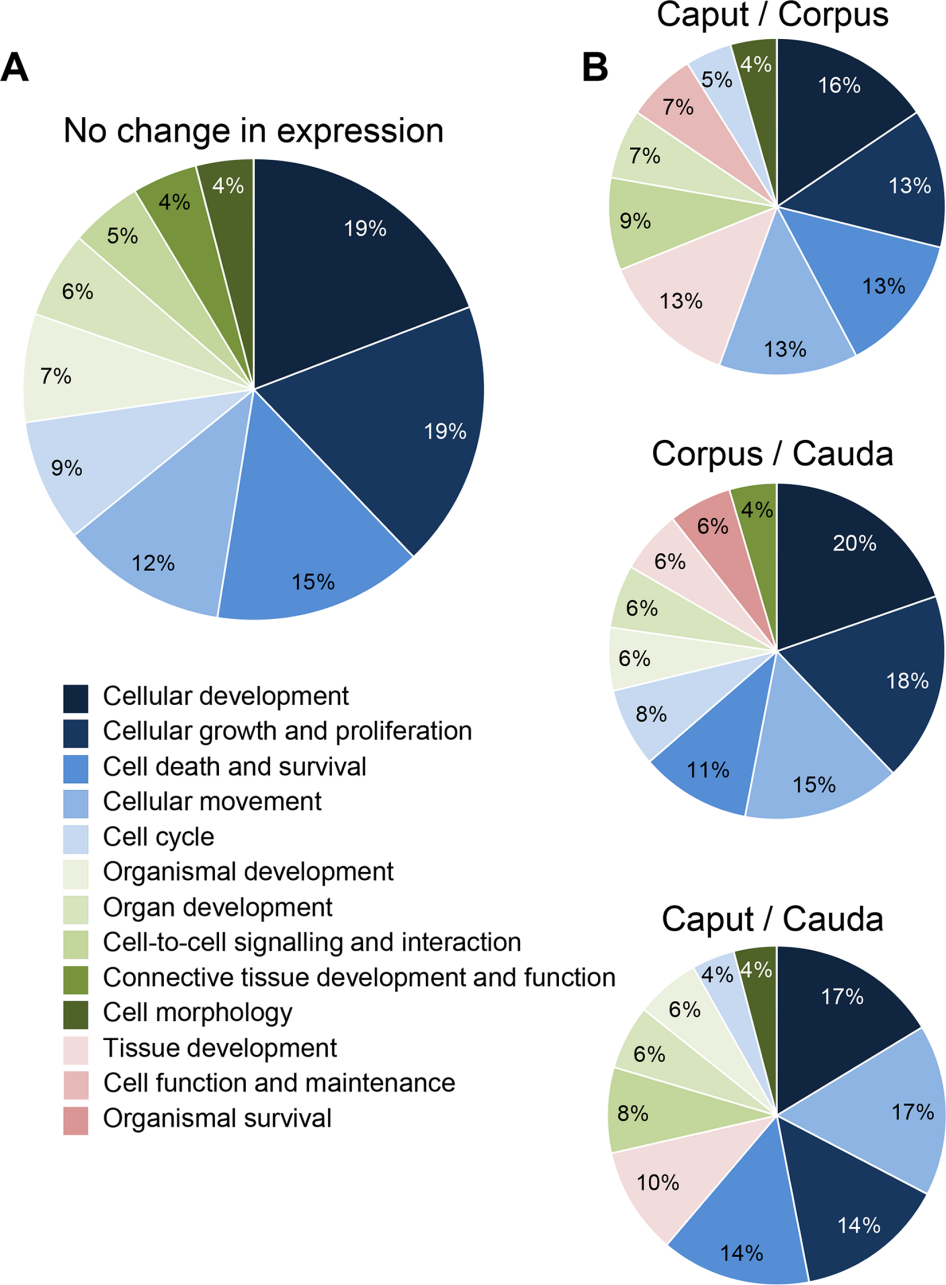
Key pathways regulated by epididymal miRNAs. Biological functions of miRNAs that were either (A) uniformly or (B) differentially expressed within the mouse epididymal epithelium were predicted through interrogation of Ingenuity Pathway Analysis (IPA) software. For both subsets of miRNAs, a majority of the experimentally validated target genes mapped to the broad categories of regulating cellular, tissue, and organ development, cell-cell signaling and interaction, and cell death and survival.

In order to gain an appreciation of the biological functions of miRNAs that are either uniformly or differentially expressed within the mouse epididymal epithelium, putative target genes were predicted through interrogation of Ingenuity Pathway Analysis (IPA) software using strict, experimentally validated filters. For both subsets of miRNAs, the putative target genes appeared to be involved in a range of biological processes with most of these mapping to the broad categories of regulating cellular, tissue, and organ development, cell-cell signaling and interaction, and cell death and survival (Fig 11A and 11B). Furthermore, within these categories we identified signaling cascades that are known to underpin key aspects of epididymal epithelium regulation and/or are implicated in sperm maturation/storage, suggesting that our analysis of miRNAs faithfully reported those of importance in epididymal function. For instance, among the 66 uniformly expressed miRNAs for which IPA assigned functions, we identified 12 candidates that have been implicated in androgen regulation, including: *let-7a-5p*, *miR-15a-5p*, *miR-17-5p*, *miR-19b-3p*, *miR-23a-3p*, *miR-24-3p*, *miR-27b-3p*, *miR-30a-5p*, *miR-34a-5p*, *miR-140-5p*, *miR-193a-3p*, *miR-205-5p* (S1 Fig). Similarly, within the differentially expressed pool of miRNAs, 10 were identified that are intimately involved in regulating intracellular trafficking pathways, including: *miR-7b-5p*, *miR-9-5p*, *miR-31-5p*, *miR-92a-3p*, *miR-106-5p*, *miR-126-3p*, *miR-150-5p*, *miR-204-5p*, *miR-222-3p*, *and miR-322-5p* (S2 Fig).

## Discussion

In all mammalian species studied, spermatozoa acquire the ability to fertilize an ovum during their passage through the epididymis [34]. A defining feature of this post-testicular maturation is that it is driven exclusively by the complex external milieu in which spermatozoa are bathed during their descent through the luminal environment of the epididymal tubule. Similarly, the prolonged storage of viable spermatozoa in the distal regions of the tract is also reliant upon the creation and maintenance of a highly specialized microenvironment. While the molecular mechanisms responsible for the controlling these dynamic regionalized environments are largely unresolved, recent evidence has highlighted a novel and potentially extremely important role for the RNA interference pathway in species such as the rat, bovine and human [8,9,35]. Since this additional tier of regulation has yet to be investigated in the mouse, we have employed next generation sequencing technology to define the global miRNA signature, and their segmental patterns of expression, in the epididymis of this important model species.

In samples of whole epididymal tissue we were able to identify a total of 370 miRNAs, representing one of the most comprehensive global screens of miRNAs performed in this tissue to date. However, a limitation of this initial analysis was that it failed to distinguish between the populations of miRNAs uniquely synthesized in each epididymal region as opposed to those that originate upstream within the testes and/or proximal epididymis before being delivered to the luminal compartment of more distal regions. In this context, it is well known that both spermatozoa [36–39] and epididymal secretions [11] harbor a complex repertoire of RNA populations, including miRNAs, that could potentially serve to mask important intrasegmental changes in the epididymal epithelial miRNA profile. Accordingly, restriction of our analysis to focus on highly enriched populations of epithelial cells dramatically narrowed the scope of the epididymal miRNA signature. Of the 218 miRNAs identified in this latter screen, comparatively few (<15%) were found to be differentially expressed between the caput/corpus and corpus/cauda epididymis. An important caveat to this finding is that our studies did not discriminate miRNA expression between the initial segment and caput epididymidis, two key regions that are known to play unique roles in murine epididymal physiology.

While our miRNA expression data stand in marked contrast to the regionalized patterns of gene [3,21,22] and protein expression [4,40–42] that are hallmarks of epididymal function, they do accord with the miRNA profiles documented in other species [7,12] and may thus reflect the fact that even small changes in miRNA expression can have a significant impact on the mRNA/proteomic profile of these cells. In keeping with this notion, it is well established that a single miRNA can influence the stability of potentially hundreds of mRNA targets thus setting the scene for a highly complex network of miRNA/mRNA interactions within epididymal cells. Indeed, even under strict target prediction criteria, an impressive array of some 8493 genes were identified as being putatively regulated by the differentially expressed miRNAs identified in this study. Specific examples include *miR-204-5p*, which was down-regulated by approximately 39-fold between the caput/caudal regions, and has an estimated 530 predicted gene targets in the mouse [43]. Interestingly, one of the key validated targets for *miR-204-5p* is SPARC (secreted protein acidic and rich in cysteine), a matricellular protein that regulates cell adhesion, matrix assembly and remodeling [44], and one whose spatial pattern of mRNA expression closely resembles that of *miR-204-5p*. Similarly, *miR-196b-5p* which displayed a 45-fold up-regulation in expression over the same regions, has been predicted to be target some 160 genes [43]. Foremost amongst these are the genes for the Homeobox (Hox) transcription factors that confer anterior-posterior axial coordinates during vertebrate embryo development [45], and have been implicated in regulating segmental function of the adult mouse epididymis [46,47]. Interestingly, several members of the *Hox* gene family whose expression has been confirmed in the mouse epididymis [3,47] are represented among the known targets for numerous additional miRNAs identified in the present study. One such example is *Hoxa11*, a transcript that displays highest levels of expression in the cauda followed by the caput and finally the corpus [3,47]. This spatial pattern of expression closely mirrors that of *miR-23a*, *miR-143*, and *miR-150*, all of which putatively target the *Hoxa11* mRNA.

While these data support the importance of miRNAs in fine tuning the regulation of the epididymal environment responsible for sperm maturation, an unexpected finding was the relatively poor conservation of miRNA signatures recorded between species. Indeed, at the level of sensitivity afforded by these analyses only 21% of epididymal miRNAs were detected in the mouse, rat, and human. We concede that these values may represent an under-representation of the true level of conservation, since our studies are confounded by the technical issues arising from the use of alternative sequencing technologies (next generation sequencing in the present study vs miRNA microarrays in previous work [7,12]) and/or the confounding influence of miRNAs harbored by luminal spermatozoa. However, we cannot discount the possibility that they report genuine differences in epididymal physiology between species. Support for this assertion rests with the data emerging from global transcriptomic [3,31,32] and proteomic analyses [4,40,41] that have been applied to the study of epididymal function. These genome-wide approaches have highlighted that significant species-specific differences do indeed exist in the epididymal transcriptome/proteome, with each species appearing to have developed unique strategies for promoting sperm maturation and preservation within this organ [40]. Although, there is a clear need for additional comparative studies to resolve these issues, a noteworthy feature of the majority of the miRNAs that were found to be conserved in the epididymis of the mouse, rat and human was their ubiquitous expression along the entire length of the tubule.

This conservation suggests that this subset of miRNAs form an integral part of the cellular processes responsible for the maintenance of epididymal homeostasis, with putative functions extending from promoting the development of the male reproductive tract, the regulation of sperm maturation, the maintenance of epididymal tight junctions and possibly the restriction of cellular proliferation within the adult organ. The latter is a defining feature of the epididymis and one that has been implicated in conferring an extraordinary resistance to primary cancer development, metastasis and invasive growth within this organ (reviewed by [48]). Indeed, human epididymal cancers account for only 0.03% of male cancers, an incidence rate that is well below that of other male reproductive tract malignancies (e.g. testicular cancer, 1.5% and prostate cancer, 20%). It also contrasts the occurrence of renal cancers (3%) despite the fact that the kidney and epididymis share a common embryonic origin (reviewed by [48]). Such observations have led to the proposal that the epididymis may possess intrinsic inhibitory mechanism(s) that protect the organ against the development of cancers, with recent work implicating miRNAs as master regulators of this process [49]. This assertion is supported by the observation that the *let-7* family of miRNAs, which have well characterized roles in the direct repression of cell proliferation and oncogenic pathways [33], featured prominently among the conserved epididymal miRNAs.

*Let-7* (*lethal-7*) is a founding member of the miRNA family that was originally described in *Caenorhabditis elegans*, where it controls the timing of terminal differentiation, acting as a key regulator of multiple genes required for exit from the cell cycle (reviewed by [33]). The *let-7* miRNA family has since been shown to display a remarkable level of sequence and functional conservation across the animal kingdom, with 14 and 13 different family members represented in mouse and human, respectively [33]. Among these members, *let-7a/b/c/d/e/f*, and *let-7i* are ubiquitously expressed in the adult mouse, rat and human epididymis. Of these family members, previous work has shown that 5 (*let-7a/b/c/d* and *let-7f*) are widely expressed in newborn, young adult, and aged human epididymides [8]. This is consistent with current models of *let-7* function which indicate that the family members are barely detectable during embryonic development before being up-regulated in differentiated cells where they are likely to possess highly redundant roles through targeting of an overlapping set of mRNAs [33]. Of significance to epididymal function, such roles are known to extend from the regulation of cellular proliferation through to the control of androgen receptor expression. For instance, *let-7c* has been reported to negatively regulate androgen receptor by suppressing its transcriptional activator c-Myc [50]. Similarly, dysregulation of *let-7* leads to a less differentiated cellular state and the development of cell-based diseases such as cancer [51,52]. It remains to be determined whether the 8 *let-7* family members that are expressed in the epididymis have different activities or whether they collectively target a similar cohort of genes. Nevertheless, it is tempting to speculate that the redundancy in *let-7* expression may contribute to the stringent molecular mechanisms that help the epididymis evade tumorigenesis. The prospect that a similar function may extend to other miRNAs is suggested by the conservation of several miRNAs (e.g. *miR-25*, *miR-34a/b/c*, *miR-135a/b*, *miR-194*, and *miR-200a*) that are capable of directly targeting the Wnt/β-catenin, a signaling pathway that has been widely implicated in the control of oncogenic hallmarks such as cell proliferation, metastasis, angiogenesis, telomerase activity, and apoptosis (reviewed by [49]). Indeed, aberrant activation and/or dysregulation of Wnt/β-catenin signaling has been identified as key underlying lesion in a significant portion of all human cancers (reviewed by [53]).

In summary, the data obtained in the present study provides novel insights into the diversity of miRNAs that are expressed within the mouse epididymis. Our findings accord with previous work emphasizing the sophistication of the miRNA network that coordinates the microenvironment necessary for post-testicular sperm maturation and storage. Interestingly however, despite the marked division of labor that characterizes epididymal function, we found that segment-specific miRNA expression is not a prominent theme in the mouse epididymis, with relatively few of the detected miRNAs displaying a significant difference in expression level between the three segments examined. Our systematic profiling of whole tissue and enriched populations of epithelial cells also served to identify luminal spermatozoa and/or epididymal fluid as a major contributor to the overall epididymal miRNA signature. Accordingly, our future studies will focus on the characterization of miRNAs in the luminal environment in order to determine whether they too are integrated into the complex regulation of epididymal function. Ultimately this work promises to improve our understanding of male infertility and identify novel targets for male contraception.

## Supporting Information

S1 ARRIVE Checklist ARRIVE Guidelines Checklist. This file is the ARRIVE Guidelines Checklist.
(PDF)

S1 Fig miRNA control of androgen regulation in the epididymis. Twelve of the miRNAs that were identified as being expressed at similar levels throughout all epididymal regions were mapped as putative regulators of the androgen signalling pathway (IPA: miRNA filter, experimentally observed).
(TIF)

S2 Fig miRNA control of endocytotic pathways in the epididymis. Ten of the miRNAs that were identified as being differentially expressed within the mouse epididymis were mapped as putative regulators of the clathrin mediated endocytosis (IPA: miRNA filter, experimentally observed).
(TIF)

S1 Table Comparison of miRNAs identified by deep sequencing within whole mouse epididymal tissue and highly enriched populations of epididymal epithelial cells. (PDF)

S2 Table Relative expression levels of miRNAs identified by deep sequencing within mouse epididymal epithelial cells. (PDF)

S3 Table Comparison of miRNAs identified within mouse, human and rat epididymal tissue. (PDF)

S4 Table Comparison of segmental expression of miRNAs identified within mouse, human and rat epididymal tissue. (PDF)
