# Supplementary figures and images for: Next Generation Sequencing Analysis Reveals Segmental Patterns of microRNA Expression in Mouse Epididymal Epithelial Cells
Source: PLoS One. 2015 Aug 13;10(8):e0135605. doi: 10.1371/journal.pone.0135605 (PMC4535982; doi:10.1371/journal.pone.0135605)

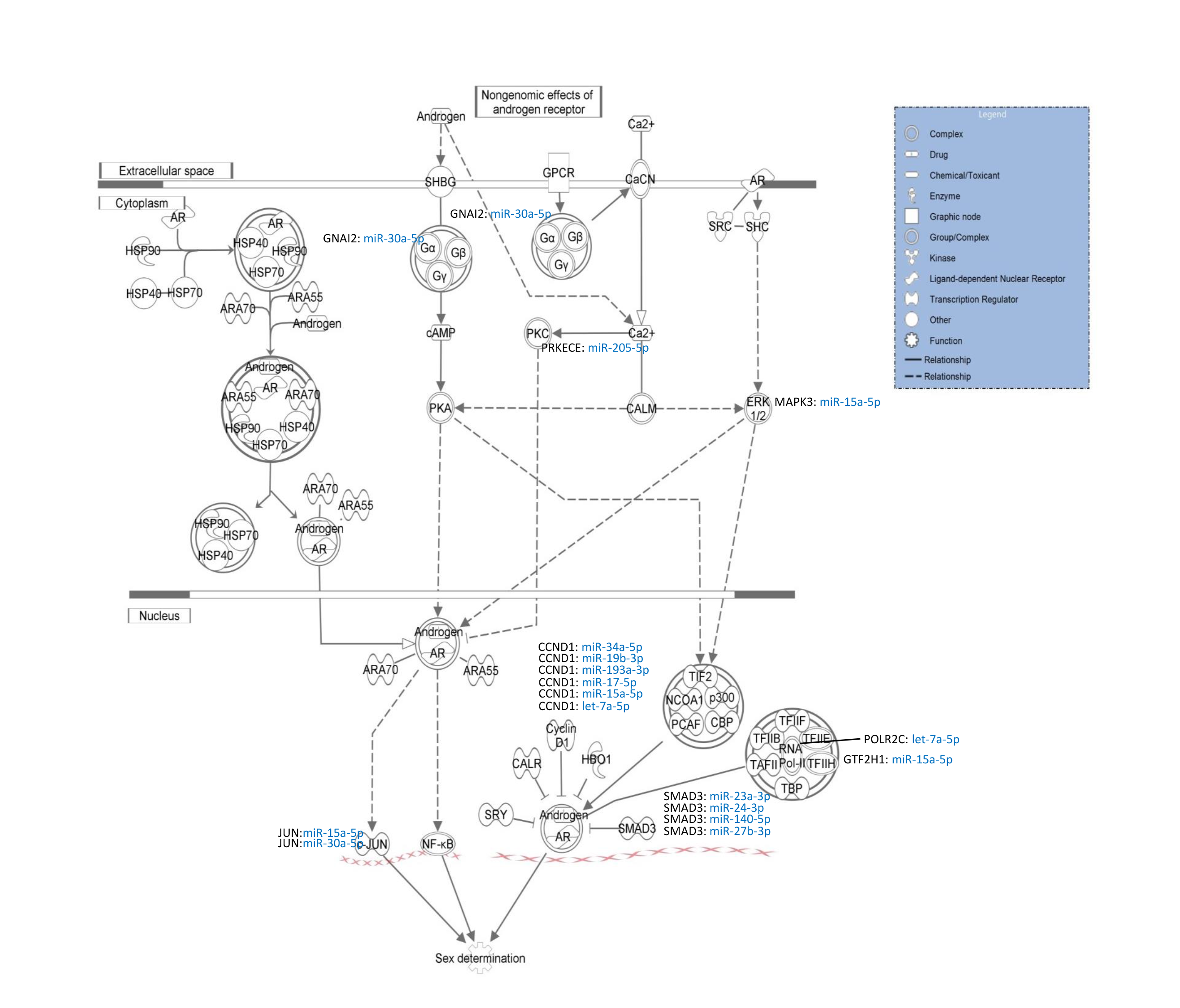

Supplement: S1 Fig — Twelve of the miRNAs that were identified as being expressed at similar levels throughout all epididymal regions were mapped as putative regulators of the androgen signalling pathway (IPA: miRNA filter, experimentally observed). (TIF) [file pone.0135605.s002.tif]

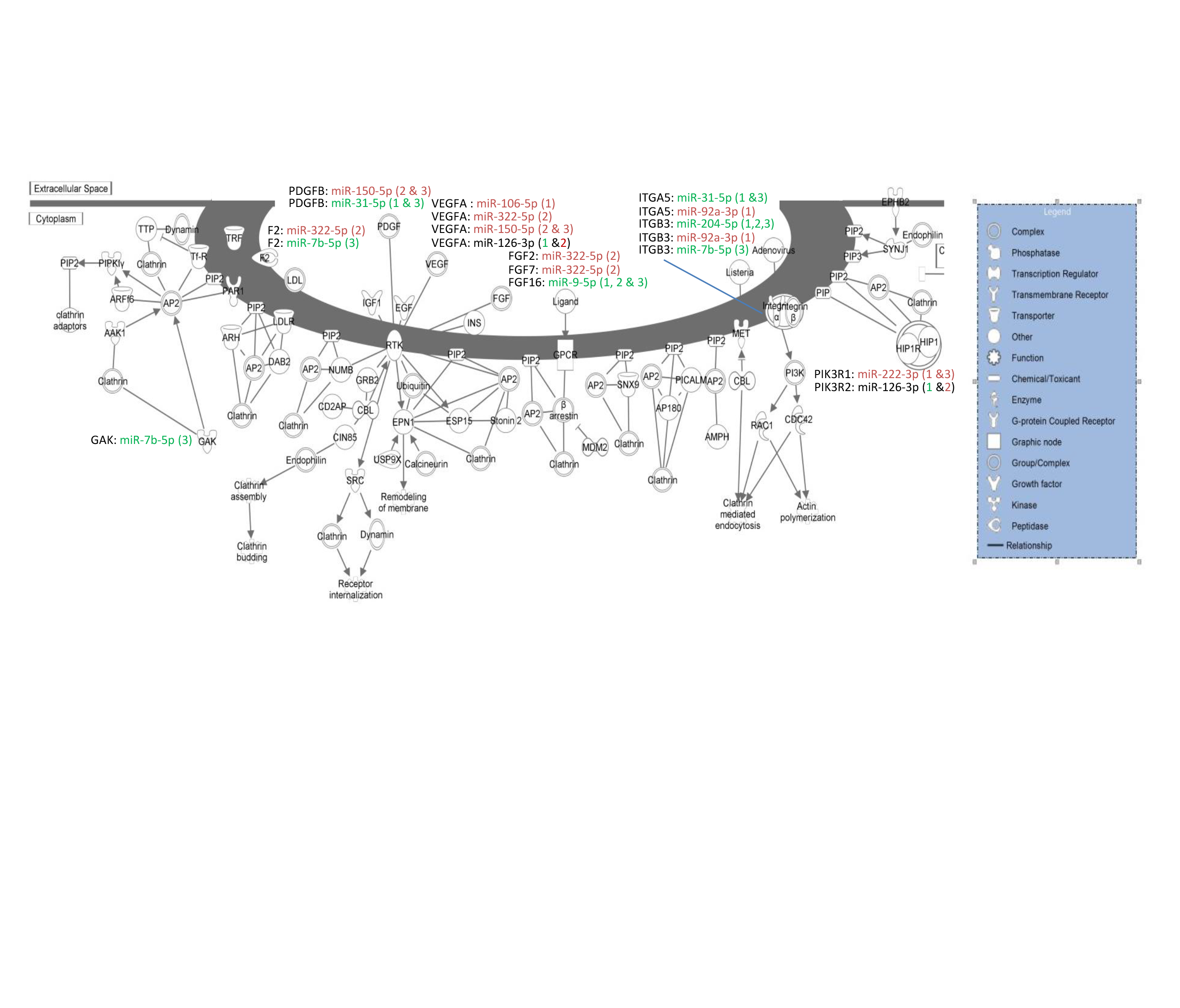

Supplement: S2 Fig — Ten of the miRNAs that were identified as being differentially expressed within the mouse epididymis were mapped as putative regulators of the clathrin mediated endocytosis (IPA: miRNA filter, experimentally observed). (TIF) [file pone.0135605.s003.tif]
