# Supplementary material for: Next Generation Sequencing Analysis Reveals Segmental Patterns of microRNA Expression in Mouse Epididymal Epithelial Cells
Source: PLoS One. 2015 Aug 13;10(8):e0135605. doi: 10.1371/journal.pone.0135605 (PMC4535982; doi:10.1371/journal.pone.0135605)
Supplement: S1 Table — (PDF) [file pone.0135605.s004.pdf]

S1 Table: Comparison of miRNAs identified by deep sequencing within whole mouse epididymal tissue and highly enriched populations of epididymal epithelial cells

MicroRNA Family	miRNA	Total Tissue (mmu)	Total Epithilial Cells (mmu)	Tissue (mmu)			Epithilial Cells (mmu)		
				Caput	Corpus	Cauda	Caput	Corpus	Cauda
let-7	*let-7a-1-3p*	+	+	+	+	+	+	-	+
	let-7a-5p	+	+	+	+	+	+	+	+
	let-7b-3p	+	+	+	+	+	+	-	+
	let-7b-5p	+	+	+	+	+	+	+	+
	let-7c-2-3p	+	+	+	+	+	+	-	+
	let-7c-5p	+	+	+	+	+	+	+	+
	let-7d-3p	+	+	+	+	+	+	+	+
	let-7d-5p	+	+	+	+	+	+	+	+
	let-7e-5p	+	+	+	+	+	+	+	+
	let-7f-5p	+	+	+	+	+	+	+	+
	let-7g-5p	+	+	+	+	+	+	+	+
	let-7i-5p	+	+	+	+	+	+	+	+
miR-9	*miR-9-3p*	+	+	+	-	-	+	-	-
	miR-9-5p	+	+	+	+	+	+	-	-
miR-10	*miR-10a-3p*	+	+	+	+	+	+	-	-
	miR-10a-5p	+	+	+	+	+	+	+	+
	miR-10b-5p	+	+	+	+	+	+	+	+
miR-15	*miR-15a-5p*	+	+	+	+	+	+	+	+
	miR-15b-5p	+	+	+	+	+	+	+	+
miR-16	*miR-16-5p*	+	+	+	+	+	+	+	+
miR-17	*miR-17-5p*	+	+	+	+	+	+	+	+
miR-19	*miR-19a-3p*	+	+	+	+	+	+	-	+
	miR-19b-3p	+	+	+	+	+	+	+	+
miR-20	*miR-20a-5p*	+	+	+	+	+	+	+	+
miR-22	*miR-22-3p*	+	+	+	+	+	+	+	+
	miR-22-5p	+	+	+	+	+	+	-	+
miR-23	*miR-23a-3p*	+	+	+	+	+	+	+	+
	miR-23b-3p	+	+	+	+	+	+	+	+
miR-24	*miR-24-2-5p*	+	+	+	+	+	+	+	+
	miR-24-3p	+	+	+	+	+	+	+	+
miR-25	*miR-25-3p*	+	+	+	+	+	+	+	+
miR-26	*miR-26a-5p*	+	+	+	+	+	+	+	+
	miR-26b-5p	+	+	+	+	+	+	+	+
miR-27	*miR-27a-3p*	+	+	+	+	+	+	+	+
	miR-27b-3p	+	+	+	+	+	+	+	+
miR-29	*miR-29a-3p*	+	+	+	+	+	+	+	+
	miR-29a-5p	+	+	+	+	+	+	-	+
	miR-29b-3p	+	+	+	+	+	+	+	+
	miR-29c-3p	+	+	+	+	+	+	+	+
miR-30	*miR-30a-3p*	+	+	+	+	+	+	+	+
	miR-30a-5p	+	+	+	+	+	+	+	+
	miR-30b-5p	+	+	+	+	+	+	+	+
	miR-30c-2-3p	+	+	+	+	+	+	-	+
	miR-30c-5p	+	+	+	+	+	+	+	+
	miR-30d-3p	+	+	+	+	+	+	+	+
	miR-30d-5p	+	+	+	+	+	+	+	+
	miR-30e-3p	+	+	+	+	+	+	+	+
	miR-30e-5p	+	+	+	+	+	+	+	+
miR-31	*miR-31-5p*	+	+	+	+	+	+	-	+
miR-34	*miR-34a-5p*	+	+	+	+	+	+	+	+
	miR-34b-3p	+	+	+	+	+	+	-	+
	miR-34b-5p	+	+	+	+	+	+	+	+
	miR-34c-5p	+	+	+	+	+	+	+	+
miR-92	*miR-92a-3p*	+	+	+	+	+	+	+	+
	miR-92b-3p	+	+	+	+	+	+	-	+
miR-93	*miR-93-5p*	+	+	+	+	+	+	+	+
miR-96	*miR-96-5p*	+	+	+	-	+	+	+	+
miR-98	*miR-98-5p*	+	+	+	-	+	+	+	+
miR-99	*miR-99a-5p*	+	+	+	-	+	+	+	+
	miR-99b-3p	+	+	+	-	+	+	-	-
	miR-99b-5p	+	+	+	-	+	+	+	+
miR-100	*miR-100-5p*	+	+	+	+	+	+	+	+
miR-101	*miR-101a-3p*	+	+	+	+	+	+	+	+
	miR-101b-3p	+	+	+	+	+	+	+	+
miR-103	*miR-103-3p*	+	+	+	+	+	+	+	+
miR-106	*miR-106b-5p*	+	+	+	+	+	+	+	+
miR-107	*miR-107-3p*	+	+	+	+	+	+	-	+
miR-125	*miR-125a-5p*	+	+	+	+	+	+	+	+
	miR-125b-1-3p	+	+	+	+	+	+	-	-
	miR-125b-2-3p	+	+	+	+	+	+	-	+
	miR-125b-5p	+	+	+	+	+	+	+	+
miR-127	*miR-127-3p*	+	+	+	+	+	+	+	+
miR-128	*miR-128-3p*	+	+	+	+	+	+	-	+
miR-130	*miR-130a-3p*	+	+	+	+	+	+	+	+
miR-132	*miR-132-3p*	+	+	+	+	+	+	-	+
miR-133	*miR-133a-3p*	+	+	+	+	+	+	+	+
miR-135	*miR-135a-5p*	+	+	+	+	+	+	+	+
miR-138	*miR-138-5p*	+	+	+	+	+	+	-	-

miR-139	*miR-139-5p*	+	+	-	+	+	-	-	+
miR-140	*miR-140-3p*	+	+	+	+	+	+	-	+
	miR-140-5p	+	+	+	+	+	+	+	+
miR-141	*miR-141-3p*	+	+	+	+	+	+	+	+
	miR-141-5p	+	+	+	+	+	+	-	+
miR-143	*miR-143-3p*	+	+	+	+	+	+	+	+
miR-144	*miR-144-3p*	+	+	-	+	+	-	-	+
miR-146	*miR-146a-5p*	+	+	+	+	+	+	+	+
	miR-146b-5p	+	+	+	+	+	+	+	+
miR-148	*miR-148a-3p*	+	+	+	+	+	+	+	+
	miR-148a-5p	+	+	+	+	+	+	+	+
	miR-148b-3p	+	+	+	+	+	+	+	+
	miR-148b-5p	+	+	+	+	+	+	-	-
miR-149	*miR-149-5p*	+	+	+	+	+	+	-	+
miR-150	*miR-150-5p*	+	+	+	+	+	+	+	+
miR-151	*miR-151-3p*	+	+	+	+	+	+	+	+
	miR-151-5p	+	+	+	+	+	+	+	+
miR-152	*miR-152-3p*	+	+	+	+	+	+	+	+
	miR-152-5p	+	+	+	+	+	+	-	+
miR-153	*miR-153-3p*	+	+	+	+	+	+	-	-
miR-181	*miR-181a-1-3p*	+	+	+	+	+	+	-	+
	miR-181a-5p	+	+	+	+	+	+	+	+
	miR-181b-5p	+	+	+	+	+	+	+	+
	miR-181c-3p	+	+	+	+	+	+	+	+
	miR-181c-5p	+	+	+	+	+	+	+	+
	miR-181d-5p	+	+	+	+	+	+	+	+
miR-182	*miR-182-5p*	+	+	+	+	+	+	+	+
miR-183	*miR-183-5p*	+	+	+	+	+	+	+	+
miR-184	*miR-184-3p*	+	+	+	+	+	-	+	+
miR-186	*miR-186-5p*	+	+	+	+	+	+	+	+
miR-187	*miR-187-3p*	+	+	+	+	+	+	+	+
miR-191	*miR-191-5p*	+	+	+	+	+	+	+	+
miR-192	*miR-192-5p*	+	+	+	+	+	+	+	+
miR-194	*miR-194-5p*	+	+	+	+	+	+	-	+
miR-196	*miR-196a-5p*	+	+	+	+	+	+	+	+
	miR-196b-5p	+	+	+	+	+	+	+	+
miR-199	*miR-199a-3p*	+	+	+	+	+	+	+	+
	miR-199a-5p	+	+	+	+	+	+	+	+
	miR-199b-3p	+	+	+	+	+	+	+	+
	miR-199b-5p	+	+	+	+	+	+	-	+
miR-200	*miR-200a-3p*	+	+	+	+	+	+	+	+
	miR-200a-5p	+	+	+	+	+	+	+	+
	miR-200b-3p	+	+	+	+	+	+	+	+
	miR-200b-5p	+	+	+	+	+	+	-	+
	miR-200c-3p	+	+	+	+	+	+	+	+
miR-203	*miR-203-3p*	+	+	+	+	+	+	+	+
miR-204	*miR-204-5p*	+	+	+	+	+	+	+	+
miR-205	*miR-205-5p*	+	+	+	+	+	+	+	+
miR-210	*miR-210-3p*	+	+	+	+	+	+	+	+
miR-214	*miR-214-3p*	+	+	+	+	+	+	+	+
miR-218	*miR-218-5p*	+	+	+	+	+	+	+	+
miR-221	*miR-221-3p*	+	+	+	+	+	+	+	+
	miR-221-5p	+	+	+	+	+	+	-	-
miR-222	*miR-222-3p*	+	+	+	+	+	+	+	+
miR-300	*miR-300-3p*	+	+	+	+	+	-	-	+
miR-301	*miR-301a-3p*	+	+	+	+	+	+	+	+
miR-320	*miR-320-3p*	+	+	+	+	+	+	+	+
miR-322	*miR-322-5p*	+	+	+	+	+	+	+	+
miR-	*miR-324-5p*	+	+	+	+	+	+	+	+
miR-326	*miR-326-3p*	+	+	+	+	+	+	-	+
miR-328	*miR-328-3p*	+	+	+	+	+	+	+	+
miR-338	*miR-338-3p*	+	+	+	+	+	+	-	+
miR-339	*miR-339-5p*	+	+	+	+	+	+	-	+
miR-340	*miR-340-5p*	+	+	+	+	+	+	+	+
miR-342	*miR-342-3p*	+	+	+	+	+	+	+	+
miR-350	*miR-350-3p*	+	+	+	-	+	+	-	-
miR-351	*miR-351-5p*	+	+	+	+	+	+	+	+
miR-361	*miR-361-5p*	+	+	+	+	+	+	+	+
miR-362	*miR-362-3p*	+	+	+	+	+	+	-	-
miR-365	*miR-365-3p*	+	+	+	+	+	-	-	+
miR-375	*miR-375-3p*	+	+	+	+	+	+	+	+
miR-379	*miR-379-5p*	+	+	+	+	+	-	-	+
miR-381	*miR-381-3p*	+	+	+	+	+	-	-	+
miR-409	*miR-409-5p*	+	+	+	+	+	-	-	+
miR-410	*miR-410-3p*	+	+	+	+	+	+	+	+
miR-411	*miR-411-5p*	+	+	+	+	+	+	+	+
miR-421	*miR-421-3p*	+	+	+	+	+	+	+	+
miR-423	*miR-423-3p*	+	+	+	+	+	+	+	+
miR-423	*miR-423-5p*	+	+	+	+	+	+	+	+
miR-425	*miR-425-5p*	+	+	+	+	+	+	+	+
miR-429	*miR-429-3p*	+	+	+	+	+	+	+	+

miR-434	*miR-434-3p*	+	+	+	+	+	+	+	+
miR-449	*miR-449a-5p*	+	+	+	+	+	+	-	+
miR-450	*miR-450a-5p*	+	+	+	+	+	+	-	+
miR-455	*miR-455-5p*	+	+	+	+	+	+	-	-
miR-465	*miR-465a-5p*	+	+	+	+	+	-	-	+
	miR-465c-5p	+	+	+	+	+	-	+	+
miR-467	*miR-467a-5p*	+	+	+	+	+	+	+	+
	miR-467d-5p	+	+	+	+	-	+	-	-
miR-470	*miR-470-5p*	+	+	+	+	+	+	+	+
miR-484	*miR-484*	+	+	+	+	+	+	+	+
miR-501	*miR-501-3p*	+	+	+	+	+	+	+	+
miR-532	*miR-532-5p*	+	+	+	+	+	+	+	+
miR-541	*miR-541-5p*	+	+	+	+	+	-	-	+
miR-574	*miR-574-3p*	+	+	+	+	+	+	-	+
miR-582	*miR-582-3p*	+	+	+	+	+	+	-	-
	miR-582-5p	+	+	+	-	+	+	-	-
miR-652	*miR-652-3p*	+	+	+	+	+	+	+	+
miR-669	*miR-669a-5p*	+	+	+	-	-	+	-	-
	miR-669c-5p	+	+	+	+	+	+	-	-
	miR-669p-5p	+	+	+	-	-	+	-	-
miR-671	*miR-671-3p*	+	+	+	+	+	+	-	-
miR-672	*miR-672-5p*	+	+	+	+	+	+	+	+
miR-676	*miR-676-3p*	+	+	+	+	+	+	-	+
miR-741	*miR-741-3p*	+	+	+	+	+	-	+	+
miR-743	*miR-743b-3p*	+	+	+	+	+	-	+	+
miR-744	*miR-744-5p*	+	+	+	+	+	+	+	+
miR-871	*miR-871-3p*	+	+	+	+	+	+	+	+
	miR-871-5p	+	+	+	+	+	-	-	+
miR-872	*miR-872-3p*	+	+	+	+	+	+	+	+
	miR-872-5p	+	+	+	+	+	+	+	+
miR-881	*miR-881-3p*	+	+	+	+	+	-	+	+
miR-1198	*miR-1198-5p*	+	+	+	+	+	+	-	+
miR-1249	*miR-1249-3p*	+	+	+	+	+	+	-	-
miR-1251	*miR-1251-5p*	+	+	+	+	+	+	-	-
miR-1839	*miR-1839-5p*	+	+	+	+	+	+	+	+
miR-1843	*miR-1843b-5p*	+	+	+	+	+	+	-	-
miR-1	*miR-1a-3p*	+	-	+	+	+	-	-	-
miR-7	*let-7c-1-3p*	+	-	+	+	+	-	-	-
	let-7e-3p	+	-	+	+	+	-		-
	let-7f-1-3p	+	-	+	+	+	-		-
	let-7i-3p	+	-	+	+	+	-	-	-
	let-7j	+	-	+	+	+	-	-	-
	miR-7a-1-3p	+	-	+	+	+	-	-	-
miR-10	*miR-10b-3p*	+	-	+	+	+	-	-	-
miR-15	*miR-15b-3p*	+	-	+	+	+	-	-	-
miR-17	*miR-17-3p*	+	-	+	+	-	-	-	-
miR-18	*miR-18a-5p*	+	-	+	+	+	-	-	-
miR-21	*miR-21a-3p*	+	-	+	+	+	-	-	-
	miR-21a-5p	+	-	+	+	+	-	-	-
miR-26	*miR-26a-2-3p*	+	-	+	-	+	-	-	-
	miR-26b-3p	+	-	+	+	+	-	-	-
miR-27	*miR-27a-5p*	+	-	+	+	+	-	-	-
	miR-27b-5p	+	-	+	+	+	-	-	-
miR-28	*miR-28a-3p*	+	-	+	+	+	-	-	-
	miR-28a-5p	+	-	+	+	+	-	-	-
miR-30	*miR-30b-3p*	+	-	+	+	+	-	-	-
	miR-30c-1-3p	+	-	+	+	+	-	-	-
miR-31	*miR-31-3p*	+	-	+	-	-	-	-	-
miR-32	*miR-32-3p*	+	-	+	-	-	-	-	-
	miR-32-5p	+	-	+	-	-	-	-	-
miR-33	*miR-33-3p*	+	-	+	-	-	-	-	-
miR-34	*miR-34c-3p*	+	-	+	+	+	-	-	-
miR-92	*miR-92a-1-5p*	+	-	+	+	+	-	-	-
miR-96	*miR-96-3p*	+	-	+	-	-	-	-	-
miR-98	*miR-98-3p*	+	-	+	-	+	-	-	-
miR-99	*miR-99a-3p*	+	-	+	-	+	-	-	-
miR-101	*miR-101a-5p*	+	-	+	-	+	-	-	-
miR-106	*miR-106b-3p*	+	-	+	-	+	-	-	-
miR-124	*miR-124-3p*	+	-	-	-	+	-	-	-
miR-125	*miR-125a-3p*	+	-	+	+	+	-	-	-
miR-126	*miR-126a-3p*	+	-	+	+	+	-	-	-
	miR-126a-5p	+	-	+	+	+	-	-	-
miR-127	*miR-127-5p*	+	-	-	-	+	-	-	-
miR-130	*miR-130b-3p*	+	-	+	+	+	-	-	-
	miR-130b-5p	+	-	+	+	+	-	-	-
miR-133	*miR-133b-3p*	+	-	-	-	+	-	-	-
miR-134	*miR-134-5p*	+	-	-	-	+	-	-	-
miR-135	*miR-135a-2-3p*	+	-	+	-	-	-	-	-
	miR-135b-5p	+	-	+	+	-	-	-	-
miR-136	*miR-136-3p*	+	-	+	+	+	-	-	-
	miR-136-5p	+	-	+	+	+	-	-	-

miR-137	*miR-137-3p*	+	-	-	-	+	-	-	-
miR-142	*miR-142a-3p*	+	-	+	+	+	-	-	-
	miR-142a-5p	+	-	+	+	+	-	-	-
miR-143	*miR-143-5p*	+	-	-	-	+	-	-	-
miR-144	*miR-144-5p*	+	-	-	+	+	-	-	-
miR-145	*miR-145a-3p*	+	-	+	+	+	-	-	-
	miR-145a-5p	+	-	+	+	+	-	-	-
miR-154	*miR-154-5p*	+	-	-	+	+	-	-	-
miR-155	*miR-155-5p*	+	-	-	-	+	-	-	-
miR-185	*miR-185-5p*	+	-	+	+	+	-	-	-
miR-188	*miR-188-3p*	+	-	-	-	+	-	-	-
miR-190	*miR-190a-3p*	+	-	-	+	-	-	-	-
	miR-190a-5p	+	-	+	+	+	-	-	-
miR-191	*miR-191-3p*	+	-	+	-	-	-	-	-
miR-193	*miR-193a-3p*	+	-	+	+	+	-	-	-
	miR-193b-3p	+	-	+	+	+	-	-	-
miR-195	*miR-195a-3p*	+	-	+	+	+	-	-	-
	miR-195a-5p	+	-	+	+	+	-	-	-
miR-196	*miR-196a-2-3p*	+	-	-	+	+	-	-	-
	miR-196b-3p	+	-	-	-	+	-	-	-
miR-200	*miR-200c-5p*	+	-	+	+	+	-	-	-
miR-203	*miR-203-5p*	+	-	+	-	+	-	-	-
miR-205	*miR-205-3p*	+	-	+	+	+	-	-	-
miR-206	*miR-206-3p*	+	-	-	-	+	-	-	-
miR-208	*miR-208b-3p*	+	-	-	-	+	-	-	-
miR-210	*miR-210-5p*	+	-	+	+	+	-	-	-
miR-211	*miR-211-5p*	+	-	-	+	-	-	-	-
miR-212	*miR-212-5p*	+	-	-	-	+	-	-	-
miR-214	*miR-214-5p*	+	-	+	+	+	-	-	-
miR-222	*miR-222-5p*	+	-	+	-	-	-	-	-
miR-223	*miR-223-3p*	+	-	+	+	+	-	-	-
miR-296	*miR-296-5p*	+	-	+	+	+	-	-	-
miR-298	*miR-298-5p*	+	-	+	+	+	-	-	-
miR-299	*miR-299a-3p*	+	-	-	-	+	-	-	-
	miR-299a-5p	+	-	-	-	+	-	-	-
miR-301	*miR-301b-3p*	+	-	+	-	+	-	-	-
miR-322	*miR-322-3p*	+	-	+	+	+	-	-	-
miR-324	*miR-324-3p*	+	-	-	+	-	-	-	-
miR-329	*miR-329-3p*	+	-	-	-	+	-	-	-
	miR-329-5p	+	-	+	-	+	-	-	-
miR-330	*miR-330-5p*	+	-	+	+	+	-	-	-
miR-331	*miR-331-3p*	+	-	+	+	+	-	-	-
miR-335	*miR-335-3p*	+	-	+	+	+	-	-	-
	miR-335-5p	+	-	+	+	+	-	-	-
miR-337	*miR-337-5p*	+	-	+	+	+	-	-	-
miR-339	*miR-339-3p*	+	-	+	+	+	-	-	-
miR-340	*miR-340-3p*	+	-	+	+	+	-	-	-
miR-341	*miR-341-3p*	+	-	+	+	+	-	-	-
miR-345	*miR-345-5p*	+	-	+	+	-	-	-	-
miR-361	*miR-361-3p*	+	-	+	-	-	-	-	-
miR-362	*miR-362-5p*	+	-	+	-	-	-	-	-
miR-369	*miR-369-3p*	+	-	-	+	+	-	-	-
miR-374	*miR-374b-5p*	+	-	+	+	+	-	-	-
miR-376	*miR-376a-3p*	+	-	-	-	+	-	-	-
	miR-376a-5p	+	-	-	-	+	-	-	-
	miR-376b-3p	+	-	+	+	+	-	-	-
	miR-376c-3p	+	-	+	-	+	-	-	-
miR-378	*miR-378a-3p*	+	-	+	+	+	-	-	-
	miR-378a-5p	+	-	+	+	+	-	-	-
	miR-378b	+	-	-	-	+	-	-	-
	miR-378c	+	-	+	+	+	-	-	-
	miR-378d	+	-	+	+	+	-	-	-
miR-379	*miR-379-3p*	+	-	-	-	+	-	-	-
miR-382	*miR-382-5p*	+	-	+	+	+	-	-	-
miR-409	*miR-409-3p*	+	-	+	+	+	-	-	-
miR-411	*miR-411-3p*	+	-	-	-	+	-	-	-
miR-431	*miR-431-5p*	+	-	-	-	+	-	-	-
miR-434	*miR-434-5p*	+	-	+	+	+	-	-	-
miR-450	*miR-450b-5p*	+	-	+	-	-	-	-	-
miR-451	*miR-451a*	+	-	+	+	+	-	-	-
miR-463	*miR-463-5p*	+	-	-	-	+	-	-	-
miR-465	*miR-465a-3p*	+	-	+	+	+	-	-	-
	miR-465b-3p	+	-	+	+	+	-	-	-
	miR-465b-5p	+	-	-	+	+	-	-	-
	miR-465c-3p	+	-	+	+	+	-	-	-
miR-466	*miR-466b-3p*	+	-	+	-	-	-	-	-
	miR-466c-3p	+	-	+	-	-	-	-	-
	miR-466g	+	-	+	-	-	-	-	-
	miR-467b-5p	+	-	+	+	-	-	-	-
miR-467	*miR-467c-5p*	+	-	+	+	+	-	-	-
	miR-467d-3p	+	-	+	-	-	-	-	-

	miR-467e-5p	+	-	+	+	+	-	-	-
	miR-470-3p	+	-	-	+	+	-	-	-
miR-470	*miR-471-3p*	+	-	-	+	+	-	-	-
miR-471	*miR-485-5p*	+	-	-	-	+	-	-	-
miR-485	*miR-486a-3p*	+	-	-	-	+	-	-	-
miR-486	*miR-486a-5p*	+	-	+	+	+	-	-	-
	miR-486b-5p	+	-	+	+	+	-	-	-
	miR-487b-3p	+	-	-	-	+	-	-	-
miR-487	*miR-497a-5p*	+	-	+	+	+	-	-	-
miR-497	*miR-499-5p*	+	-	-	-	+	-	-	-
miR-499	*miR-500-3p*	+	-	+	-	+	-	-	-
miR-500	*miR-511-3p*	+	-	+	+	+	-	-	-
miR-511	*miR-532-3p*	+	-	+	+	+	-	-	-
miR-532	*miR-574-5p*	+	-	+	+	+	-	-	-
miR-574	*miR-598-3p*	+	-	+	+	-	-	-	-
miR-598	*miR-615-3p*	+	-	+	+	+	-	-	-
miR-615	*miR-669a-3p*	+	-	+	-	+	-	-	-
miR-669	*miR-669f-5p*	+	-	+	-	-	-	-	-
	miR-669l-5p	+	-	+	-	-	-	-	-
	miR-669o-3p	+	-	+	-	+	-	-	-
	miR-669o-5p	+	-	+	+	+	-	-	-
	miR-672-3p	+	-	+	+	-	-	-	-
miR-672	*miR-674-3p*	+	-	+	-	+	-	-	-
miR-674	*miR-676-5p*	+	-	+	+	+	-	-	-
miR-676	*miR-677-5p*	+	-	-	+	+	-	-	-
miR-677	*miR-708-3p*	+	-	+	+	+	-	-	-
miR-708	*miR-708-5p*	+	-	-	+	+	-	-	-
	miR-741-5p	+	-	-	-	+	-	-	-
miR-741	*miR-743a-3p*	+	-	+	+	+	-	-	-
miR-743	*miR-743a-5p*	+	-	-	-	+	-	-	-
	miR-744-3p	+	-	+	+	+	-	-	-
miR-744	*miR-874-3p*	+	-	+	+	+	-	-	-
miR-874	*miR-874-5p*	+	-	+	+	+	-	-	-
	miR-878-5p	+	-	-	+	+	-	-	-
miR-878	*miR-880-3p*	+	-	-	+	+	-	-	-
miR-880	*miR-883a-3p*	+	-	-	+	+	-	-	-
miR-883	*miR-1247-3p*	+	-	+	+	+	-	-	-
miR-1247	*miR-1247-5p*	+	-	-	+	+	-	-	-
	miR-1839-3p	+	-	+	-	-	-	-	-
miR-1839	*miR-1843a-5p*	+	-	+	+	+	-	-	-
miR-1843	*miR-1981-3p*	+	-	+	+	+	-	-	-
miR-1981	*miR-1981-5p*	+	-	+	+	+	-	-	-
	miR-3068-3p	+	-	+	+	+	-	-	-
miR-3068	*miR-3068-5p*	+	-	+	-	-	-	-	-
	miR-3082-3p	+	-	-	+	-	-	-	-
miR-3082	*miR-3475-3p*	+	-	-	+	-	-	-	-
miR-3475	*miR-3535*	+	-	+	-	-	-	-	-
miR-3535	*miR-8114*	+	-	+	+	+	-	-	-
miR-8114	*miR-466p-3p*	+	-	+	-	-	-	-	-
miR-21	*miR-21-3p*	-	+	-	-	-	+	-	+
	miR-21-5p	-	+	-	-	-	+	+	+
miR-28	*miR-28-3p*	-	+	-	-	-	+	+	+
	miR-28-5p	-	+	-	-	-	+	+	+
miR-126	*miR-126-3p*	-	+	-	-	-	+	+	+
	miR-126-5p	-	+	-	-	-	+	+	+
miR-129	*miR-129-2-3p*	-	+	-	-	-	-	-	+
miR-142	*miR-142-5p*	-	+	-	-	-	+	+	+
miR-145	*miR-145-3p*	-	+	-	-	-	+	+	+
	miR-145-5p	-	+	-	-	-	+	+	+
miR-190	*miR-190-5p*	-	+	-	-	-	-	+	+
miR-193	*miR-193-3p*	-	+	-	-	-	+	+	+
miR-195	*miR-195-5p*	-	+	-	-	-	+	+	+
miR-374	*miR-374-5p*	-	+	-	-	-	+	+	+
miR-378	*miR-378-3p*	-	+	-	-	-	+	+	+
	miR-378-5p	-	+	-	-	-	+	-	-
miR-451	*miR-451*	-	+	-	-	-	+	-	+
miR-486	*miR-486-5p*	-	+	-	-	-	+	+	+
miR-497	*miR-497-5p*	-	+	-	-	-	+	+	+
miR-720	*miR-720*	-	+	-	-	-	+	-	-
miR-1843	*miR-1843-5p*	-	+	-	-	-	+	+	+
miR-3096	*miR-3096-5p*	-	+	-	-	-	+	-	+
miR-3107	*miR-3107-5p*	-	+	-	-	-	+	+	+

Total Whole Tissue
370

Total Epithelial Cells
218

Total
393

195 miRNAs common to both whole tissue and epithelial cells
175 miRNAs unique to whole tissue
23 miRNAs unique to epithelial cells
