# Supplementary material for: Next Generation Sequencing Analysis Reveals Segmental Patterns of microRNA Expression in Mouse Epididymal Epithelial Cells
Source: PLoS One. 2015 Aug 13;10(8):e0135605. doi: 10.1371/journal.pone.0135605 (PMC4535982; doi:10.1371/journal.pone.0135605)
Supplement: S2 Table — (PDF) [file pone.0135605.s005.pdf]

S2 Table. Relative expression levels of miRNAs identified by deep sequencing within mouse epididymal epithelial cells

								False Discovery Rate (FDR)		
**MicroRNA Family**	**GeneName**	**Caput 1**	**Caput 2**	**Corpus 1**	**Corpus 2**	**Cauda 1**	**Cauda 2**	**Caput to Corpus**	**Corpus to Cauda**	**Caput to Cauda**
let-7	*let-7a-1-3p*	43.001	46.001	6.501	6.001	23.501	24.501	0.116844317	0.467224731	0.352272471
	let-7a-5p	3240.501	3920.001	1010.501	1437.501	1804.001	2318.001	0.75434466	0.291524728	0.076667217
	let-7b-3p	43.001	31.001	2.001	5.001	12.001	20.001	0.031936796	0.328101241	0.133846455
	let-7b-5p	1661.501	1887.501	372.501	403.501	466.501	655.501	0.067599277	0.200934175	0.000863351
	let-7c-2-3p	43.001	46.001	6.501	6.001	23.501	24.501	0.116844317	0.467224731	0.352272471
	let-7c-5p	13371.001	15785.501	2712.001	3744.001	3814.501	5136.501	0.014705736	0.044880172	3.16156E-05
	let-7d-3p	135.001	166.001	30.001	45.001	119.001	111.001	0.329753301	0.482995553	0.875182121
	let-7d-5p	372.001	411.001	169.001	251.001	311.001	352.001	0.217104047	0.349259749	0.858471933
	let-7e-5p	514.001	588.001	123.001	168.001	150.001	234.001	0.223472254	0.102649063	0.001634182
	let-7f-5p	9065.001	9612.001	3286.001	3944.001	5374.001	6899.001	0.814852018	0.291524728	0.285319522
	let-7g-5p	2201.501	2618.001	920.001	1220.001	1567.501	1482.501	0.490338938	0.156711078	0.319853884
	let-7i-5p	2243.501	2219.001	1082.001	1318.001	1572.501	2030.501	0.101167372	0.143211631	0.994653333
miR-9	*miR-9-3p*	4.001	17.001	0.001	0.001	0.001	0.001	0.043333249	0.534103566	0.004049515
	miR-9-5p	124.001	192.001	1.001	4.001	3.001	7.001	0.002741393	0.917314742	0.000437087
miR-10	*miR-10a-3p*	18.001	28.001	2.001	2.001	1.001	8.001	0.123216848	0.771785412	0.025663191
	miR-10a-5p	20818.001	26151.001	5636.501	8369.001	11100.501	12992.501	0.217104047	0.237111924	0.005888885
	miR-10b-5p	23176.001	30357.001	6584.501	11109.001	11525.501	13759.501	0.508981727	0.03820591	0.00186608
miR-15	*miR-15a-5p*	44.001	48.001	10.001	11.001	31.001	47.001	0.355129386	0.382174315	0.907023921
	miR-15b-5p	73.001	95.001	17.001	20.001	65.001	86.001	0.234115896	0.205394928	0.746116529
miR-16	*miR-16-5p*	2106.001	2410.001	686.001	913.001	2749.001	2958.001	0.874801171	0.043897771	0.020831139
miR-17	*miR-17-5p*	89.001	105.001	38.001	42.001	87.001	81.001	0.749343992	0.880709989	0.831770684
miR-19	*miR-19a-3p*	22.001	38.501	8.001	6.001	31.001	17.001	0.580132729	0.696247668	0.907023921
	miR-19b-3p	188.001	189.501	63.001	59.001	143.001	169.001	0.744087253	0.76034854	0.919532
miR-20	*miR-20a-5p*	123.001	127.001	52.001	66.001	170.001	165.001	0.489146405	0.543968619	0.08873631
miR-21	*miR-21-3p*	32.001	16.001	3.001	13.001	4.001	21.001	0.850570909	0.698757977	0.376800691
	miR-21-5p	11513.001	12981.001	3046.001	3903.001	6178.001	7136.001	0.228338622	0.603968431	0.039557738
miR-22	*miR-22-3p*	25980.001	32993.001	4502.001	7240.001	13511.001	18425.001	0.001229604	0.268644472	0.009983922
	miR-22-5p	23.001	27.001	2.001	3.001	10.001	14.001	0.064882036	0.387788766	0.279152478
miR-23	*miR-23a-3p*	382.501	387.001	91.001	106.001	309.001	523.001	0.207395911	0.039505793	0.230223282
	miR-23b-3p	369.501	323.001	115.001	124.001	256.001	353.001	0.867689567	0.744648597	0.746116529
miR-24	*miR-24-2-5p*	58.001	58.001	15.001	29.001	54.001	83.001	0.960809654	0.467224731	0.280561835
	miR-24-3p	127.001	133.001	36.001	46.001	66.001	103.001	0.740101737	0.868303619	0.473432392
miR-25	*miR-25-3p*	655.001	784.001	261.001	334.001	744.001	975.001	0.682545537	0.35414912	0.080192318
miR-26	*miR-26a-5p*	17006.001	20088.001	5622.001	9091.001	13815.001	19297.001	0.749420353	0.872504638	0.521928702
	miR-26b-5p	431.001	417.001	228.001	224.001	530.001	654.001	0.233134598	0.677270278	0.049133431
miR-27	*miR-27a-3p*	373.501	373.001	95.501	99.001	303.501	672.501	0.284579822	0.031020189	0.098098663
	miR-27b-3p	7990.501	8933.001	2069.501	2702.001	5876.501	10202.501	0.178098817	0.057493605	0.394304603
miR-28	*miR-28-3p*	38.001	51.001	16.001	20.001	28.001	43.001	0.793663415	0.849637459	0.967602756
	miR-28-5p	38.001	23.001	14.001	13.001	26.001	27.001	0.648661003	0.855935899	0.807038844
miR-29	*miR-29a-3p*	1387.001	1806.001	417.001	516.001	793.001	1137.001	0.368266049	0.853412942	0.177720915
	miR-29a-5p	9.001	15.001	5.001	6.001	19.001	43.001	0.718578284	0.287156641	0.039557738
	miR-29b-3p	237.001	224.001	76.001	78.001	96.001	149.001	0.840166717	0.451837323	0.190248349
	miR-29c-3p	204.001	248.001	41.001	59.001	87.001	110.001	0.148635876	0.849637459	0.039839009
miR-30	*miR-30a-3p*	312.001	334.001	120.001	136.001	293.001	577.001	0.793663415	0.234663438	0.055456689
	miR-30a-5p	1948.001	1795.001	988.001	1034.001	1875.001	2821.001	0.079794323	0.876668333	0.032725731
	miR-30b-5p	1603.001	1729.001	621.001	636.001	728.001	1269.001	0.89568274	0.188188621	0.117853881
	miR-30c-2-3p	35.001	38.001	8.001	8.001	18.001	22.001	0.297992636	0.872504638	0.317169798
	miR-30c-5p	1079.001	1016.001	355.001	345.001	682.001	1349.001	0.757003491	0.45889658	0.530031392
	miR-30d-3p	37.001	56.001	11.001	14.001	19.001	36.001	0.619438399	0.913982378	0.412092847
	miR-30d-5p	751.001	755.001	290.001	466.001	794.001	801.001	0.233134598	0.913982378	0.207770947
	miR-30e-3p	204.001	223.001	58.001	71.001	112.001	237.001	0.624772727	0.76034854	0.905007683
	miR-30e-5p	247.001	193.001	123.001	125.001	197.001	265.001	0.142334409	0.641649042	0.319853884
miR-31	*miR-31-5p*	78.001	80.001	5.001	5.001	18.001	12.001	0.017487684	0.853412942	0.006260488
miR-34	*miR-34a-5p*	45.001	58.001	24.001	28.001	32.001	42.001	0.489146405	0.417908191	0.807038844
	miR-34b-3p	15.001	25.001	8.001	10.001	79.001	31.001	0.764626318	0.296519788	0.063902055
	miR-34b-5p	59.501	58.001	7.501	14.501	77.001	33.001	0.29355697	0.349259749	0.936464789
	miR-34c-5p	1077.501	1252.001	150.501	221.501	902.001	442.001	0.112286404	0.494027221	0.352272471
miR-92	*miR-92a-3p*	122.001	163.001	102.001	219.001	212.001	244.001	0.003277264	0.291524728	0.02330421
	miR-92b-3p	42.001	45.001	5.001	7.001	13.001	42.001	0.282773913	0.596712943	0.563568512
miR-93	*miR-93-5p*	235.001	267.001	80.001	106.001	241.001	231.001	0.960809654	0.750564645	0.558761313
miR-96	*miR-96-5p*	142.001	178.001	43.001	44.001	84.001	86.001	0.385199839	0.812232041	0.143176053
miR-98	*miR-98-5p*	284.001	305.001	92.001	144.001	106.001	138.001	0.831165866	0.044880172	0.026817099
miR-99	*miR-99a-5p*	379.001	410.001	232.001	234.001	440.001	717.001	0.080943661	0.805598074	0.018864892
	miR-99b-3p	16.001	20.001	1.001	8.001	5.001	6.001	0.355129386	0.773059572	0.106418704
	miR-99b-5p	5963.001	6941.001	1551.001	2603.001	2332.001	3835.001	0.515942896	0.126967061	0.009611073
miR-100	*miR-100-5p*	1511.001	1380.001	370.001	554.001	990.001	2000.001	0.699451604	0.349259749	0.447825343
miR-101	*miR-101a-3p*	351.001	351.001	183.001	235.001	343.001	430.001	0.171302727	0.708353875	0.319853884
	miR-101b-3p	38.001	38.001	19.001	25.001	36.001	52.001	0.233134598	0.853412942	0.299698833
miR-103	*miR-103-3p*	898.001	1011.501	339.001	432.001	524.501	813.501	0.718578284	0.349259749	0.438398016
miR-106	*miR-106b-5p*	38.001	30.001	29.001	46.001	52.001	46.001	0.011420079	0.242723084	0.112671946
miR-107	*miR-107-3p*	48.001	49.501	8.001	10.001	14.501	22.501	0.154532828	0.868303619	0.04626529
miR-125	*miR-125a-5p*	491.001	735.001	90.001	172.001	326.001	380.001	0.068851655	0.518890568	0.210350374
	miR-125b-1-3p	19.001	14.001	3.001	10.001	4.001	7.001	0.960809654	0.279487659	0.131632143
	miR-125b-2-3p	18.001	25.001	5.001	12.001	11.001	11.001	0.915488987	0.488462109	0.389411401
	miR-125b-5p	1827.001	2064.001	614.001	800.001	1269.001	2228.001	0.960809654	0.790625652	0.715605865
miR-126	*miR-126-3p*	718.001	1081.001	103.001	155.001	861.001	1088.001	0.006239608	0.001012289	0.204266224
	miR-126-5p	7202.001	10409.001	1179.001	1610.001	8904.001	14131.001	0.001229604	1.53834E-05	0.010084777
miR-127	*miR-127-3p*	404.001	417.001	208.001	281.001	744.001	1532.001	0.082560614	0.021443417	5.73932E-05
miR-128	*miR-128-3p*	37.001	46.001	8.001	10.001	30.001	19.001	0.382956561	0.868303619	0.420667584
miR-129	*miR-129-2-3p*	4.001	8.001	1.001	2.001	6.001	10.001	0.847748992	0.426953223	0.394304603
miR-130	*miR-130a-3p*	205.001	179.001	84.001	104.001	119.001	254.001	0.458235045	0.778326759	0.715605865
miR-132	*miR-132-3p*	36.001	37.001	6.001	11.001	11.001	32.001	0.454039195	0.959573985	0.378505542
miR-133	*miR-133a-3p*	106.001	111.001	53.001	68.501	53.501	118.501	0.374474551	0.380708399	0.875182121
miR-135	*miR-135a-5p*	655.001	577.001	184.001	159.001	221.001	291.001	0.355129386	0.287156641	0.015342282
miR-138	*miR-138-5p*	40.001	64.001	3.001	8.001	5.001	6.001	0.024658779	0.319550008	0.000407164
miR-139	*miR-139-5p*	5.001	9.001	0.001	0.001	16.001	8.001	0.060059467	0.01400386	0.31368552
miR-140	*miR-140-3p*	13.001	12.001	2.001	2.001	11.001	13.001	0.29390583	0.261250415	0.728767275
	miR-140-5p	40.001	35.001	12.001	11.001	38.001	99.001	0.808599866	0.126967061	0.070010069
miR-141	*miR-141-3p*	263.001	351.501	190.501	238.501	188.501	215.001	0.023338489	0.008530076	0.423173696
	miR-141-5p	25.001	23.001	4.001	13.001	14.001	14.001	0.874854122	0.834555908	0.531305904
miR-142	*miR-142-5p*	103.001	193.001	37.001	63.001	189.001	254.001	0.907463338	0.143211631	0.06612475
miR-143	*miR-143-3p*	57905.001	45045.001	19489.001	25580.001	32077.001	85134.001	0.62480896	0.872728537	0.418441451
miR-144	*miR-144-3p*	2.001	4.001	1.001	0.001	7.001	16.001	0.759138058	0.086305784	0.077626575
miR-145	*miR-145-3p*	34.001	36.001	5.001	18.001	25.001	41.001	0.726765782	0.593179643	0.753828477
	miR-145-5p	2801.001	1359.001	1071.001	557.001	1021.001	5267.001	0.898487097	0.636098135	0.402272257
miR-146	*miR-146a-5p*	150.501	198.001	77.001	72.001	169.001	246.001	0.726765782	0.677270278	0.284041017
	miR-146b-5p	312.501	242.001	9.001	21.001	24.001	26.001	0.00037469	0.76034854	3.16156E-05
miR-148	*miR-148a-3p*	33789.001	40210.001	7251.001	12432.001	8979.001	9368.001	0.127048138	0.002429077	3.16156E-05
	miR-148a-5p	201.001	240.001	10.001	20.001	37.001	37.001	0.000309671	0.853412942	5.31486E-05
	miR-148b-3p	165.001	185.001	56.001	70.001	118.001	150.001	0.960809654	0.899848412	0.841959507

	miR-148b-5p	11.001	16.001	2.001	2.001	9.001	10.001	0.29355697	0.467224731	0.832943518
miR-149	*miR-149-5p*	59.001	61.001	3.001	14.001	6.001	31.001	0.131578713	0.912754066	0.04626529
miR-150	*miR-150-5p*	38.001	69.001	7.001	17.001	86.001	149.001	0.29355697	0.00366284	0.004256332
miR-151	*miR-151-3p*	509.001	639.001	121.001	198.001	311.001	518.001	0.253481614	0.605557613	0.562967224
	miR-151-5p	544.001	772.001	134.001	285.001	399.001	471.001	0.558615943	0.992549801	0.428401486
miR-152	*miR-152-3p*	65.001	58.001	15.001	34.001	53.001	71.001	0.922547723	0.794952501	0.548944347
	miR-152-5p	27.001	23.001	7.001	7.001	15.001	23.001	0.756802832	0.856017331	0.907023921
miR-153	*miR-153-3p*	14.001	25.001	6.001	1.001	7.001	3.001	0.29355697	0.841437433	0.106203318
miR-181	*miR-181a-1-3p*	12.001	28.001	6.001	8.001	15.001	29.001	0.916370198	0.716717296	0.431302402
	miR-181a-5p	3086.001	3968.001	875.001	1400.001	2384.001	4529.001	0.563622302	0.236471244	0.393360055
	miR-181b-5p	77.001	132.001	32.001	62.501	83.001	107.001	0.640586485	0.880709989	0.70566562
	miR-181c-3p	125.001	123.001	36.001	41.001	56.001	73.001	0.7049038	0.518890568	0.148227216
	miR-181c-5p	2361.001	3010.001	1108.001	1800.001	2480.001	2945.001	0.104979534	0.600239875	0.272083197
	miR-181d-5p	229.001	263.001	124.001	191.501	302.001	342.001	0.045758815	0.867705265	0.045562035
miR-182	*miR-182-5p*	2165.001	2716.001	348.001	534.001	610.001	629.001	0.011420079	0.173973906	8.70254E-05
miR-183	*miR-183-5p*	835.001	913.001	215.001	260.001	315.001	540.001	0.217104047	0.438703337	0.016438454
miR-184	*miR-184-3p*	5.001	4.001	17.001	34.001	27.001	6.001	0.003875638	0.0494697	0.148227216
miR-186	*miR-186-5p*	174.001	220.001	137.001	195.001	195.001	176.001	0.008357307	0.039505793	0.563568512
miR-187	*miR-187-3p*	39.001	50.001	9.001	16.001	12.001	22.001	0.619385307	0.380708399	0.061087467
miR-190	*miR-190-5p*	6.001	6.001	39.001	29.001	31.001	25.001	0.000362152	0.068922735	0.007055401
miR-191	*miR-191-5p*	18787.001	22938.001	2629.001	4725.001	5959.001	6004.001	0.001229604	0.291524728	3.16156E-05
miR-192	*miR-192-5p*	802.001	1131.001	153.001	231.001	454.001	713.001	0.178098817	0.549859442	0.420855708
miR-193	*miR-193-3p*	25.001	21.001	7.001	13.001	10.001	26.001	0.770558799	0.750564645	0.863717722
miR-194	*miR-194-5p*	37.001	51.001	9.001	8.001	20.001	32.001	0.382956561	0.784073127	0.563568512
miR-195	*miR-195-5p*	478.001	379.001	135.001	155.001	157.001	245.001	0.845082443	0.177086088	0.046346215
miR-196	*miR-196a-5p*	10.001	12.001	111.001	113.001	118.001	185.001	1.80198E-05	0.275097663	3.54062E-05
	miR-196b-5p	11.001	29.001	119.001	148.001	424.001	1072.001	4.93534E-05	0.044880172	1.02274E-06
miR-199	*miR-199a-3p*	400.501	380.501	188.001	212.501	444.501	852.501	0.249252552	0.296519788	0.009983922
	miR-199a-5p	149.001	138.501	40.001	63.001	62.001	116.001	0.94792566	0.543968619	0.354758396
	miR-199b-3p	400.501	380.501	188.001	212.501	444.501	852.501	0.249252552	0.296519788	0.009983922
	miR-199b-5p	21.001	17.501	4.001	5.001	16.001	45.001	0.619385307	0.171987866	0.190248349
miR-200	*miR-200a-3p*	1166.001	1318.501	467.501	615.501	1084.501	963.001	0.640586485	0.750564645	0.936464789
	miR-200a-5p	40.001	53.001	8.001	24.001	21.001	22.001	0.784301294	0.494027221	0.177720915
	miR-200b-3p	918.001	1120.001	405.001	473.001	1468.001	1358.001	0.648661003	0.296519788	0.057117227
	miR-200b-5p	26.001	20.001	7.001	4.001	13.001	15.001	0.60342456	0.870169895	0.649131957
	miR-200c-3p	1200.001	1416.001	455.001	579.001	590.001	498.001	0.795926833	0.022396009	0.010084777
miR-203	*miR-203-3p*	56.001	93.001	13.001	17.001	50.001	61.001	0.231816378	0.349259749	0.875182121
miR-204	*miR-204-5p*	7611.001	8467.501	533.001	878.001	163.001	169.001	1.39946E-05	1.14382E-06	1.26E-10
miR-205	*miR-205-5p*	3355.001	3594.001	2185.001	2568.001	4708.001	5434.001	0.031363446	0.907561538	0.018864892
miR-210	*miR-210-3p*	179.001	213.001	115.001	147.001	77.001	114.001	0.031936796	0.001012289	0.050786432
miR-214	*miR-214-3p*	40.001	40.001	13.001	10.001	10.001	20.001	0.740101737	0.490425066	0.150960998
miR-218	*miR-218-5p*	37.001	36.001	11.001	14.001	16.001	24.001	0.935389018	0.543968619	0.334164459
miR-221	*miR-221-3p*	341.001	366.001	131.001	146.001	306.001	407.001	0.814810132	0.677270278	0.351832624
	miR-221-5p	16.001	15.001	4.001	1.001	6.001	5.001	0.245986661	0.957134072	0.193119216
miR-222	*miR-222-3p*	33.001	33.001	71.001	68.001	183.001	150.001	0.000284278	0.868303619	3.71291E-05
miR-300	*miR-300-3p*	4.001	3.001	4.001	3.001	13.001	16.001	0.131578713	0.417908191	0.006800246
miR-301	*miR-301a-3p*	298.001	281.001	126.001	173.001	174.001	269.001	0.233134598	0.234257603	0.824591674
miR-320	*miR-320-3p*	95.001	100.001	39.001	63.001	79.001	61.001	0.355129386	0.287156641	0.715605865
miR-322	*miR-322-5p*	134.001	214.001	25.001	49.001	159.001	206.001	0.116379637	0.034149076	0.307442061
miR-324	*miR-324-5p*	44.001	40.001	26.001	17.001	55.001	68.001	0.506059627	0.588389043	0.101152817
miR-326	*miR-326-3p*	19.001	11.001	6.001	3.001	10.001	14.001	0.852238981	0.856017331	0.934538054
miR-328	*miR-328-3p*	51.001	50.001	12.001	22.001	32.001	54.001	0.852238981	0.855935899	0.912053176
	miR-338-3p	13.001	22.001	4.001	1.001	17.001	24.001	0.217104047	0.107232451	0.432722937
miR-339	*miR-339-5p*	10.001	15.001	1.001	3.001	5.001	15.001	0.365431406	0.500446429	0.907023921
miR-340	*miR-340-5p*	865.001	918.001	147.001	185.001	252.001	354.001	0.023338489	0.593179643	0.001645886
miR-342	*miR-342-3p*	50.001	43.001	21.001	24.001	54.001	77.001	0.508981727	0.602516409	0.108312421
miR-350	*miR-350-3p*	11.001	12.001	2.001	2.001	5.001	4.001	0.379906351	0.880709989	0.229819321
miR-351	*miR-351-5p*	31.001	57.001	8.001	15.001	44.001	37.001	0.619438399	0.467224731	0.735923361
miR-361	*miR-361-5p*	53.001	68.001	15.001	16.001	20.001	65.001	0.508981727	0.880709989	0.501584088
miR-362	*miR-362-3p*	15.001	11.001	0.001	2.001	3.001	9.001	0.088110864	0.417908191	0.393360055
miR-365	*miR-365-3p*	9.001	9.001	1.001	0.001	6.001	22.001	0.103763137	0.039505793	0.402272257
miR-374	*miR-374-5p*	32.001	31.001	11.001	10.001	11.001	22.001	0.89568274	0.543968619	0.317169798
miR-375	*miR-375-3p*	1089.001	1356.001	160.001	253.001	112.001	31.001	0.101167372	0.002699887	3.16156E-05
miR-378	*miR-378-3p*	264.001	301.001	94.001	108.001	137.001	226.001	0.960481299	0.549859442	0.38085667
	miR-378-5p	16.001	14.001	2.001	2.001	7.001	8.001	0.217104047	0.694257026	0.393360055
miR-379	*miR-379-5p*	5.001	6.001	1.001	2.001	16.001	25.001	0.871395019	0.022396009	0.006380521
miR-381	*miR-381-3p*	7.001	8.001	2.001	5.001	17.001	33.001	0.722670773	0.083014493	0.007055401
miR-409	*miR-409-5p*	3.001	0.001	3.001	2.001	8.001	22.001	0.069713203	0.414897013	0.003134955
miR-410	*miR-410-3p*	26.001	17.001	11.001	17.001	69.001	133.001	0.249252552	0.034149076	0.000276796
miR-411	*miR-411-5p*	77.001	63.001	20.001	41.001	130.001	321.001	0.756802832	0.01400386	0.000606487
miR-421	*miR-421-3p*	28.001	34.001	15.001	33.001	31.001	49.001	0.091782181	0.609075121	0.206096201
miR-423	*miR-423-3p*	76.001	93.001	14.001	31.001	69.001	72.001	0.370644098	0.406691103	0.907023921
	miR-423-5p	33.001	47.001	14.001	10.001	22.001	27.001	0.719638751	0.880709989	0.484248178
miR-425	*miR-425-5p*	68.001	88.001	10.001	29.001	47.001	39.001	0.29355697	0.880709989	0.285319522
miR-429	*miR-429-3p*	1181.001	1362.001	264.001	296.001	572.001	567.001	0.171302727	0.868303619	0.06612475
miR-434	*miR-434-3p*	32.001	49.001	23.001	57.001	131.001	250.001	0.043333249	0.069936363	0.00011696
miR-449	*miR-449a-5p*	8.001	20.001	1.001	3.001	18.001	8.001	0.312796486	0.319551049	0.849817974
miR-450	*miR-450a-5p*	12.001	32.001	5.001	7.001	25.001	44.001	0.817939789	0.172577131	0.094484217
miR-451	*miR-451*	35.001	45.001	5.001	8.001	49.001	95.001	0.226721841	0.025437926	0.062765488
miR-455	*miR-455-5p*	13.001	23.001	6.001	6.001	4.001	4.001	0.96616353	0.11445038	0.036741112
miR-465	*miR-465a-5p*	1.001	1.001	2.001	3.001	18.001	12.001	0.043333249	0.291524728	0.000838379
	miR-465c-5p	3.001	6.001	7.001	18.001	35.001	13.001	0.023338489	0.805598074	0.021226464
miR-467	*miR-467a-5p*	76.001	108.001	10.001	12.001	7.001	13.001	0.014841001	0.11154413	6.11264E-05
	miR-467d-5p	15.001	9.001	0.001	1.001	2.001	2.001	0.064624836	0.855935899	0.073314379
miR-470	*miR-470-5p*	13.001	31.001	25.001	64.001	95.001	54.001	0.006600332	0.694257026	0.007621196
miR-484	*miR-484*	26.001	32.001	8.001	16.001	27.001	17.001	0.845082443	0.776719402	0.825455892
miR-486	*miR-486-5p*	78.501	97.001	33.501	17.501	271.501	675.501	0.661487973	0.001012289	0.000198657
miR-497	*miR-497-5p*	129.001	133.001	24.001	48.001	44.001	78.001	0.385199839	0.595852882	0.0762044
miR-501	*miR-501-3p*	57.001	84.001	21.001	28.001	58.001	59.001	0.944072003	0.875541391	0.905007683
miR-532	*miR-532-5p*	128.001	157.001	42.001	31.001	69.001	98.001	0.286342953	0.903974688	0.268484448
miR-541	*miR-541-5p*	1.001	5.001	1.001	3.001	17.001	18.001	0.378727843	0.135821769	0.005888885
miR-574	*miR-574-3p*	61.001	71.001	8.001	11.001	12.001	21.001	0.064882036	0.702164766	0.006780691
miR-582	*miR-582-3p*	2.001	23.001	0.001	2.001	0.001	2.001	0.411449842	0.635335614	0.109011405
	miR-582-5p	10.001	13.001	0.001	0.001	2.001	4.001	0.015227193	0.294215526	0.137007054
miR-652	*miR-652-3p*	22.001	29.001	20.001	13.001	18.001	18.001	0.29355697	0.287156641	0.809385591
miR-669	*miR-669a-5p*	13.66766667	11.33433333	1.001	0.501	1.501	1.834333333	0.043333249	0.853412942	0.009983922
	miR-669c-5p	10.001	11.001	2.001	1.001	2.001	3.001	0.249252552	0.725352283	0.062765488
	miR-669p-5p	13.66766667	11.33433333	1.001	0.501	1.501	1.834333333	0.043333249	0.853412942	0.009983922
miR-671	*miR-671-3p*	7.001	14.001	0.001	2.001	4.001	2.001	0.11468791	0.841437433	0.148227216
miR-672	*miR-672-5p*	152.001	144.001	20.001	22.001	23.001	23.001	0.032460784	0.205394928	0.000285059
miR-676	*miR-676-3p*	23.001	17.001	6.001	8.001	11.001	14.001	0.964462838	0.754920726	0.614519748
miR-720	*miR-720*	13.001	21.001	2.001	11.001	5.001	5.001	0.854382825	0.337293646	0.114086528
miR-741	*miR-741-3p*	7.001	10.001	9.001	44.001	29.001	21.001	0.006600332	0.296519788	0.039557738

miR-743	*miR-743b-3p*	5.001	8.001	7.001	28.001	58.001	14.001	0.055719926	0.856017331	0.049133431
miR-744	*miR-744-5p*	136.001	156.001	20.001	46.001	42.001	30.001	0.207395911	0.200934175	0.003134955
miR-871	*miR-871-3p*	15.001	27.001	29.001	75.001	74.001	78.001	0.001229604	0.500446429	0.00224783
	miR-871-5p	2.001	2.001	1.001	8.001	20.001	7.001	0.131578713	0.740603361	0.023050554
miR-872	*miR-872-3p*	54.001	66.001	11.001	12.001	18.001	28.001	0.198156321	0.856017331	0.061297746
miR-872	*miR-872-5p*	110.001	168.001	30.001	42.001	54.001	50.001	0.385199839	0.357772646	0.028129266
miR-881	*miR-881-3p*	10.001	9.001	20.001	56.001	95.001	53.001	0.004928654	0.876668333	0.002320534
miR-1198	*miR-1198-5p*	20.001	28.001	4.001	11.001	7.001	19.001	0.793491612	0.778326759	0.393360055
miR-1249	*miR-1249-3p*	14.001	14.001	2.001	5.001	5.001	8.001	0.619385307	0.853412942	0.317169798
miR-1251	*miR-1251-5p*	16.001	18.001	5.001	8.001	2.001	4.001	0.89568274	0.044955727	0.01862306
miR-1839	*miR-1839-5p*	93.001	93.001	22.001	64.001	46.001	58.001	0.756802832	0.294215526	0.315593316
miR-1843	*miR-1843-5p*	34.001	43.001	10.001	22.001	10.001	19.001	0.845082443	0.11445038	0.06612475
	miR-1843b-5p	10.001	10.001	3.001	2.001	4.001	7.001	0.718578284	0.887720304	0.501584088
miR-3096	*miR-3096-5p*	9.001	11.001	4.001	10.001	15.001	25.001	0.368266049	0.777668958	0.109199006
miR-3107	*miR-3107-5p*	78.501	97.001	33.501	17.501	271.501	675.501	0.661487973	0.001012289	0.000198657

* red shading indicates absence of miRNA from that sample (i.e. <10 average number of reads across both technical replicates)
