# Supplementary material for: Next Generation Sequencing Analysis Reveals Segmental Patterns of microRNA Expression in Mouse Epididymal Epithelial Cells
Source: PLoS One. 2015 Aug 13;10(8):e0135605. doi: 10.1371/journal.pone.0135605 (PMC4535982; doi:10.1371/journal.pone.0135605)
Supplement: S3 Table — (PDF) [file pone.0135605.s006.pdf]

S3 Table: Comparison of miRNAs identified within mouse, human and rat epididymal tissue

miRNA Family	miRNA*	Mouse (mmu)	Human[1] (hsa)	Rat[2] (rno)
let-7	*let-7a*	+	+	+
	let-7b	+	+	+
	let-7c	+	+	+
	let-7d	+	+	+
	let-7e	+	+	+
	let-7f	+	+	+
	let-7i	+	+	+
	miR-98	+	+	+
miR-10	*miR-10a*	+	+	+
	miR-10b	+	+	+
	miR-15b	+	+	+
miR-16	*miR-16*	+	+	+
miR-17	*miR-17*	+	+	+
miR-20	*miR-20a*	+	+	+
miR-22	*miR-22*	+	+	+
miR-23	*miR-23a*	+	+	+
	miR-23b	+	+	+
miR-24	*miR-24*	+	+	+
miR-25	*miR-25*	+	+	+
miR-26	*miR-26a*	+	+	+
miR-27	*miR-27a*	+	+	+
	miR-27b	+	+	+
miR-29	*miR-29a*	+	+	+
	miR-29c	+	+	+
miR-30	*miR-30a*	+	+	+
	miR-30b	+	+	+
	miR-30c	+	+	+
	miR-30d	+	+	+
	miR-30e	+	+	+
miR-31	*miR-31*	+	+	+
miR-34	*miR-34a*	+	+	+
	miR-34b	+	+	+
	miR-34c	+	+	+
miR-93	*miR-93*	+	+	+
miR-99	*miR-99a*	+	+	+
	miR-99b	+	+	+
miR-100	*miR-100*	+	+	+
miR-103	*miR-103*	+	+	+
miR-106	*miR-106b*	+	+	+
miR-107	*miR-107*	+	+	+
miR-125	*miR-125a*	+	+	+
	miR-125b	+	+	+
miR-127	*miR-127*	+	+	+
miR-130	*miR-130a*	+	+	+
	miR-130b	+	+	+
miR-132	*miR-132*	+	+	+
miR-133	*miR-133a*	+	+	+
	miR-133b	+	+	+
miR-134	*miR-134*	+	+	+
miR-135a	*miR-135a*	+	+	+
miR-139	*miR-139*	+	+	+
miR-140	*miR-140*	+	+	+
miR-141	*miR-141*	+	+	+
miR-143	*miR-143*	+	+	+
miR-148	*miR-148b*	+	+	+
miR-150	*miR-150*	+	+	+
miR-151	*miR-151*	+	+	+
miR-152	*miR-152*	+	+	+
miR-181	*miR-181a*	+	+	+
	miR-181b	+	+	+
	miR-181c	+	+	+
miR-183	*miR-183*	+	+	+
miR-184	*miR-184*	+	+	+
miR-185	*miR-185*	+	+	+
miR-187	*miR-187*	+	+	+
miR-191	*miR-191*	+	+	+
miR-194	*miR-194*	+	+	+
miR-196	*miR-196a*	+	+	+
miR-199	*miR-199a*	+	+	+
miR-200	*miR-200a*	+	+	+
	miR-200b	+	+	+
	miR-200c	+	+	+
miR-204	*miR-204*	+	+	+
miR-205	*miR-205*	+	+	+
miR-210	*miR-210*	+	+	+
miR-212	*miR-212*	+	+	+
miR-214	*miR-214*	+	+	+
miR-221	*miR-221*	+	+	+
miR-222	*miR-222*	+	+	+
miR-296	*miR-296*	+	+	+
miR-298	*miR-298*	+	+	+
miR-324	*miR-324*	+	+	+
miR-328	*miR-328*	+	+	+
miR-330	*miR-330*	+	+	+
miR-331	*miR-331*	+	+	+
miR-337	*miR-337*	+	+	+
miR-338	*miR-338*	+	+	+
miR-339	*miR-339*	+	+	+
miR-342	*miR-342*	+	+	+
miR-345	*miR-345*	+	+	+
miR-361	*miR-361*	+	+	+
miR-365	*miR-365*	+	+	+
miR-382	*miR-382*	+	+	+
miR-409	*miR-409*	+	+	+
miR-485	*miR-485*	+	+	+
miR-487	*miR-487b*	+	+	+
miR-501	*miR-501*	+	+	+
let-7	*let-7a-1*	+	-	-
	let-7c-1	+	-	-
	let-7c-2	+	-	-
	let-7f-1	+	-	-
	let-7g	+	+	-
	let-7j	+	-	-
miR-1	*miR-1*	-	-	+
	miR-1a	+	-	-
miR-7	*miR-7*	-	-	+
	miR-7a-1	+	-	-
	miR-7b	-	-	+
miR-9	*miR-9*	+	-	+
miR-15	*miR-15a*	+	-	-
miR-16	*miR-16-2*	-	+	-
miR-18	*miR-18*	-	-	+
	miR-18a	+	+	-
miR-19	*miR-19a*	+	-	+
	miR-19b	+	-	+
	miR-19b-1	-	+	-
miR-20	*miR-20b*	-	+	+

Comparison

**Mouse + rat + human**	Total Conserved	97 / 463
	Conserved (%)	21
**Mouse + Rat**		
	Total Conserved	143 / 341
	Conserved (%)	42
**Mouse + Human**		
	Total Conserved	131 / 417
	Conserved (%)	31

miR-21	*miR-21*	-	+	+
	miR-21a	+	-	-
miR-24	*miR-24-2*	+	-	-
miR-26	*miR-26a-2*	+	-	-
	miR-26b	+	-	+
miR-28	*miR-28*	-	+	+
	miR-28a	+	-	-
miR-29	*miR-29b*	+	-	+
	miR-29b-1	-	+	-
	miR-29b-2	-	+	-
miR-30	*miR-30c-1*	+	-	-
	miR-30c-2	+	+	-
miR-32	*miR-32*	+	-	+
miR-33	*miR-33*	+	-	+
miR-92	*miR-92*	-	-	+
	miR-92a	+	+	-
	miR-92a-1	+	+	-
	miR-92a-2	-	+	-
	miR-92b	+	+	-
miR-96	*miR-96*	+	-	+
miR-101	*miR-101a*	+	-	+
	miR-101b	+	-	+
miR-106	*miR-106a*	-	+	-
miR-122	*miR-122a*	-	-	+
miR-124	*miR-124*	+	-	-
	miR-124a	-	-	+
miR-125	*miR-125b-1*	+	+	-
	miR-125b-2	+	+	-
miR-126	*miR-126*	-	+	+
	miR-126a	+	-	-
miR-128	*miR-128*	+	-	-
	miR-128a	-	-	+
	miR-128b	-	-	+
miR-129	*miR-129*	-	+	+
miR-135	*miR-135a-2*	+	-	-
	miR-135b	+	-	+
miR-136	*miR-136*	+	-	+
miR-137	*miR-137*	+	-	+
miR-138	*miR-138*	+	-	+
	miR-138-1	-	+	-
miR-142	*miR-142*	-	-	+
	miR-142a	+	-	-
miR-144	*miR-144*	+	-	+
miR-145	*miR-145*	-	+	+
	miR-145a	+	-	-
miR-146	*miR-146*	-	-	+
	miR-146a	+	+	-
	miR-146b	+	+	-
miR-148	*miR-148a*	+	-	-
miR-149	*miR-149*	+	+	-
miR-153	*miR-153*	+	-	+
miR-154	*miR-154*	+	-	+
miR-155	*miR-155*	+	+	-
miR-181	*miR-181a-1*	+	-	-
	miR-181a-2	-	+	-
miR-181	*miR-181d*	+	+	-
miR-182	*miR-182*	+	+	-
miR-186	*miR-186*	+	-	+
miR-188	*miR-188*	+	+	-
miR-189	*miR-189*	-	-	+
miR-190	*miR-190*	-	-	+
	miR-190a	+	-	-
miR-192	*miR-192*	+	-	+
miR-193	*miR-193*	-	-	+
	miR-193a	+	+	-
	miR-193b	+	+	-
miR-195	*miR-195*	-	+	+
	miR-195a	+	-	-
miR-196	*miR-196a-2*	+	-	-
	miR-196b	+	-	+
miR-197	*miR-197*	-	+	-
miR-199	*miR-199b*	+	+	-
miR-202	*miR-202*	-	+	-
miR-203	*miR-203*	+	-	+
miR-206	*miR-206*	+	-	+
miR-207	*miR-207*	-	-	+
miR-208	*miR-208*	-	-	+
	miR-208b	+	-	-
miR-211	*miR-211*	+	-	+
miR-215	*miR-215*	-	-	+
miR-216	*miR-216*	-	-	+
miR-217	*miR-217*	-	-	+
miR-218	*miR-218*	+	-	+
	miR-218-2	-	+	-
miR-219	*miR-219*	-	-	+
miR-223	*miR-223*	+	-	+
miR-224	*miR-224*	-	-	+
miR-290	*miR-290*	-	-	+
miR-291	*miR-291*	-	-	+
miR-292	*miR-292*	-	-	+
miR-297	*miR-297*	-	-	+
miR-299	*miR-299*	-	-	+
	miR-299a	+	-	-
miR-300	*miR-300*	+	-	+
miR-301	*miR-301*	-	-	+
miR-301	*miR-301a*	+	-	-
	miR-301b	+	-	-
miR-320	*miR-320*	+	-	+
	miR-320a	-	+	-
	miR-320b	-	+	-
	miR-320c	-	+	-
	miR-320d	-	+	-
miR-322	*miR-322*	+	-	+
miR-323	*miR-323*	-	-	+
miR-325	*miR-325*	-	-	+
miR-326	*miR-326*	+	-	+
miR-327	*miR-327*	-	-	+
miR-329	*miR-329*	+	-	+
miR-333	*miR-333*	-	-	+
miR-335	*miR-335*	+	-	+
miR-336	*miR-336*	-	-	+
miR-340	*miR-340*	+	-	+
miR-341	*miR-341*	+	-	+
miR-343	*miR-343*	-	-	+
miR-344	*miR-344*	-	-	+
miR-346	*miR-346*	-	+	+
miR-347	*miR-347*	-	-	+
miR-349	*miR-349*	-	-	+
miR-350	*miR-350*	+	-	+

miR-351	*miR-351*	+	-	+
miR-352	*miR-352*	-	-	+
miR-362	*miR-362*	+	+	-
miR-363	*miR-363*	-	+	+
miR-369	*miR-369*	+	-	+
miR-370	*miR-370*	-	+	+
miR-371	*miR-371*	-	+	-
miR-374	*miR-374*	-	-	+
	miR-374b	+	-	-
miR-375	*miR-375*	+	+	-
miR-376	*miR-376a*	+	-	+
	miR-376b	+	-	+
	miR-376c	+	-	+
miR-377	*miR-377*	-	+	+
miR-378	*miR-378*	-	+	+
	miR-378a	+	-	-
	miR-378b	+	-	-
	miR-378c	+	-	-
	miR-378d	+	-	-
miR-379	*miR-379*	+	-	+
miR-381	*miR-381*	+	-	+
miR-383	*miR-383*	-	+	+
miR-410	*miR-410*	+	-	-
miR-411	*miR-411*	+	-	-
miR-412	*miR-412*	-	-	+
miR-421	*miR-421*	+	-	+
miR-422	*miR-422a*	-	+	-
	miR-422b	-	-	+
miR-423	*miR-423*	+	+	-
miR-424	*miR-424*	-	+	+
miR-425	*miR-425*	+	+	-
miR-429	*miR-429*	+	-	+
miR-431	*miR-431*	+	-	+
miR-432	*miR-432*	-	+	-
miR-433	*miR-433*	-	+	+
miR-434	*miR-434*	+	-	-
miR-448	*miR-448*	-	-	+
miR-449	*miR-449*	-	-	+
	miR-449a	+	-	-
miR-450	*miR-450*	-	-	+
	miR-450a	+	-	-
	miR-450b	+	-	-
miR-451	*miR-451*	-	-	+
	miR-451a	+	-	-
miR-455	*miR-455*	+	+	-
miR-463	*miR-463*	+	-	-
miR-465	*miR-465a*	+	-	-
	miR-465b	+	-	-
	miR-465c	+	-	-
miR-466	*miR-466b*	+	-	-
	miR-466c	+	-	-
	miR-466g	+	-	-
	miR-466p	+	-	-
miR-467	*miR-467a*	+	-	-
	miR-467b	+	-	-
	miR-467c	+	-	-
	miR-467d	+	-	-
	miR-467e	+	-	-
miR-470	*miR-470*	+	-	-
miR-471	*miR-471*	+	-	-
miR-483	*miR-483*	-	+	+
miR-484	*miR-484*	+	+	-
miR-486	*miR-486*	-	+	-
	miR-486a	+	-	-
	miR-486b	+	-	-
miR-489	*miR-489*	-	+	+
miR-491	*miR-491*	-	+	-
miR-492	*miR-492*	-	+	-
miR-493	*miR-493*	-	-	+
miR-494	*miR-494*	-	+	+
miR-495	*miR-495*	-	+	-
miR-497	*miR-497*	-	+	+
	miR-497a	+	-	-
miR-499	*miR-499*	+	-	+
miR-500	*miR-500*	+	+	-
miR-502	*miR-502*	-	+	-
miR-503	*miR-503*	-	+	+
miR-504	*miR-504*	-	+	-
miR-505	*miR-505*	-	+	+
miR-508	*miR-508*	-	+	-
miR-509	*miR-509*	-	+	-
miR-509	*miR-509-3*	-	+	-
miR-511	*miR-511*	+	-	-
miR-513	*miR-513a*	-	+	-
miR-517	*miR-517*	-	+	-
miR-519	*miR-519b*	-	+	-
miR-520	*miR-520b*	-	+	-
miR-532	*miR-532*	+	+	-
miR-539	*miR-539*	-	+	+
miR-540	*miR-540*	-	-	+
miR-541	*miR-541*	+	-	+
miR-542	*miR-542*	-	+	+
miR-543	*miR-543*	-	+	+
miR-548	*miR-548a*	-	+	-
	miR-548c	-	+	-
miR-550	*miR-550*	-	+	-
miR-551	*miR-551b*	-	+	-
miR-559	*miR-559*	-	+	-
miR-570	*miR-570*	-	+	-
miR-572	*miR-572*	-	+	-
miR-574	*miR-574*	+	+	-
miR-582	*miR-582*	+	-	-
miR-589	*miR-589*	-	+	-
miR-596	*miR-596*	-	+	-
miR-598	*miR-598*	+	-	-
miR-602	*miR-602*	-	+	-
miR-603	*miR-603*	-	+	-
miR-615	*miR-615*	+	+	-
miR-621	*miR-621*	-	+	-
miR-623	*miR-623*	-	+	-
miR-625	*miR-625*	-	+	-
miR-628	*miR-628*	-	+	-
miR-629	*miR-629*	-	+	-
miR-635	*miR-635*	-	+	-
miR-636	*miR-636*	-	+	-
miR-638	*miR-638*	-	+	-
miR-639	*miR-639*	-	+	-
miR-641	*miR-641*	-	+	-
miR-652	*miR-652*	+	+	-

miR-654	*miR-654*	-	+	-
miR-659	*miR-659*	-	+	-
miR-663	*miR-663*	-	+	-
	miR-663b	-	+	-
miR-664	*miR-664*	-	+	+
miR-665	*miR-665*	-	+	-
miR-669	*miR-669a*	+	-	-
	miR-669c	+	-	-
	miR-669f	+	-	-
	miR-669l	+	-	-
	miR-669o	+	-	-
	miR-669p	+	-	-
miR-671	*miR-671*	+	+	-
miR-672	*miR-672*	+	-	-
miR-674	*miR-674*	+	-	-
miR-675	*miR-675*	-	+	-
miR-676	*miR-676*	+	-	-
miR-677	*miR-677*	+	-	-
miR-708	*miR-708*	+	-	-
miR-720	*miR-720*	-	+	-
miR-741	*miR-741*	+	-	-
miR-743	*miR-743a*	+	-	-
	miR-743b	+	-	-
miR-744	*miR-744*	+	+	-
miR-760	*miR-760*	-	+	-
miR-766	*miR-766*	-	+	-
miR-768	*miR-768*	-	+	-
miR-769	*miR-769*	-	+	-
miR-802	*miR-802*	-	+	-
miR-871	*miR-871*	+	-	-
miR-872	*miR-872*	+	-	-
miR-874	*miR-874*	+	+	-
miR-877	*miR-877*	-	+	-
miR-878	*miR-878*	+	-	-
miR-880	*miR-880*	+	-	-
miR-881	*miR-881*	+	-	-
miR-883	*miR-883a*	+	-	-
miR-885	*miR-885*	-	+	-
miR-886	*miR-886*	-	+	-
miR-887	*miR-887*	-	+	-
miR-890	*miR-890*	-	+	-
miR-891	*miR-891a*	-	+	-
	miR-891b	-	+	-
	miR-892a	-	+	-
	miR-892b	-	+	-
miR-921	*miR-921*	-	+	-
miR-923	*miR-923*	-	+	-
miR-933	*miR-933*	-	+	-
miR-935	*miR-935*	-	+	-
miR-938	*miR-938*	-	+	-
miR-939	*miR-939*	-	+	-
miR-940	*miR-940*	-	+	-
miR-941	*miR-941*	-	+	-
miR-943	*miR-943*	-	+	-
miR-1180	*miR-1180*	-	+	-
miR-1181	*miR-1181*	-	+	-
miR-1184	*miR-1184*	-	+	-
miR-1198	*miR-1198*	+	-	-
miR-1202	*miR-1202*	-	+	-
miR-1207	*miR-1207*	-	+	-
miR-1224	*miR-1224*	-	+	-
miR-1225	*miR-1225*	-	+	-
miR-1226	*miR-1226*	-	+	-
miR-1228	*miR-1228*	-	+	-
miR-1229	*miR-1229*	-	+	-
miR-1231	*miR-1231*	-	+	-
miR-1234	*miR-1234*	-	+	-
miR-1236	*miR-1236*	-	+	-
miR-1238	*miR-1238*	-	+	-
miR-1244	*miR-1244*	-	+	-
miR-1246	*miR-1246*	-	+	-
miR-1247	*miR-1247*	+	+	-
miR-1249	*miR-1249*	+	+	-
miR-1251	*miR-1251*	+	-	-
miR-1254	*miR-1254*	-	+	-
miR-1260	*miR-1260*	-	+	-
miR-1263	*miR-1263*	-	+	-
miR-1267	*miR-1267*	-	+	-
miR-1268	*miR-1268*	-	+	-
miR-1270	*miR-1270*	-	+	-
miR-1271	*miR-1271*	-	+	-
miR-1272	*miR-1272*	-	+	-
miR-1274	*miR-1274a*	-	+	-
	miR-1274b	-	+	-
miR-1275	*miR-1275*	-	+	-
miR-1280	*miR-1280*	-	+	-
miR-1281	*miR-1281*	-	+	-
miR-1287	*miR-1287*	-	+	-
miR-1291	*miR-1291*	-	+	-
miR-1292	*miR-1292*	-	+	-
miR-1296	*miR-1296*	-	+	-
miR-1299	*miR-1299*	-	+	-
miR-1300	*miR-1300*	-	+	-
miR-1301	*miR-1301*	-	+	-
miR-1307	*miR-1307*	-	+	-
miR-1308	*miR-1308*	-	+	-
miR-1323	*miR-1323*	-	+	-
miR-1825	*miR-1825*	-	+	-
miR-1826	*miR-1826*	-	+	-
miR-1839	*miR-1839*	+	-	-
miR-1843	*miR-1843a*	+	-	-
	miR-1843b	+	-	-
miR-1981	*miR-1981*	+	-	-
miR-3068	*miR-3068*	+	-	-
miR-3082	*miR-3082*	+	-	-
miR-3475	*miR-3475*	+	-	-
miR-3535	*miR-3535*	+	-	-
miR-8114	*miR-8114*	+	-	-

*All miRNAs appearing above row 100 represent those that have been identified as being conserved in the epididymis of the mouse, rat, and human. Those in rows 100-465 have been identified in either 1 or 2 of these species.

1. Belleannee, C., Calvo, E., Thimon, V., Cyr, D. G., Legare, C., Garneau, L. and Sullivan, R. (2012). Role of microRNAs in controlling gene expression in different segments of the human epididymis. PLoS One 7, e34996.

2. Ma, W., Xie, S., Ni, M., Huang, X., Hu, S., Liu, Q., Liu, A., Zhang, J. and Zhang, Y. (2012). MicroRNA-29a inhibited epididymal epithelial cell proliferation by targeting nuclear autoantigenic sperm protein (NASP). J Biol Chem 287, 10189-99.
