# Supplementary material for: Next Generation Sequencing Analysis Reveals Segmental Patterns of microRNA Expression in Mouse Epididymal Epithelial Cells
Source: PLoS One. 2015 Aug 13;10(8):e0135605. doi: 10.1371/journal.pone.0135605 (PMC4535982; doi:10.1371/journal.pone.0135605)
Supplement: S4 Table — (PDF) [file pone.0135605.s007.pdf]

S4 Table. Comparison of segmental expression of miRNAs identified within mouse, human and rat epididymal tissue

MicroRNA Family	miRNA*	Mouse (mmu)			Human[1] (hsa)			Rat[2] (rno)		
		Caput	Corpus	Cauda	Caput	Corpus	Cauda	Caput	Corpus	Cauda
let-7	*let-7a*	+	+	+	+	+	+	+	+	+
	let-7b	+	+	+	+	+	+	+	+	+
	let-7c	+	+	+	+	+	+	+	+	+
	let-7d	+	+	+	+	+	+	+	+	+
	let-7e	+	+	+	+	+	+	+	+	+
	let-7f	+	+	+	+	+	+	+	+	+
	let-7i	+	+	+	+	+	+	+	+	+
	miR-98	+	+	+	+	+	+	+	+	+
miR-10	*miR-10a*	+	+	+	+	+	+	+	+	+
	miR-10b	+	+	+	+	+	+	+	+	+
	miR-15b	+	+	+	+	+	+	+	+	+
miR-16	*miR-16*	+	+	+	+	+	+	+	+	+
miR-17	*miR-17*	+	+	+	+	+	+	+	+	+
miR-22	*miR-22*	+	+	+	+	+	+	+	+	+
miR23	*miR-23a*	+	+	+	+	+	+	+	+	+
	miR-23b	+	+	+	+	+	+	+	+	+
miR-24	*miR-24*	+	+	+	+	+	+	+	+	+
miR-25	*miR-25*	+	+	+	+	+	+	+	+	+
miR-26	*miR-26a*	+	+	+	+	+	+	+	+	+
miR-27	*miR-27a*	+	+	+	+	+	+	+	+	+
	miR-27b	+	+	+	+	+	+	+	+	+
miR-29	*miR-29a*	+	+	+	+	+	+	+	+	+
miR-30	*miR-30a*	+	+	+	+	+	+	+	+	+
	miR-30b	+	+	+	+	+	+	+	+	+
	miR-30c	+	+	+	+	+	+	+	+	+
	miR-30d	+	+	+	+	+	+	+	+	+
miR-31	*miR-31*	+	+	+	+	+	+	+	+	+
miR-34	*miR-34a*	+	+	+	+	+	+	+	+	+
	miR-34b	+	+	+	+	+	+	+	+	+
	miR-34c	+	+	+	+	+	+	+	+	+
miR-93	*miR-93*	+	+	+	+	+	+	+	+	+
miR-99	*miR-99a*	+	+	+	+	+	+	+	+	+
	miR-99b	+	+	+	+	+	+	+	+	+
miR-100	*miR-100*	+	+	+	+	+	+	+	+	+
miR-103	*miR-103*	+	+	+	+	+	+	+	+	+
miR-106	*miR-106b*	+	+	+	+	+	+	+	+	+
miR-107	*miR-107*	+	+	+	+	+	+	+	+	+
miR-125	*miR-125a*	+	+	+	+	+	+	+	+	+
	miR-125b	+	+	+	+	+	+	+	+	+
miR-127	*miR-127*	+	+	+	+	+	+	+	+	+
mir-130	*miR-130a*	+	+	+	+	+	+	+	+	+
	miR-130b	+	+	+	+	+	+	+	+	+
miR-132	*miR-132*	+	+	+	+	+	+	+	+	+
miR-133	*miR-133a*	+	+	+	+	+	+	+	+	+
miR-140	*miR-140*	+	+	+	+	+	+	+	+	+
miR-143	*miR-143*	+	+	+	+	+	+	+	+	+
miR-148	*miR-148b*	+	+	+	+	+	+	+	+	+
miR-150	*miR-150*	+	+	+	+	+	+	+	+	+
miR-151	*miR-151*	+	+	+	+	+	+	+	+	+
miR-152	*miR-152*	+	+	+	+	+	+	+	+	+
miR-181	*miR-181a*	+	+	+	+	+	+	+	+	+
	miR-181b	+	+	+	+	+	+	+	+	+
	miR-181c	+	+	+	+	+	+	+	+	+
miR-183	*miR-183*	+	+	+	+	+	+	+	+	+
miR-185	*miR-185*	+	+	+	+	+	+	+	+	+
miR-187	*miR-187*	+	+	+	+	+	+	+	+	+
miR-191	*miR-191*	+	+	+	+	+	+	+	+	+
miR-199	*miR-199a*	+	+	+	+	+	+	+	+	+
miR-200	*miR-200b*	+	+	+	+	+	+	+	+	+
	miR-200c	+	+	+	+	+	+	+	+	+
miR-205	*miR-205*	+	+	+	+	+	+	+	+	+
miR-210	*miR-210*	+	+	+	+	+	+	+	+	+
miR-214	*miR-214*	+	+	+	+	+	+	+	+	+
miR-221	*miR-221*	+	+	+	+	+	+	+	+	+
miR-222	*miR-222*	+	+	+	+	+	+	+	+	+
miR-296	*miR-296*	+	+	+	+	+	+	+	+	+
miR-298	*miR-298*	+	+	+	+	+	+	+	+	+
miR-324	*miR-324*	+	+	+	+	+	+	+	+	+
miR-328	*miR-328*	+	+	+	+	+	+	+	+	+
miR-330	*miR-330*	+	+	+	+	+	+	+	+	+
miR-331	*miR-331*	+	+	+	+	+	+	+	+	+
miR-337	*miR-337*	+	+	+	+	+	+	+	+	+
miR-338	*miR-338*	+	+	+	+	+	+	+	+	+
miR-339	*miR-339*	+	+	+	+	+	+	+	+	+
miR-342	*miR-342*	+	+	+	+	+	+	+	+	+
miR-361	*miR-361*	+	+	+	+	+	+	+	+	+
miR-382	*miR-382*	+	+	+	+	+	+	+	+	+
miR-409	*miR-409*	+	+	+	+	+	+	+	+	+
miR-501	*miR-501*	+	+	+	+	+	+	+	+	+
let-7	*let-7a-1*	+	+	+	-	-	-	-	-	-
	let-7c-1	+	+	+	-	-	-	-	-	-
	let-7c-2	+	+	+	-	-	-	-	-	-
	let-7f-1	+	+	+	-	-	-	-	-	-
	let-7g	+	+	+	+	+	+	-	-	-
	let-7j	+	+	+	-	-	-	-	-	-
miR-1	*miR-1*	-	-	-	-	-	-	+	+	+
	miR-1a	+	+	+	-	-	-	-	-	-
miR-7	*miR-7*	-	-	-	-	-	-	+	+	+
	miR-7a-1	+	+	+	-	-	-	-	-	-
	miR-7b	-	-	-	-	-	-	+	+	+
miR-9	*miR-9*	+	+	+	-	-	-	+	+	+
miR-15	*miR-15a*	+	+	+	-	-	-	-	-	-
miR-16	*miR-16-2*	-	-	-	+	-	-	-	-	-
miR-18	*miR-18*	-	-	-	-	-	-	+	+	+
	miR-18a	+	+	+	+	+	+	-	-	-
miR-19	*miR-19a*	+	+	+	-	-	-	+	+	+
	miR-19b	+	+	+	-	-	-	+	+	+
	miR-19b-1	-	-	-	-	+	-	-	-	-
miR-20	*miR-20a*	+	+	+	+	+	-	+	+	+
	miR-20b	-	-	-	+	+	-	+	+	+
miR-21	*miR-21*	-	-	-	-	-	+	+	+	+
	miR-21a	+	+	+	-	-	-	-	-	-
miR-24	*miR-24-2*	+	+	+	-	-	-	-	-	-
miR-26	*miR-26a-2*	+	-	+	-	-	-	-	-	-
	miR-26b	+	+	+	-	-	-	+	+	+
miR-28	*miR-28*	-	-	-	+	+	+	+	+	+
	miR-28a	+	+	+	-	-	-	-	-	-
miR-29	*miR-29b*	+	+	+	-	-	-	+	+	+
	miR-29b-1	-	-	-	+	+	-	-	-	-
	miR-29b-2	-	-	-	+	+	+	-	-	-
	miR-29c	+	+	+	-	+	-	+	+	+
miR-30	*miR-30c-1*	+	+	+	-	-	-	-	-	-
	miR-30c-2	+	+	+	+	+	-	-	-	-
	miR-30e	+	+	+	+	-	-	+	+	+
miR-32	*miR-32*	+	-	-	-	-	-	+	+	+
miR-33	*miR-33*	+	-	-	-	-	-	+	+	+
miR-92	*miR-92*	-	-	-	-	-	-	+	+	+
	miR-92a	+	+	+	+	+	+	-	-	-
	miR-92a-1	+	+	+	+	-	+	-	-	-
	miR-92a-2	-	-	-	+	-	+	-	-	-
	miR-92b	+	+	+	+	+	+	-	-	-
miR-96	*miR-96*	+	+	+	-	-	-	+	+	+
miR-101	*miR-101a*	+	+	+	-	-	-	+	+	+
	miR-101b	+	+	+	-	-	-	+	+	+
miR-106	*miR-106a*	-	-	-	+	+	+	-	-	-

Comparison

**Mouse + rat + human**	Total Conserved	97 / 463
	Conserved (%)	21
**Mouse + Rat**	Total Conserved	143 / 341
	Conserved (%)	42
**Mouse + Human**	Total Conserved	131 / 417
	Conserved (%)	31

miR-122	miR-122a	-	-	-	-	-	-	+	+	+
miR-124	miR-124	-	-	+	-	-	-	-	-	-
	miR-124a	-	-	-	-	-	-	+	+	+
miR-125	miR-125b-1	+	+	+	+	+	+	-	-	-
	miR-125b-2	+	+	+	+	+	+	-	-	-
miR-126	miR-126	-	-	-	+	+	+	+	+	+
	miR-126a	+	+	+	-	-	-	-	-	-
miR-128	miR-128	+	+	+	-	-	-	-	-	-
	miR-128a	-	-	-	-	-	-	+	+	+
	miR-128b	-	-	-	-	-	-	+	+	+
miR-129	miR-129	-	-	-	+	-	+	+	+	+
miR-133	miR-133b	-	-	+	+	+	+	+	+	+
miR-134	miR-134	-	-	+	+	+	+	+	+	+
miR-135	miR-135a	+	+	+	+	+	-	+	+	+
	miR-135a-2	+	-	-	-	-	-	-	-	-
	miR-135b	+	+	-	-	-	-	+	+	+
miR-136	miR-136	+	+	+	-	-	-	+	+	+
miR-137	miR-137	-	-	+	-	-	-	+	+	+
miR-138	miR-138	+	+	+	-	-	-	+	+	+
	miR-138-1	-	-	-	+	+	+	-	-	-
miR-139	miR-139	-	+	+	+	+	+	+	+	+
miR-141	miR-141	+	+	+	-	+	-	+	+	+
miR-142	miR-142	-	-	-	-	-	-	+	+	+
	miR-142a	+	+	+	-	-	-	-	-	-
miR-144	miR-144	-	+	+	-	-	-	+	+	+
miR-145	miR-145	-	-	-	+	+	+	+	+	+
	miR-145a	+	+	+	-	-	-	-	-	-
miR-146	miR-146	-	-	-	-	-	-	+	+	+
	miR-146a	+	+	+	+	+	-	-	-	-
	miR-146b	+	+	+	+	+	+	-	-	-
miR-148	miR-148a	+	+	+	-	-	-	-	-	-
miR-149	miR-149	+	+	+	+	+	+	-	-	-
miR-153	miR-153	+	+	+	-	-	-	+	+	+
miR-154	miR-154	-	+	+	-	-	-	+	+	+
miR-155	miR-155	-	-	+	+	+	+	-	-	-
miR-181	miR-181a-1	+	+	+	-	-	-	-	-	-
	miR-181a-2	-	-	-	+	+	+	-	-	-
	miR-181d	+	+	+	+	+	+	-	-	-
miR-182	miR-182	+	+	+	+	+	+	-	-	-
miR-184	miR-184	+	+	+	+	-	+	+	+	+
miR-186	miR-186	+	+	+	-	-	-	+	+	+
miR-188	miR-188	-	-	+	+	+	+	-	-	-
miR-189	miR-189	-	-	-	-	-	-	+	+	+
miR-190	miR-190	-	-	-	-	-	-	+	+	+
	miR-190a	+	+	+	-	-	-	-	-	-
miR-192	miR-192	+	+	+	-	-	-	+	+	+
miR-193	miR-193	-	-	-	-	-	-	+	+	+
	miR-193a	+	+	+	+	+	+	-	-	-
	miR-193b	+	+	+	+	+	+	-	-	-
miR-194	miR-194	+	+	+	+	-	-	+	+	+
miR-195	miR-195	-	-	-	+	+	+	+	+	+
	miR-195a	+	+	+	-	-	-	-	-	-
miR-196	miR-196a	+	+	+	-	-	+	+	+	+
	miR-196a-2	-	+	+	-	-	-	-	-	-
	miR-196b	+	+	+	-	-	-	+	+	+
miR-197	miR-197	-	-	-	+	+	+	-	-	-
miR-199	miR-199b	+	+	+	+	+	+	-	-	-
miR-200	miR-200a	+	+	+	+	+	-	+	+	+
miR-202	miR-202	-	-	-	+	-	+	-	-	-
miR-203	miR-203	+	+	+	-	-	-	+	+	+
miR-204	miR-204	+	+	+	+	+	-	+	+	+
miR-206	miR-206	-	-	+	-	-	-	+	+	+
miR-207	miR-207	-	-	-	-	-	-	+	+	+
miR-208	miR-208	-	-	-	-	-	-	+	+	+
	miR-208b	-	-	+	-	-	-	-	-	-
miR-211	miR-211	-	+	-	-	-	-	+	+	+
miR-212	miR-212	-	-	+	+	+	+	+	+	+
miR-215	miR-215	-	-	-	-	-	-	+	+	+
miR-216	miR-216	-	-	-	-	-	-	+	+	+
miR-217	miR-217	-	-	-	-	-	-	+	+	+
miR-218	miR-218	+	+	+	-	-	-	+	+	+
	miR-218-2	-	-	-	-	-	+	-	-	-
miR-219	miR-219	-	-	-	-	-	-	+	+	+
miR-223	miR-223	+	+	+	-	-	-	+	+	+
miR-224	miR-224	-	-	-	-	-	-	+	+	+
miR-290	miR-290	-	-	-	-	-	-	+	+	+
miR-291	miR-291	-	-	-	-	-	-	+	+	+
miR-292	miR-292	-	-	-	-	-	-	+	+	+
miR-297	miR-297	-	-	-	-	-	-	+	+	+
miR-299	miR-299	-	-	-	-	-	-	+	+	+
	miR-299a	-	-	+	-	-	-	-	-	-
miR-300	miR-300	+	+	+	-	-	-	+	+	+
miR-301	miR-301	-	-	-	-	-	-	+	+	+
	miR-301a	+	+	+	-	-	-	-	-	-
	miR-301b	+	-	+	-	-	-	-	-	-
miR-320	miR-320	+	+	+	-	-	-	+	+	+
	miR-320a	-	-	-	+	+	+	-	-	-
	miR-320b	-	-	-	+	+	+	-	-	-
	miR-320c	-	-	-	+	+	+	-	-	-
	miR-320d	-	-	-	+	+	+	-	-	-
miR-322	miR-322	+	+	+	-	-	-	+	+	+
miR-323	miR-323	-	-	-	-	-	-	+	+	+
miR-325	miR-325	-	-	-	-	-	-	+	+	+
miR-326	miR-326	+	+	+	-	-	-	+	+	+
miR-327	miR-327	-	-	-	-	-	-	+	+	+
miR-329	miR-329	+	-	+	-	-	-	+	+	+
miR-333	miR-333	-	-	-	-	-	-	+	+	+
miR-335	miR-335	+	+	+	-	-	-	+	+	+
miR-336	miR-336	-	-	-	-	-	-	+	+	+
miR-340	miR-340	+	+	+	-	-	-	+	+	+
miR-341	miR-341	+	+	+	-	-	-	+	+	+
miR-343	miR-343	-	-	-	-	-	-	+	+	+
miR-344	miR-344	-	-	-	-	-	-	+	+	+
miR-345	miR-345	+	+	-	+	+	+	+	+	+
miR-346	miR-346	-	-	-	+	+	+	+	+	+
miR-347	miR-347	-	-	-	-	-	-	+	+	+
miR-349	miR-349	-	-	-	-	-	-	+	+	+
miR-350	miR-350	+	-	+	-	-	-	+	+	+
miR-351	miR-351	+	+	+	-	-	-	+	+	+
miR-352	miR-352	-	-	-	-	-	-	+	+	+
miR-362	miR-362	+	+	+	+	+	+	-	-	-
miR-363	miR-363	-	-	-	+	+	-	+	+	+
miR-365	miR-365	+	+	+	-	-	+	+	+	+
miR-369	miR-369	-	+	+	-	-	-	+	+	+
miR-370	miR-370	-	-	-	+	+	+	+	+	+
miR-371	miR-371	-	-	-	-	-	+	-	-	-
miR-374	miR-374	-	-	-	-	-	-	+	+	+
	miR-374b	+	+	+	-	-	-	-	-	-
miR-375	miR-375	+	+	+	+	+	+	-	-	-
miR-376	miR-376a	-	-	+	-	-	-	+	+	+
	miR-376b	+	+	+	-	-	-	+	+	+
	miR-376c	+	-	+	-	-	-	+	+	+
miR-377	miR-377	-	-	-	+	+	+	+	+	+
miR-378	miR-378	-	-	-	+	+	+	+	+	+
	miR-378a	+	+	+	-	-	-	-	-	-
	miR-378b	-	-	+	-	-	-	-	-	-
	miR-378c	+	+	+	-	-	-	-	-	-
	miR-378d	+	+	+	-	-	-	-	-	-
miR-379	miR-379	+	+	+	-	-	-	+	+	+

miR-381	miR-381	+	+	+	-	-	-	+	+	+
miR-383	miR-383	-	-	-	+	+	+	+	+	+
miR-410	miR-410	+	+	+	-	-	-	-	-	-
miR-411	miR-411	+	+	+	-	-	-	-	-	-
miR-412	miR-412	-	-	-	-	-	-	+	+	+
miR-421	miR-421	+	+	+	-	-	-	+	+	+
miR-422	miR-422a	-	-	-	+	+	+	-	-	-
	miR-422b	-	-	-	-	-	-	+	+	+
miR-423	miR-423	+	+	+	+	+	+	-	-	-
miR-424	miR-424	-	-	-	+	+	+	+	+	+
miR-425	miR-425	+	+	+	+	+	+	-	-	-
miR-429	miR-429	+	+	+	-	-	-	+	+	+
miR-431	miR-431	-	-	+	-	-	-	+	+	+
miR-432	miR-432	-	-	-	+	+	+	-	-	-
miR-433	miR-433	-	-	-	+	+	+	+	+	+
miR-434	miR-434	+	+	+	-	-	-	-	-	-
miR-448	miR-448	-	-	-	-	-	-	+	+	+
miR-449	miR-449	-	-	-	-	-	-	+	+	+
miR-449	miR-449a	+	+	+	-	-	-	-	-	-
miR-450	miR-450	-	-	-	-	-	-	+	+	+
miR-450	miR-450a	+	+	+	-	-	-	-	-	-
	miR-450b	+	-	-	-	-	-	-	-	-
miR-451	miR-451	-	-	-	-	-	-	+	+	+
	miR-451a	+	+	+	-	-	-	-	-	-
miR-455	miR-455	+	+	+	+	+	+	-	-	-
miR-463	miR-463	-	-	+	-	-	-	-	-	-
miR-465	miR-465a	+	+	+	-	-	-	-	-	-
	miR-465b	+	+	+	-	-	-	-	-	-
	miR-465c	+	+	+	-	-	-	-	-	-
miR-466	miR-466b	+	-	-	-	-	-	-	-	-
	miR-466c	+	-	-	-	-	-	-	-	-
	miR-466g	+	-	-	-	-	-	-	-	-
	miR-466p	+	-	-	-	-	-	-	-	-
miR-467	miR-467a	+	+	+	-	-	-	-	-	-
	miR-467b	+	+	-	-	-	-	-	-	-
	miR-467c	+	+	+	-	-	-	-	-	-
	miR-467d	+	+	-	-	-	-	-	-	-
miR-467	miR-467e	+	+	+	-	-	-	-	-	-
miR-470	miR-470	+	+	+	-	-	-	-	-	-
miR-471	miR-471	-	+	+	-	-	-	-	-	-
miR-483	miR-483	-	-	-	+	+	+	+	+	+
miR-484	miR-484	+	+	+	+	+	-	-	-	-
miR-485	miR-485	-	-	+	+	+	+	+	+	+
miR-486	miR-486	-	-	-	+	+	+	-	-	-
	miR-486a	+	+	+	-	-	-	-	-	-
	miR-486b	+	+	+	-	-	-	-	-	-
miR-487	miR-487b	-	-	+	+	+	+	+	+	+
miR-489	miR-489	-	-	-	+	-	-	+	+	+
miR-491	miR-491	-	-	-	+	+	+	-	-	-
miR-492	miR-492	-	-	-	-	-	+	-	-	-
miR-493	miR-493	-	-	-	-	-	-	+	+	+
miR-494	miR-494	-	-	-	+	+	+	+	+	+
miR-495	miR-495	-	-	-	-	-	+	-	-	-
miR-497	miR-497	-	-	-	+	+	+	+	+	+
	miR-497a	+	+	+	-	-	-	-	-	-
miR-499	miR-499	-	-	+	-	-	-	+	+	+
miR-500	miR-500	+	-	+	+	+	+	-	-	-
miR-502	miR-502	-	-	-	+	+	+	-	-	-
miR-503	miR-503	-	-	-	+	+	+	+	+	+
miR-504	miR-504	-	-	-	+	+	+	-	-	-
miR-505	miR-505	-	-	-	+	+	+	+	+	+
miR-508	miR-508	-	-	-	+	+	+	-	-	-
miR-509	miR-509	-	-	-	+	+	+	-	-	-
	miR-509-3	-	-	-	-	-	+	-	-	-
miR-511	miR-511	+	+	+	-	-	-	-	-	-
miR-513	miR-513a	-	-	-	-	+	-	-	-	-
miR-517	miR-517	-	-	-	-	+	+	-	-	-
miR-519	miR-519b	-	-	-	-	+	+	-	-	-
miR-520	miR-520b	-	-	-	+	+	+	-	-	-
miR-532	miR-532	+	+	+	+	+	+	-	-	-
miR-539	miR-539	-	-	-	-	+	+	+	+	+
miR-540	miR-540	-	-	-	-	-	-	+	+	+
miR-541	miR-541	+	+	+	-	-	-	+	+	+
miR-542	miR-542	-	-	-	+	+	+	+	+	+
miR-543	miR-543	-	-	-	-	+	+	+	+	+
miR-548	miR-548a	-	-	-	+	+	+	-	-	-
	miR-548c	-	-	-	-	-	+	-	-	-
miR-550	miR-550	-	-	-	+	+	+	-	-	-
miR-551	miR-551b	-	-	-	+	-	-	-	-	-
miR-559	miR-559	-	-	-	-	-	+	-	-	-
miR-570	miR-570	-	-	-	-	-	+	-	-	-
miR-572	miR-572	-	-	-	+	+	+	-	-	-
miR-574	miR-574	+	+	+	+	+	+	-	-	-
miR-582	miR-582	+	+	+	-	-	-	-	-	-
miR-589	miR-589	-	-	-	+	+	+	-	-	-
miR-596	miR-596	-	-	-	+	+	+	-	-	-
miR-598	miR-598	+	+	-	-	-	-	-	-	-
miR-602	miR-602	-	-	-	+	+	+	-	-	-
miR-603	miR-603	-	-	-	-	-	+	-	-	-
miR-615	miR-615	+	+	+	+	+	+	-	-	-
miR-621	miR-621	-	-	-	-	+	-	-	-	-
miR-623	miR-623	-	-	-	-	+	+	-	-	-
miR-625	miR-625	-	-	-	-	+	-	-	-	-
miR-628	miR-628	-	-	-	-	+	-	-	-	-
miR-629	miR-629	-	-	-	+	+	-	-	-	-
miR-635	miR-635	-	-	-	+	+	+	-	-	-
miR-636	miR-636	-	-	-	-	+	-	-	-	-
miR-638	miR-638	-	-	-	+	+	+	-	-	-
miR-639	miR-639	-	-	-	-	-	+	-	-	-
miR-641	miR-641	-	-	-	-	-	+	-	-	-
miR-652	miR-652	+	+	+	+	+	+	-	-	-
miR-654	miR-654	-	-	-	+	+	+	-	-	-
miR-659	miR-659	-	-	-	+	+	+	-	-	-
miR-663	miR-663	-	-	-	+	+	+	-	-	-
	miR-663b	-	-	-	+	-	+	-	-	-
miR-664	miR-664	-	-	-	+	+	+	+	+	+
miR-665	miR-665	-	-	-	-	+	+	-	-	-
miR-669	miR-669a	+	-	+	-	-	-	-	-	-
	miR-669c	+	+	+	-	-	-	-	-	-
	miR-669f	+	-	-	-	-	-	-	-	-
	miR-669l	+	-	-	-	-	-	-	-	-
	miR-669o	+	+	+	-	-	-	-	-	-
	miR-669p	+	-	-	-	-	-	-	-	-
miR-671	miR-671	+	+	+	+	+	+	-	-	-
miR-672	miR-672	+	+	+	-	-	-	-	-	-
miR-674	miR-674	+	-	+	-	-	-	-	-	-
miR-675	miR-675	-	-	-	+	+	+	-	-	-
miR-676	miR-676	+	+	+	-	-	-	-	-	-
miR-677	miR-677	-	+	+	-	-	-	-	-	-
miR-708	miR-708	+	+	+	-	-	-	-	-	-
miR-720	miR-720	-	-	-	+	+	+	-	-	-
miR-741	miR-741	+	+	+	-	-	-	-	-	-
miR-743	miR-743a	+	+	+	-	-	-	-	-	-
	miR-743b	+	+	+	-	-	-	-	-	-
miR-744	miR-744	+	+	+	+	+	+	-	-	-
miR-760	miR-760	-	-	-	+	+	-	-	-	-
miR-766	miR-766	-	-	-	+	+	+	-	-	-
miR-768	miR-768	-	-	-	+	+	+	-	-	-
miR-769	miR-769	-	-	-	+	+	-	-	-	-

miR-802	*miR-802*	-	-	-	-	-	+	-	-	-
miR-871	*miR-871*	+	+	+	-	-	-	-	-	-
miR-872	*miR-872*	+	+	+	-	-	-	-	-	-
miR-874	*miR-874*	+	+	+	+	+	+	-	-	-
miR-877	*miR-877*	-	-	-	+	+	+	-	-	-
miR-878	*miR-878*	-	+	+	-	-	-	-	-	-
miR-880	*miR-880*	-	+	+	-	-	-	-	-	-
miR-881	*miR-881*	+	+	+	-	-	-	-	-	-
miR-883	*miR-883a*	-	+	+	-	-	-	-	-	-
miR-885	*miR-885*	-	-	-	+	+	+	-	-	-
miR-886	*miR-886*	-	-	-	+	+	+	-	-	-
miR-887	*miR-887*	-	-	-	+	+	-	-	-	-
miR-890	*miR-890*	-	-	-	-	+	+	-	-	-
miR-891	*miR-891a*	-	-	-	+	+	+	-	-	-
	miR-891b	-	-	-	-	+	+	-	-	-
miR-892	*miR-892a*	-	-	-	-	+	+	-	-	-
	miR-892b	-	-	-	-	+	+	-	-	-
miR-921	*miR-921*	-	-	-	+	+	+	-	-	-
miR-923	*miR-923*	-	-	-	+	+	+	-	-	-
miR-933	*miR-933*	-	-	-	+	+	+	-	-	-
miR-935	*miR-935*	-	-	-	+	+	+	-	-	-
miR-938	*miR-938*	-	-	-	+	+	+	-	-	-
miR-939	*miR-939*	-	-	-	+	+	+	-	-	-
miR-940	*miR-940*	-	-	-	+	+	+	-	-	-
miR-941	*miR-941*	-	-	-	+	+	+	-	-	-
miR-943	*miR-943*	-	-	-	+	-	+	-	-	-
miR-1180	*miR-1180*	-	-	-	+	+	+	-	-	-
miR-1181	*miR-1181*	-	-	-	-	-	+	-	-	-
miR-1184	*miR-1184*	-	-	-	+	+	+	-	-	-
miR-1198	*miR-1198*	+	+	+	-	-	-	-	-	-
miR-1202	*miR-1202*	-	-	-	+	-	+	-	-	-
miR-1207	*miR-1207*	-	-	-	+	+	+	-	-	-
miR-1224	*miR-1224*	-	-	-	+	-	+	-	-	-
miR-1225	*miR-1225*	-	-	-	+	+	+	-	-	-
miR-1226	*miR-1226*	-	-	-	-	-	+	-	-	-
miR-1228	*miR-1228*	-	-	-	+	+	+	-	-	-
miR-1229	*miR-1229*	-	-	-	+	+	+	-	-	-
miR-1231	*miR-1231*	-	-	-	-	-	+	-	-	-
miR-1234	*miR-1234*	-	-	-	+	+	+	-	-	-
miR-1236	*miR-1236*	-	-	-	-	-	+	-	-	-
miR-1238	*miR-1238*	-	-	-	+	+	+	-	-	-
miR-1244	*miR-1244*	-	-	-	+	+	+	-	-	-
miR-1246	*miR-1246*	-	-	-	+	-	+	-	-	-
miR-1247	*miR-1247*	+	+	+	+	+	+	-	-	-
miR-1249	*miR-1249*	+	+	+	+	-	-	-	-	-
miR-1251	*miR-1251*	+	+	+	-	-	-	-	-	-
miR-1254	*miR-1254*	-	-	-	-	+	+	-	-	-
miR-1260	*miR-1260*	-	-	-	+	+	+	-	-	-
miR-1263	*miR-1263*	-	-	-	+	+	+	-	-	-
miR-1267	*miR-1267*	-	-	-	+	+	-	-	-	-
miR-1268	*miR-1268*	-	-	-	+	+	+	-	-	-
miR-1270	*miR-1270*	-	-	-	+	+	-	-	-	-
miR-1271	*miR-1271*	-	-	-	+	+	+	-	-	-
miR-1272	*miR-1272*	-	-	-	+	-	+	-	-	-
miR-1274	*miR-1274a*	-	-	-	-	-	+	-	-	-
	miR-1274b	-	-	-	+	+	+	-	-	-
miR-1275	*miR-1275*	-	-	-	+	+	+	-	-	-
miR-1280	*miR-1280*	-	-	-	+	+	+	-	-	-
miR-1281	*miR-1281*	-	-	-	+	+	+	-	-	-
miR-1287	*miR-1287*	-	-	-	+	+	+	-	-	-
miR-1291	*miR-1291*	-	-	-	-	+	-	-	-	-
miR-1292	*miR-1292*	-	-	-	+	+	+	-	-	-
miR-1296	*miR-1296*	-	-	-	+	+	+	-	-	-
miR-1299	*miR-1299*	-	-	-	+	-	-	-	-	-
miR-1300	*miR-1300*	-	-	-	-	-	+	-	-	-
miR-1301	*miR-1301*	-	-	-	+	+	+	-	-	-
miR-1307	*miR-1307*	-	-	-	+	+	+	-	-	-
miR-1308	*miR-1308*	-	-	-	+	+	+	-	-	-
miR-1323	*miR-1323*	-	-	-	+	+	+	-	-	-
miR-1825	*miR-1825*	-	-	-	+	+	+	-	-	-
miR-1826	*miR-1826*	-	-	-	+	+	+	-	-	-
miR-1839	*miR-1839*	+	+	+	-	-	-	-	-	-
miR-1843	*miR-1843a*	+	+	+	-	-	-	-	-	-
	miR-1843b	+	+	+	-	-	-	-	-	-
miR-1981	*miR-1981*	+	+	+	-	-	-	-	-	-
miR-3068	*miR-3068*	+	+	+	-	-	-	-	-	-
miR-3082	*miR-3082*	-	+	-	-	-	-	-	-	-
miR-3475	*miR-3475*	-	+	-	-	-	-	-	-	-
miR-3535	*miR-3535*	+	-	-	-	-	-	-	-	-
miR-8114	*miR-8114*	+	+	+	-	-	-	-	-	-

*All miRNAs appearing above row 83 represent those that have been identified as being conserved in all epididymal regions of the mouse, rat, and human.

1. Belleannee, C., Calvo, E., Thimon, V., Cyr, D. G., Legare, C., Garneau, L. and Sullivan, R. (2012). Role of microRNAs in controlling gene expression in different segments of the human epididymis. PLoS One 7, e34996.

2. Ma, W., Xie, S., Ni, M., Huang, X., Hu, S., Liu, Q., Liu, A., Zhang, J. and Zhang, Y. (2012). MicroRNA-29a inhibited epididymal epithelial cell proliferation by targeting nuclear autoantigenic sperm protein (NASP). J Biol Chem 287, 10189-99.
